# Calculating the Aqueous pK_a_ of Phenols: Predictions for Antioxidants and Cannabinoids

**DOI:** 10.3390/antiox12071420

**Published:** 2023-07-13

**Authors:** Max Walton-Raaby, Tyler Floen, Guillermo García-Díez, Nelaine Mora-Diez

**Affiliations:** 1 Department of Chemistry, Thompson Rivers University, Kamloops, BC V2C 0C8, Canada; max.walton-raaby@uwaterloo.ca (M.W.-R.); tfloen@tru.ca (T.F.); guillermogd89@gmail.com (G.G.-D.); 2 Department of Chemistry, University of Waterloo, Waterloo, ON N2L 3G1, Canada

**Keywords:** acid dissociation constant, pK_a_, phenols, predictions, antioxidants, cannabinoids, DFT, SMD, PCM

## Abstract

We aim to develop a theoretical methodology for the accurate aqueous pK_a_ prediction of structurally complex phenolic antioxidants and cannabinoids. In this study, five functionals (M06-2X, B3LYP, BHandHLYP, PBE0, and TPSS) and two solvent models (SMD and PCM) were combined with the 6-311++G(d,p) basis set to predict pK_a_ values for twenty structurally simple phenols. None of the direct calculations produced good results. However, the correlations between the calculated Gibbs energy difference of each acid and its conjugate base, ${\Delta G}_{aq(BA)}^{{}^{\circ}}={\Delta G}_{aq\left(A^{-}\right)}^{{}^{\circ}}-{\Delta G}_{aq(HA)}^{{}^{\circ}}$, and the experimental aqueous pK_a_ values had superior predictive accuracy, which was also tested relative to an independent set of ten molecules of which six were structurally complex phenols. New correlations were built with twenty-seven phenols (including the phenols with experimental pK_a_ values from the test set), which were used to make predictions. The best correlation equations used the PCM method and produced mean absolute errors of 0.26–0.27 pK_a_ units and R^2^ values of 0.957–0.960. The average range of predictions for the potential antioxidants (cannabinoids) was 0.15 (0.25) pK_a_ units, which indicates good agreement between our methodologies. The new correlation equations could be used to make pK_a_ predictions for other phenols in water and potentially in other solvents where they might be more soluble.

## 1. Introduction

Acid dissociation constants (K_a_, pK_a_ = −log K_a_) are crucial physico-chemical quantities that impact chemical, environmental, and biochemical research [1,2,3,4,5,6]. Biochemical kinetic and thermodynamic studies involving acids require the calculation of molar fractions or Gibbs free energies of reaction at physiological pH for which aqueous pK_a_ values are necessary [7,8,9,10,11]. Accurate predictions of aqueous pK_a_ values can also be used to predict pK_a_ values in non-aqueous environments [12]. The quest for determining reliable aqueous pK_a_ values for complex phenolic compounds, including cannabinoids, has been motivated by our antioxidant studies on this family of compounds.

Choosing the best methods for obtaining reliable experimental pK_a_ values can be challenging (due to low solubility, difficulty isolating, high reactivity, and variable ionic strength solutions) and time consuming [13,14,15]. Hence, theoretical calculations are a promising alternative. A traditional methodology uses thermodynamic cycles [14,15,16,17,18,19], which combine experimental or calculated ab initio gas phase Gibbs free energies with calculated solution Gibbs free energies. Another approach uses the dissociation equilibrium, HA_(aq)_ ⇌ A^−^_(aq)_ + H^+^_(aq)_, and requires experimental data for H^+^, which is quite variable [14,15,20,21]. Other acid-base equilibria can be applied as well, relative to a reference acid whose experimental pK_a_ is required [22,23]. Alternatively, various linear correlations between calculated properties (in the gas phase or in solution) and experimental pK_a_ values have shown to have important predictive value [24,25,26,27,28]. The application of density functional theory methods combined with continuum solvation methods such as SMD (solvent model based on density) or PCM (polarizable continuum model) is a practical approach for estimating properties in solution. However, in some cases explicit solute molecules are required in addition to the continuum, especially around charged species, to achieve good results [29,30,31].

Phenolic molecules are ubiquitous in the human body, as well as in nature [32]. Examples of endogenous phenolic molecules that play a crucial role are the neurotransmitters serotonin and dopamine and the thyroid hormones and estradiol [33,34]. Other phenolic natural products have made it into the modern-day pharmacopeia: aspirin is sourced from the bark of the willow tree [35], and morphine is an alkaloid present in the opium poppy [36]. Other classes of phenolic molecules include cannabinoids, flavonoids, catechins, and polyphenols, which have shown promising pharmacological properties, including antioxidant activity [32,37].

Previous theoretical studies have focused on the aqueous pK_a_ determination of phenols [23,31,38]. Thapa and Schlegel’s best results include three explicit water molecules surrounding the –OH and –O^–^ groups in the acids and conjugate bases, respectively (HA∙3H_2_O_(aq)_ ⇌ A^−^∙3H_2_O_(aq)_ + H^+^_(aq)_), while working at the B3LYP(SMD)/6-311++G(d,p) level of theory [31]. They achieved mean absolute (MAE) and signed errors (MSE) of 0.45 and −0.02 pK_a_ units, respectively. It is important to note that the set of twenty-five phenols they considered (with pK_a_ values ranging from 7.66 to 10.30) does not include nitrophenols nor 2-substituted phenols, which are compounds we are interested in studying. An earlier study by Galano’s group in 2011 focused on four large phenolic derivatives (acetaminophen, profadol, tapentadol, and ketobemidone) and explored calculations using twenty-two reaction schemes and nine functionals combined with the PCM solvation method (applied through single-point energy calculations) with up to seven explicit solvent molecules [23]. They recommended the reaction scheme HA + OH^−^ (3H_2_O) ⇌ A^−^ (H_2_O) + 3H_2_O, and their best results were obtained with the PBE0 (MAE = 0.77), TPSS (MAE = 0.82), BHandHLYP (MAE = 0.82), and B3LYP (MAE = 0.86) functionals, using the Gaussian03’s PCM implementation in single-point calculations. In the absence of experimental values, calculations were tested relative to theoretical predictions made with the ACD/Laboratories Software [39]. In a newer publication, that came to our attention after our calculations had finished, Galano et al. reported an extensive study (also considering carboxylic acids and amines) that applied 74 levels of theory (all with the SMD solvation model) to a set of twenty simple phenols covering a pK_a_ range from 6.33 to 10.31. Their recommended predictive approach requires the Gibbs energy difference between an acid and its conjugate base and can produce MAE less than 0.35 pK_a_ units for 98.6% of the ten simple phenols they tested [38]. The correlation equations reported, which they have applied in several studies [40,41], are yet to be evaluated with phenols that are more complex for which experimental data exist. We will be referring to the results obtained by these studies and will test their predictive capabilities alongside our work.

**Figure 1:**
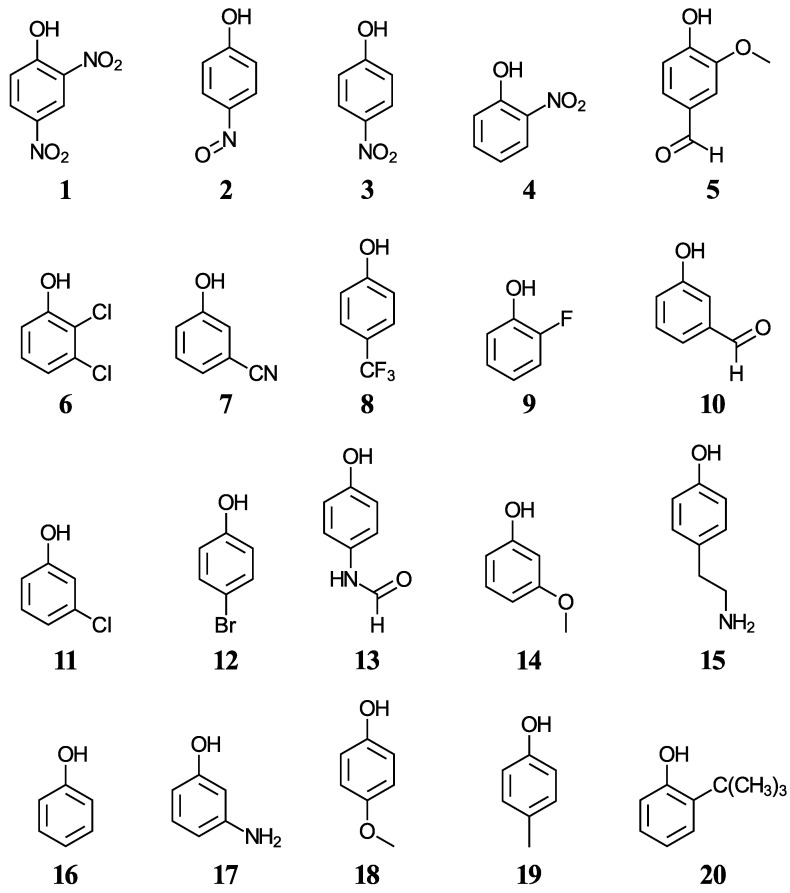
Structures and numeric labels used to identify the phenols in the training set.

We have selected twenty simple phenols ((**1**–**20**), displayed in Figure 1) containing a variety of functional groups with experimental aqueous pK_a_ values in the range from 4.07 to 10.62 [42,43,44], with the objective to develop a methodology for the accurate pK_a_ determination of more complex phenols including cannabinoids. Five functionals, two solvation methods, and three acid-base dissociation equilibria will help us test the accuracy of the direct aqueous pK_a_ calculations. Various correlations to experimental data will also be considered.

**Figure 2:**
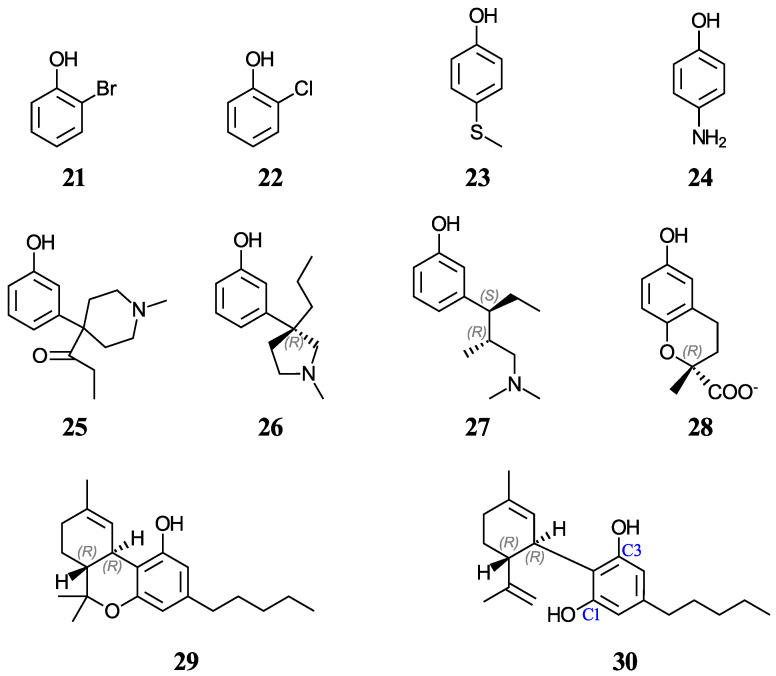
Structures and numeric labels used to identify the phenols in the test set.

**Figure 3:**
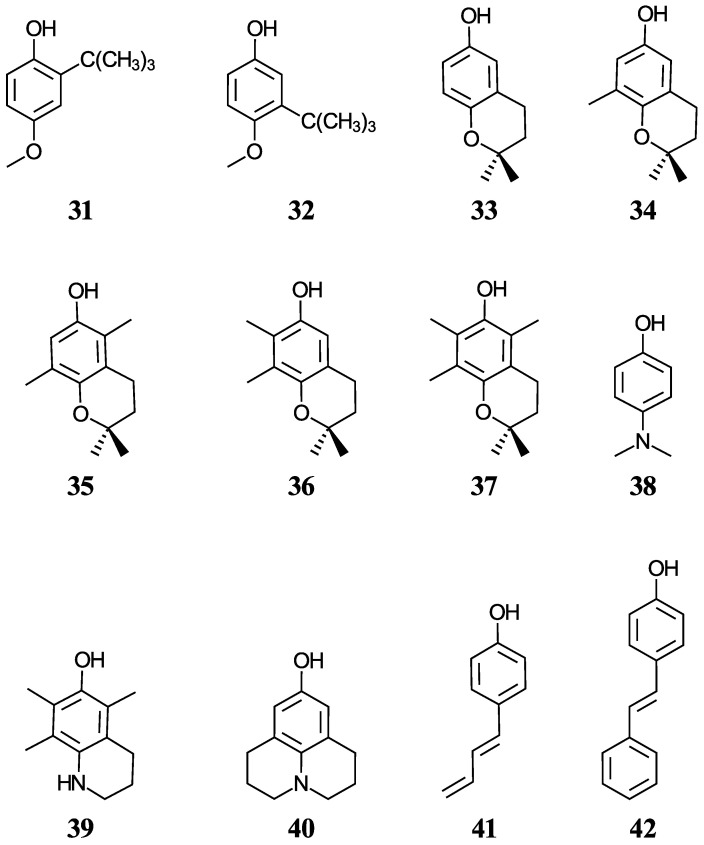
Structures and numeric labels used to identify the phenolic antioxidants studied.

Another group of ten phenols ((**21**–**30**), displayed in Figure 2) is used as an independent test set to compare our predictions to previously reported experimental or theoretical aqueous pK_a_ values. This test group includes six complex phenols. The best methodologies are later used to predict the aqueous pK_a_ values of complex phenols with potential antioxidant properties that are currently under study by our group [45,46,47]. This group of compounds ((**31**–**42**), shown in Figure 3) includes food additives (**31** and **32**) [48], vitamin E analogues (**33**–**37**) which have a methyl group in place of the phytyl tail (C_16_H_33_) due to the tail’s small impact on local properties such as acidity [49], aminophenols (**38**–**40**) used in cosmetics, dyes, and photographic developers [50,51], and stilbenes related to resveratrol (**41** and **42**) [49].

**Figure 4:**
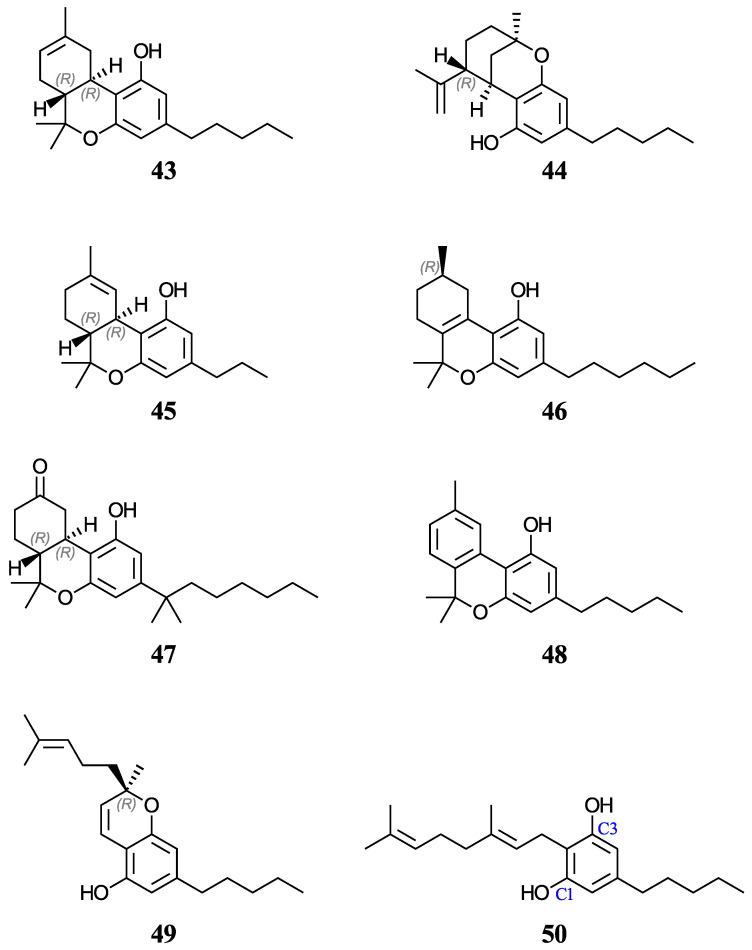
Structures and numeric labels used to identify the cannabinoids studied.

Furthermore, aqueous pK_a_ predictions will also be made for of a set of nine cannabinoids displayed in Figure 4. Cannabinoids are phytochemicals found in the Cannabis plant [52]; nevertheless, this term is also used for any substance which interacts with the endocannabinoid system, including drugs that bear no resemblance to plant-derived cannabinoids [53]. Given the current pharmacological interest, inherent legalities, and little data available for cannabinoids, we thought that it would be appropriate to investigate these molecules that also have a phenol ring in their basic structure. Increasing evidence indicates that certain cannabinoids are effective antioxidants, in addition to their therapeutic uses [54,55,56,57,58,59]. For this study, we have chosen eight phytocannabinoids (**29**, **30**, **43**–**45**, and **48**–**50**), which are important components in the *Cannabis sativa* plant, and two synthetic cannabinoids (**46** and **47**), all of which are being investigated for potential therapeutic uses. The test set includes ∆^9^-tetrahydrocannabinol (∆^9^-THC, **29**) and cannabidiol (CBD, **30**), which have an experimental and a predicted aqueous pK_a_ value reported, respectively. For molecules with stereocenters, labels have been added to identify the stereoisomer used in each case (see Figure 2 and Figure 4), although the calculated pK_a_ is not affected by this.

## 2. Computational Methodology

Calculations were performed with the Gaussian16 software package [60]. Five DFT functionals were applied with the 6-311++G(d,p) basis set: M06-2X, B3LYP, BHandHLYP, PBE0, and TPSSTPSS (referred to as TPSS). These functionals were chosen based on their performance in previous pK_a_ studies of phenols [23,31,38]. The aqueous environment was modelled by two widely used continuum methods, the Integral Equation Formalism Polarizable Continuum Model [61,62,63,64] (IEFPCM, with the solute cavities built by the united atom for Hartree–Fock model; radii = UAHF) [65] and the Solvation Model based on Density (SMD) [66]. The default implementation of IEFPCM (referred to as PCM) in Gaussian16, unlike SMD, neglects explicit non-electrostatic energy contribution terms (e.g., cavity formation, dispersion, and repulsion terms).

Geometry optimizations were followed by a frequency calculation, both including solvent effects, at the same level of theory to determine the nature of the stationary points, and all structures were confirmed to be local minima in which no imaginary frequencies were present. Given that the conformation used for an acid and its conjugate base can greatly affect the calculated pK_a_ value [67], every effort was made to ensure the most stable conformer was optimized in each case. For example, in 2-substituted halogen groups (molecules **6**, **9**, **21**, and **22**), SMD typically favoured the OH facing the halogen while PCM favoured the opposite case. Additionally, the pK_a_ of the (amino) protonated form of molecules **15**, **17**, and **24**–**27** was computed to assess the possibility of concurrent microequilibria, of which none were considered significant. The optimized structures of all the species considered in this study at the M06-2X(SMD)/6-311++G(d,p) level of theory are provided in the Supporting Information. The absolute aqueous Gibbs free energies of the different species considered in this study at the various levels of theory at 298.15 K are reported in Tables S1–S5 of the Supporting Information.

This study explores three aqueous acid-base dissociation equilibria (Reactions (R1)–(R3), shown below as (R1)–(R3)) for calculating the absolute pK_a_ value of an acid, HA, whose conjugate base is A^−^. Reactions (R1) and (R3) are standard ones used in theoretical pK_a_ determinations and, together with Reaction (R2), have produced reasonable results for phenolic molecules [23].

(R1) $$\begin{array}{cc} HA⇌A^{-}+H^{+}\; & {pK}_{a}=\frac{{∆G{}^{\circ}}_{\left(aq\right)}}{RTln(10)} \end{array}$$

(R2) $$\begin{array}{cc} HA+{OH}^{-}\;(3H_{2}O)⇌A^{-}\;(H_{2}O)+3H_{2}O\; & {pK}_{a}=\frac{{∆G{}^{\circ}}_{\left(aq\right)}}{RTln(10)}+14+3\log[H_{2}O] \end{array}$$

(R3) $$\begin{array}{cc} HA+{Ref}^{-}⇌A^{-}+HRef\; & {pK}_{a}=\frac{{∆G{}^{\circ}}_{\left(aq\right)}}{RTln(10)}+{pK}_{a}(HRef) \end{array}$$

Reaction (R1) requires an experimental aqueous Gibbs free energy value for H^+^ to be combined with the calculated aqueous G° values of HA and A^−^. Even though several values have been reported and used in calculations, we have chosen to work with −270.29 kcal/mol [20,23,30,31,38], because it has been corrected for the 1 M reference state and it has sometimes led to a good reproducibility of experimental aqueous pK_a_ values.

Reaction (R2) includes explicit water molecules solvating the anions, which attempts to simulate the solute–solvent interactions that are not fully modelled in the implicit solvation models used. Hydrogen bonding between a water molecule and the conjugate base in A^−^ (H_2_O) can significantly stabilize the charged species. The species OH^−^ (3H_2_O) is the OH^−^ ion solvated by three water molecules, while the species indicated as 3H_2_O is a water cluster of three molecules. The concentration of water used is 55.55 mol/L [23].

Reaction (R3) involves a reference acid similar in structure to the acid studied for which a reliable pK_a_ value is available. For all molecules except phenol, phenol was used as the reference acid (experimental pK_a_ = 9.99). For the determination of phenol, 3-methoxyphenol was used as the reference acid (experimental pK_a_ = 9.65).

The uneven distribution of charges across the equilibrium of Reaction (R1) makes this approach more prone to errors. Reactions (R2) and (R3) do not encounter this difficulty since both sides of their equilibria are balanced in terms of charges, thus contributing to better results due to the cancellation of errors. Reactions (R1)–(R3) contain the same number of computed reactant and product species; thus, reference state conversions are not needed.

Molecules **30** and **50** both contain two equivalent deprotonation sites through rotation of the sigma bond connecting the benzene ring to its substituent. As a result, the acid equilibrium constant should be doubled [68]. Accordingly, pK_a_ values for molecules **30** and **50** must be corrected by subtracting log(2) from their respective calculated values using Reactions (R1)–(R3) or the correlation equations [38]. The deprotonation of both sites was tested, and the most stable conformer was used in each case.

## 3. Results and Discussion

### 3.1. Exploring Several Methodologies for the Direct Calculation of pK_a_ Values

Eleven phenols of varying structural complexities are used to initially test the aqueous pK_a_ calculations using five functionals, two solvation models, and three acid–base equilibria. The training set is then increased to twenty phenols (see Figure 1) for all functionals other than PBE0 because of significant technical issues. Mean absolute errors (MAEs), used to assess prediction quality, have been compiled in Table 1. The direct calculation results using the SMD solvation method are always much better than the corresponding results using PCM in each case considered, except when using the M06-2X and TPSS functionals with Reaction (R2). While there are differences between the results obtained with the different functionals using each reaction scheme, the reaction scheme used is much more impactful on the overall accuracy of the methodology applied, and that is how we have organized the discussion that follows.

#### 3.1.1. Results Obtained from the Direct Application of Reactions (R1) and (R2)

The results obtained with Reactions (R1) and (R2) are not satisfactory. The calculated individual pK_a_ values and their errors are displayed in Tables S6 and S7 (Tables S8 and S9) of the Supporting Information when using Reaction (R1) (Reaction (R2)). The MAE ranged between 3.09 and 5.24 (4.73 and 7.38) pK_a_ units when using Reaction (R1) (Reaction (R2)) and the SMD solvation method for the set of twenty (eleven) phenols. Larger errors were usually obtained with the PCM solvation method, between 4.76 and 7.05 (4.75 and 6.83) pK_a_ units when using Reaction (R1) (Reaction (R2)). With very few exceptions, the calculated aqueous pK_a_ values were always overestimated (with positive errors).

It is worth mentioning that Reaction (R1) was shown to drastically improve the pK_a_ calculations for phenolic molecules (as well as alcohols, hydroperoxides, and thiols) when three explicit water molecules are added to the HA and A^−^ species, while using the value of −270.29 kcal/mol for the aqueous Gibbs energy of H^+^ after corrections [30,31]. This approach produced MAE of 0.45 pK_a_ units for phenols at the best level of theory reported, B3LYP(SMD)/6-311++G(d,p) [31]. The M06-2X errors were still significantly large using this approach with mean signed errors of −1.40 pK_a_ units.

Reaction (R2), using the Gaussian03 implementation of PCM through single-point energy calculations, was reported to produce much smaller MAE values which were generally in the 0.77–0.86 pK_a_ unit range using similar functionals [23]. Our approach, including PCM as implemented in Gaussian16 in both geometry optimizations and frequency calculations, leads to much larger errors for direct pK_a_ predictions. Given the results obtained for eleven phenols using Reaction (R2), which requires the additional calculation of the explicitly solvated conjugate base, we decided not to take it into account any further.

#### 3.1.2. Results Obtained from the Direct Application of Reaction (R3)

The best direct calculation results are obtained using Reaction (R3), and M06-2X was the best-performing functional with both solvation models. The calculated individual pK_a_ values and their errors are displayed in Table 2 and Table S10, respectively. Table 2 also displays the experimental values used. The best predictive methodologies are expected to have the lowest MAEs and mean signed errors (MSEs, taking their sign into account). When considering the training set of twenty phenols, MAEs between 1.34 and 1.61 (between 1.99 and 2.28) pK_a_ units are obtained when using SMD (PCM).

In general, direct calculations with Reaction (R3) led to underestimated (overestimated) pK_a_ values for compounds more (less) acidic than phenol. The more acidic the phenol, the larger and more negative the error in these calculations. The calculated pK_a_ values for the three nitrophenols and nitrosophenol, compounds **1**–**4**, have the largest MAEs (e.g., between −3.36 and −5.02 pK_a_ units with M06-2X(SMD)). These results indicate that the direct methodology tested is insufficient to predict nitrophenols.

When the calculated pK_a_ values of compounds **1**–**4** are excluded, the MAEs become significantly reduced with values between 0.50 and 0.94 with SMD (between 0.90 and 1.38 with PCM), and MSE between −0.09 and −0.41 (−0.61 and −0.88) when using SMD (PCM). The PBE0 functional seems to produce the best results, even though only eleven of the twenty phenols were calculated because of significant technical issues when applying this functional, with MAEs and MSEs of 0.50 and −0.09 (0.90 and −0.61), respectively, with SMD (PCM). Ignoring the incomplete PBE0 results, BHandHLYP(SMD) produces the best results with an MAE and MSE of 0.74 and −0.23, respectively, followed by M06-2X(SMD) with an MAE of 0.78 and MSE of −0.33. Searching for smaller MAE values in the calculated aqueous pK_a_ values of phenols, various correlations between the calculated ${∆G{}^{\circ}}_{\left(aq\right)}$ values and the experimental aqueous pK_a_ values are investigated.

### 3.2. Exploring Various Correlations with Experimental pK_a_ Values and the Training Set

Correlations between calculated properties (descriptors), including ΔG° values related to the acid dissociation equilibrium, and experimental aqueous pK_a_ values have been previously reported [12,24,25,26,27,28,38,69,70]. When a correlation has a significantly large R^2^ value and the MAE is very small, the fitted equation can be directly used to predict new aqueous pK_a_ values of similar compounds.

Correlations between the experimental aqueous pK_a_ values and the calculated ${∆G{}^{\circ}}_{\left(aq\right)}$ for Reactions (R1)–(R3) produced very high R^2^ values (>0.90) in all but one case and much smaller and more consistent MAEs when the corresponding correlation equation is used at a given level of theory (see Table 1). Ignoring the results for Reaction (R2) and the PBE0 functional (which only considers eleven phenols in the training set and leads to slightly larger MAEs in most cases), MAEs between 0.22 and 0.27 (0.28 and 0.40) are obtained with SMD (PCM), which is an excellent result since a pK_a_ error of 0.50 units corresponds to a 0.68 kcal/mol error in the calculated ${∆G{}^{\circ}}_{\left(aq\right)}$.

Given that when calculating ${∆G{}^{\circ}}_{\left(aq\right)}$ for Reactions (R1) and (R3) the only variables are G°(A^−^) and G°(HA), we decided to directly focus the correlation between the experimental aqueous pK_a_ values and their difference, ${\Delta G}_{aq(BA)}^{{}^{\circ}}={\Delta G}_{aq\left(A^{-}\right)}^{{}^{\circ}}-{\Delta G}_{aq\left(HA\right)}^{{}^{\circ}}$. The calculated pK_a_ values and their errors for each of the twenty phenols in the training set, relative to the corresponding correlation equation, are displayed in Table 3 and Table S11, respectively, for the various levels of theory considered (only eleven phenols were calculated with the PBE0 functional). The MAEs for these correlations are shown in Table 1, and the associated correlation equations and R^2^ values are listed in Table 4.

The correlations using the SMD solvation model have lower MAEs and better R^2^ values than the equivalent correlations using PCM, which is the same trend seen from the direct calculations previously discussed (see Table 4). The MAE values between 0.22 and 0.27 (0.28 and 0.40) and the R^2^ values between 0.947 and 0.975 (0.898 and 0.946) when using SMD (PCM) are excellent results. M06-2X(SMD) produced the most promising results with an MAE of 0.22 (and R^2^ = 0.975); likewise, this functional also produced the best results using PCM with an MAE of 0.28 (and R^2^ = 0.946).

**Figure 5:**
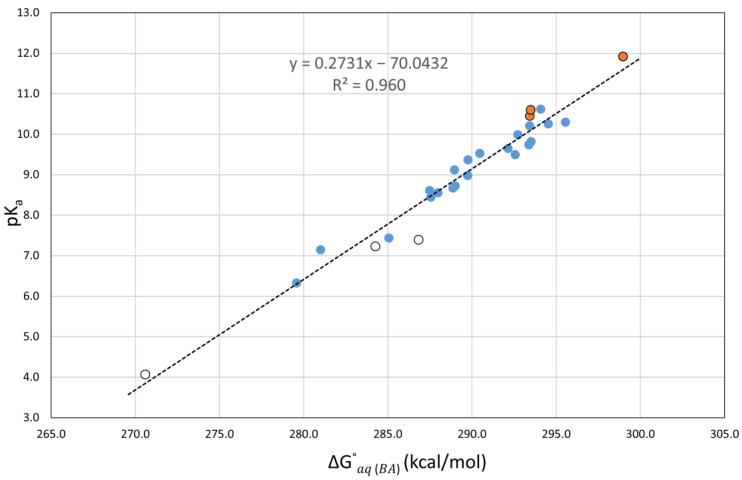
Plot of the experimental aqueous pK_a_ values versus the calculated ${\Delta G}_{aq(BA)}^{{}^{\circ}}$ at the M06-2X(PCM)/6-311++G(d,p) level of theory for 27 phenolic molecules.

It is important to note that when using these correlations (see Table 3), the calculated errors for the nitrophenols (**1**,**3**,**4**) and the nitrosophenol (**2**), which were very large when considering direct pK_a_ calculations, are very small, in agreement with the calculated errors for the other compounds. This indicates that our correlations correctly adjust for the previously underestimated pK_a_ predictions. Another observation is that the molecules with intramolecular hydrogen bonding affecting the most stable conformation of the acid form (**1**,**4**,**5**) tend to have a slightly higher error, as seen in Figure 5. We suspect that the additional stabilization in the acidic form may lead to an underestimated pK_a_ prediction.

Correlations between the experimental aqueous pK_a_ values and ${\Delta G}_{aq(BA)}^{{}^{\circ}}$ were reported for simple phenols by Galano et al. at several levels of theory using only the SMD solvation method [38]. Their training set of twenty simple phenols covered the experimental pK_a_ range from 6.33 to 10.31. Using their reported correlation equations, we calculated slightly larger MAE (from 0.21 to 0.46) for the compounds in our training set (see Table 1). Apart from the fact that our correlation was built from these data, this difference is possibly due to the larger number of molecules more acidic than phenol in our training set, compared to theirs.

### 3.3. Predicting Aqueous pK_a_ Values of Complex Phenols

A significant number of phenolic molecules that we are interested in studying (see Figure 2, Figure 3 and Figure 4) are of greater structural complexity than the twenty molecules included in our training set. Hence, it is essential to test the performance of our correlation equations with more complex phenols. This is a largely underexplored area, partly because most complex phenols lack experimental aqueous pK_a_ values and no previous theoretical studies have verified the quality of aqueous pK_a_ predictions for complex phenols using reliable experimental data.

#### 3.3.1. Checking the Predictions with a Test Set

To check the accuracy of our correlations from the training set of twenty simple phenols, we collected ten phenolic molecules of varying complexity (displayed in Figure 2). Seven of them have experimental aqueous pK_a_ values reported (**21**–**24** and **27**–**29**) [42,71,72], and three other ones (**25**, **26**, and **30**) only have predicted values; **25** and **26** were predicted by the ACD/Laboratories Software [39], and **30** has a minimum experimental aqueous pK_a_ value reported [73]. However, the same experimental methodology approximated the pK_a_ of **29** within 0.1 pK_a_ units [73]. While phenols **21**–**24** are simple, phenols **25**–**30** are of significant structural complexity, and **27**–**29** have experimental pK_a_ values reported. Hence, for the first time to our knowledge, we will be assessing aqueous pK_a_ predictions of complex phenols using correlations involving the experimental values of simpler ones.

**Table 4:** Details of the correlation equations obtained for the initial training set of 20 phenols and for the final training set of 27 phenols ^a,b^.

Fitted Equation	${\mathrm{pK}}_{a\;(\exp)}=m{\Delta G}_{aq(BA)}^{{}^{\circ}}+n$			
Level of Theory	m	n	R^2^	MAE
**Set of 20 phenols**				
M06-2X(SMD)	0.3533	−92.4756	0.975	0.22
B3LYP(SMD)	0.3266	−84.9381	0.958	0.24
BHandHLYP(SMD)	0.3305	−86.9380	0.963	0.25
PBE0(SMD) ^d^	0.3761	−99.8596	0.969	0.22
TPSS(SMD)	0.3315	−86.3857	0.947	0.27
M06-2X(PCM)	0.2988	−77.5916	0.946	0.28
B3LYP(PCM)	0.2751	−70.7109	0.916	0.34
BHandHLYP(PCM)	0.2847	−74.4297	0.938	0.30
PBE0(PCM) ^d^	0.3328	−88.0977	0.935	0.36
TPSS(PCM)	0.2789	−71.8317	0.898	0.40
**Set of 27 phenols ^c^**				
M06-2X(SMD)	0.3244	−84.1492	0.953	0.27
B3LYP(SMD)	0.3071	−79.2803	0.955	0.26
BHandHLYP(SMD)	0.3039	−79.2127	0.950	0.27
TPSS(SMD)	0.3104	−80.2526	0.959	0.27
M06-2X(PCM)	0.2731	−70.0432	0.960	0.26
B3LYP(PCM)	0.2522	−63.9968	0.956	0.27
BHandHLYP(PCM)	0.2581	−66.5540	0.957	0.26
TPSS(PCM)	0.2489	−62.9995	0.938	0.32

^a^ ${\Delta G}_{aq(BA)}^{{}^{\circ}}={\Delta G}_{aq\left(A^{-}\right)}^{{}^{\circ}}-{\Delta G}_{aq(HA)}^{{}^{\circ}}$, new pK_a_ values should be calculated as pK_a (calc)_ = $m{\Delta G}_{aq(BA)}^{{}^{\circ}}+n$; ^b^ Using the 6-311++G(d,p) basis set; ^c^ The 7 phenols of the test set (which includes 3 large phenols) with experimental pK_a_ values (see Table 5) have been added to the initial set of 20 phenols; ^d^ Calculated using 11 phenols.

Using the correlation equations (reported in Table 4) for the training set of twenty phenols employing the functionals M06-2X, B3LYP, BHandHLYP, and TPSS (with both the SMD and PCM solvation methods), the aqueous pK_a_ values of the training set are calculated. Experimental pK_a_ values and errors in their prediction, with MAE and MSE values, are shown in Table 5, while the calculated pK_a_ values are reported in Table S12. MAE and MSE values are reported for the seven phenols with experimental data and for the entire set (including predicted values). Predictions using the correlation equations of Galano et al. [38] and employing the method suggested by Thapa and Schlegel [31] are also reported for comparison.

Unlike the trends seen in the MAE and MSE values previously reported (see, for example, Table 2 and Table 3), the correlations using SMD usually exhibit larger errors in the predicted pK_a_ values of the test set than when using PCM. The lowest MAE of 0.24 using the whole test set is achieved with M06-2X(PCM); however, when only experimental pK_a_ values are used, the lowest MAE of 0.23 is produced by B3LYP(PCM). The MSE values are very similar between both solvation models and indicate that our correlations slightly underestimate the test set’s pK_a_ values.

Comparing our SMD results with Ref. [38], our values almost always produce smaller MAEs and MSEs. To compare with the predictive ability of the method reported in Ref. [31], a few molecules in our test set were selected (**24**, **28,** and **29**). In all cases (except for (R)-Trolox using B3LYP(SMD) and TPSS(SMD)), our correlations produce more accurate values. Moreover, the method reported in Ref. [31] is incompatible with 2-chlorophenol (and in general, with 2-substituted phenols) since the water molecules would not equilibrate around the -OH group in the acid species. Given that the pK_a_ values of nitrophenols are difficult to predict directly, we included 2-nitrophenol in Table 5 (experimental pK_a_ = 7.23) [42]. The pK_a_ predictions of 6.13 and 3.92 by Refs. [31,38], respectively, had much larger errors than when using our M06-2X(PCM) correlation (7.34, see Table S12).

**Table 5:** Predicted pK_a_ errors and experimental pK_a_ values for the phenols in the test set (**21**–**30**) using the corresponding pK_a (exp)_ vs. ${\Delta G}_{aq(BA)}^{{}^{\circ}}$ correlation equation listed in Table 4 (obtained from a training set of 20 phenols) at several levels of theory at 298.15 K ^a^.

Solvation Method	SMD				PCM				Exp ^f^	Other Predictions	
Name/Functional	M06-2X	B3LYP	BHandHLYP	TPSS	M06-2X	B3LYP	BHandHLYP	TPSS		Ref. [38] ^g^	Ref. [31] ^h^
**(21)** 2-bromophenol	0.49	0.56	0.58	0.48	−0.12	0.06	−0.04	0.08	8.45 ^i^	0.49	
**(22)** 2-chlorophenol	−0.14	−0.06	−0.09	−0.11	−0.11	0.00	−0.07	−0.69	8.56 ^i^	−0.10	
**(23)** 4-(methylthio)phenol	−0.13	−0.14	−0.01	0.27	−0.34	−0.34	−0.35	−0.97	9.53 ^i^	−0.19	
**(24)** 4-aminophenol	0.35	0.33	0.33	0.30	0.42	0.39	0.46	0.41	10.30 ^i^	0.17	0.43 ^l^
**(25)** ketobemidone	−0.17	0.16	−0.07	0.12	−0.02	0.98	1.12	0.96	[9.96] ^m^	−0.26	
**(26)** profadol	−0.24	−0.19	−0.26	−0.22	−0.05	0.00	0.04	−0.01	[10.27] ^m^	−0.36	
**(27)** tapentadol	−0.36	−0.29	−0.32	−0.31	−0.36	−0.37	−0.35	−0.33	10.45 ^n^, [10.09] ^m^	−0.48	
**(28)** (R)-Trolox	−0.40	−0.81	−0.37	−0.59	−0.18	0.08	0.09	0.08	11.92 ^j^	−0.67	−0.47
**(29)** ∆^9^-tetrahydrocannabinol (Δ^9^-THC)	−0.83	−0.46	−0.66	−0.57	−0.50	−0.40	−0.43	−0.41	10.60 ^n^	−0.91	−1.37
**(30)** cannabidiol (CBD) ^d^	−0.08	0.29	0.18	0.18	0.30	0.26	0.29	0.26	9.7 ^k^	−0.18	
**(4)** 2-nitrophenol	−0.38	−0.12	−0.56	0.15	0.11	0.32	0.31	0.63	7.23 ^h^	−1.10	−3.31
**MAE** ^b^ (test set with exp values)	**0.39**	**0.38**	**0.34**	**0.38**	**0.29**	**0.23**	**0.25**	**0.42**			
**MSE** ^b^	−0.15	−0.12	−0.08	−0.08	−0.17	−0.08	−0.10	−0.26			
**MAE** ^c^ (entire test set)	**0.32**	**0.33**	**0.29**	**0.32**	**0.24**	**0.29**	**0.32**	**0.42**			
**MSE** ^c^	−0.15	−0.06	−0.07	−0.05	−0.10	0.06	0.08	−0.06			
**MAE** (Ref. [38]) ^e^	**0.43**	**0.40**	**0.35**	**0.42**							
**MSE** (Ref. [38]) ^e^	−0.24	−0.08	−0.09	−0.26							
**MAE** ^b,o^ (complex ph. with exp values)	**0.53**	**0.52**	**0.45**	**0.49**	**0.35**	**0.28**	**0.29**	**0.27**			
**MSE** ^b,o^	−0.53	−0.52	−0.45	−0.49	−0.35	−0.23	−0.23	−0.22			
**MAE** ^c,o^ (all complex ph.)	**0.35**	**0.37**	**0.31**	**0.33**	**0.23**	**0.35**	**0.39**	**0.34**			
**MSE** ^c,o^	−0.35	−0.22	−0.25	−0.23	−0.14	0.09	0.13	0.09			
**MAE** (Ref. [38]) ^e,o^	**0.59**	**0.55**	**0.51**	**0.77**							
**MSE** (Ref. [38]) ^e,o^	−0.59	−0.55	−0.51	−0.77							

^a^ The calculated aqueous pK_a_ values are reported in Table S12; ^b^ Mean absolute (MAE) and signed (MSE) errors only taking (7) experimental values into account; ^c^ MAE and MSE taking (7) experimental and (3) previously predicted values (shown in square brackets) into account; ^d^ Macroscopic pK_a_ values have been calculated by accounting for the degenerate deprotonation sites; ^e^ Using the correlation equations reported (SMD only) for the compounds with experimental data; ^f^ Values in brackets are theoretical predictions; ^g^ Predicted pK_a_ values using the correlation equation at the M06-2X(SMD) level of theory; ^h^ Predicted pK_a_ values using the three-water clusters for the acid and the conjugate base as done in Ref. [31]; ^i^ Ref. [42]; ^j^ Ref. [72]; ^k^ Ref. [73] (minimum experimental value); ^l^ Value taken from Ref. [31]; ^m^ Ref. [39]; ^n^ Ref. [71]; ^o^ Taking only the complex phenols into account (**25**–**30**).

The more complex phenols (**25**–**30**) were isolated from the simpler phenols (**21**–**24**), and the complex phenols with experimental aqueous pK_a_ values (**27**–**30**) were further separated from those with estimated values. When considering all complex phenols in the test set, the MAEs ranged between 0.31 and 0.37 (0.23 and 0.39) with SMD (PCM). PCM generally performs better than SMD, with the lowest MAE of 0.23 achieved with the M06-2X(PCM) functional.

When considering only the complex phenols with experimental aqueous pK_a_ values, the MAEs increased with SMD, but they generally decreased with PCM. Moreover, the MSE values increased for all functionals in both solvation models when only considering the complex phenols with experimental values. The MAEs ranged between 0.45 and 0.53 (0.27 and 0.35) with SMD (PCM). B3LYP(PCM), BHandHLYP(PCM), and TPSS(PCM) produce small MAE values for the complex phenols with experimental pK_a_ values of 0.28, 0.29, and 0.27 pK_a_ units, while their MSE values are −0.23, −0.23, and −0.22 pK_a_ units, respectively. Compared to Ref. [38], our correlations produced significantly lower MAE and MSE values, especially with PCM. The performance disparity between the SMD and PCM solvent models can in part be attributed to the PCM exclusion of explicit non-electrostatic energy contributions. The excellent results with the test set, especially when using PCM, indicate that our correlations can be confidently applied to our prediction sets.

To increase the statistical value of our work, we included the seven phenols with experimental aqueous pK_a_ values from our test set into new correlated equations at all levels of theory. The best correlation graph obtained for the twenty-seven phenols is shown in Figure 5. The associated equations that will be used for predictions are listed in the second half of Table 4. While the MAEs of the new correlations with SMD slightly increased between 0.02 and 0.05 pK_a_ units when the seven phenols were added, the MAEs with PCM decreased between 0.02 and 0.08 pK_a_ units. The MAE range with twenty-seven phenols became 0.26–0.27 (0.26–0.32) using SMD (PCM), with the lowest MAE of 0.26 shared by M06-2X(PCM) and BHandHLYP(PCM). These are excellent results.

#### 3.3.2. Predicting Aqueous pK_a_ Values of Phenols with Potential Antioxidant Activity

Our group has studied the antioxidant properties of molecules **31**–**42**, shown in Figure 3, and molecule **24**, to repair damaged leucine residues under physiological conditions (pH 7.4). We require accurate pK_a_ values to understand the biological mechanisms of action of these potential antioxidants. Additionally, we provide pK_a_ predictions for molecules **25** and **26** which are opioid analgesics. Table 6 presents the aqueous pK_a_ predictions using PCM, while additional predictions using SMD are displayed in Table S13. The predicted aqueous pK_a_ values could be used as reference values for approximate pK_a_ predictions in other computationally available implicit solvents, as previously reported [12]. This potential solvent transferability is highly useful as many of the species in the prediction sets have poor aqueous solubility.

Molecules **25**–**26** and **31**–**42** exhibit similar acidity, with the majority being lipophilic, with average pK_a_ predictions using PCM (SMD) between 9.15 and 11.51 (9.21 and 11.29). This is unsurprising since most of them share structural features (e.g., heteroatom at the 4-substituted position). The spread of the predicted pK_a_ values for each of these systems using PCM (SMD) was 0.40 (0.52) pK_a_ units or less, with the exception of **25** with PCM predictions spreading up to 1.01 pK_a_ units.

Molecule **25** is predicted to have a pK_a_ of 9.96 [39], in good agreement with the prediction from Ref. [38] of 9.89. On the other hand, our average prediction using PCM is 10.66, but the M06-2X(PCM) prediction is 9.96, and the overall SMD predicted average is 9.89. Our predictions for this molecule are the most spread of all with PCM and the one with the largest average difference (0.77 pK_a_ units) between PCM and SMD. Conversely, molecule **26** was predicted to have a pK_a_ of 10.27 [39], which is in good agreement with the prediction from Ref. [38] of 10.00 and in excellent agreement with our average prediction of 10.25. The predicted values for **25**–**27** used the same software [39], and while the predicted value for **27** was 10.09, the experimental value reported is 10.45 [71].

To our knowledge, there are no previous pK_a_ predictions that we can compare our results with for molecules **31**–**42**. However, the small average (median) range of 0.15 (0.12) for values predicted with M06-2X, B3LYP, BHandHLYP, and TPSS functionals using PCM is a promising result. Evidently, there is good agreement between these levels of theory.

#### 3.3.3. Predicting Aqueous pK_a_ Values of Cannabinoids

Finally, we used the same methodologies to predict the pK_a_ for nine phenolic cannabinoids, shown in Figure 4. Of these, **43**–**46** and **49** present stereoisomerism. In all cases, the naturally occurring isomer was used (see Figure 4). In addition, two synthetic cannabinoids (**47** and **48**) were considered. Compound **46** contains only one stereocenter at C9, which gives a pair of enantiomers. For consistency, the *R* enantiomer was used in each case. In the case of **47**, which is usually commercialized as a racemic mixture of (*S*,*S*)-(+) and (*R*,*R*)-(−) isomers, the latter was used in our calculations. 

We could only find a reliable experimental pK_a_ value for molecule **29** and an estimated minimum experimental value for **30** [71,73]. These cannabinoids were part of the test set previously discussed. Our average PCM prediction for **29** is 10.16 (with a spread of 0.09 pK_a_ units, see Table 6), which is in good agreement with the reported experimental value of 10.60 [71]. The pK_a_ predictions for **29** using the methodologies described in Refs. [31,38] were 9.23 and 9.69, respectively. Similarly, our average PCM pK_a_ prediction for molecule **30** is 9.96, which is also in good agreement with the minimum estimated experimental value of 9.7 [73]. Using the methodology of Ref. [38], the pK_a_ prediction of 9.55 for **30** seems to be slightly underestimated. Further, these results give us confidence in the accuracy of the methodology followed to calculate the macroscopic pK_a_ value for molecule **50**.

Again, due to the structural similarities of the cannabinoids, the average PCM (SMD) predicted pK_a_ range for these molecules was between 9.21 and 10.31 (8.98 and 10.22). An interesting structural trend between molecules **46**, **48**, and **49**, when compared to the other cannabinoids, is their lower expected pK_a_ values (with values of 9.87, 9.26, and 9.21, respectively, if using average PCM predictions; see Table 6) because of the increased conjugate base stabilization from substituent conjugation. All of the cannabinoids were predicted to be similar or slightly more acidic than molecule **29**, if considering its experimental value of 10.60; this is supported by molecule **43**, an isomer of **29**, having an average predicted pK_a_ of 10.15 using PCM (just 0.01 pK_a_ units from **29**’s average prediction).

The spread of the predicted pK_a_ values for each of these systems using PCM (SMD) was 0.51 (0.69) pK_a_ units or less, with the exception of **45** with PCM predictions spreading over 0.86 pK_a_ units. Similar to what was reported in the previous section, the PCM average (median) range between the levels of theory for each molecule was 0.25 (0.17) which gives us confidence in our pK_a_ predictions for these molecules. Furthermore, since the pK_a_ values of cannabinoids were well reproduced in the test set, our prediction methodology could be extended to other molecules of this family.

## 4. Conclusions

Working with an initial training set of eleven structurally simple phenols, which was later expanded to twenty molecules, direct aqueous pK_a_ calculations (using three acid dissociation equilibria) were perform with of five DFT functionals (M06-2X, B3LYP, BHandHLYP, PBE0, and TPSS), using the 6-311++G(d,p) basis set and the SMD and PCM solvent models. Much better and more consistent results were produced from the correlations between the calculated Gibbs energy difference between each acid and its conjugate base, ${\Delta G}_{aq(BA)}^{{}^{\circ}}={\Delta G}_{aq\left(A^{-}\right)}^{{}^{\circ}}-{\Delta G}_{aq(HA)}^{{}^{\circ}}$, and the experimental aqueous pK_a_ values, as previously reported [38]. The correlations using SMD (PCM) produced MAEs between 0.22 and 0.27 (0.28 and 0.40) and R^2^s between 0.947 and 0.975 (0.898 and 0.946). In general, the correlations using twenty phenols with SMD produced more accurate results than PCM.

A new set of ten phenols of varying complexities with experimental and/or predicted pK_a_ values (separated accordingly) was used to test the performance of our correlations. In this case, PCM performed significantly better than SMD and the theoretical methodologies previously reported [31,38] for the entire test set and when the complex phenols were isolated. The best performance (for the set with experimental pK_a_ values) was achieved by B3LYP(PCM) with an MAE (MSE) of 0.23 (−0.08) pK_a_ units. The best performance for the complex phenols with experimental values were achieved by B3LYP(PCM), BHandHLYP(PCM), and TPSS(PCM) with MAE values of 0.28, 0.29, and 0.27 pK_a_ units, respectively. These three functionals are expected to produce the most accurate pK_a_ predictions when combined with the PCM solvent model; however, we have included the remaining levels of theory to form a range of predicted values. Furthermore, we developed new correlations, including the seven molecules from the training set (working with twenty-seven phenols in total) to increase the statistical value of our work. The best MAE for the new correlations was shared by M06-2X(PCM), B3LYP(SMD), and BHandHLYP(PCM) with an MAE of 0.26 and R^2^s between 0.955 and 0.960 (see Table 4).

Our correlations were used to predict the pK_a_ values of twelve molecules with potential antioxidant activity and of nine phenolic cannabinoids. The average prediction range with the PCM (SMD) solvation model was 0.15 (0.21) and 0.25 (0.34) pK_a_ units, respectively, which indicates a very good agreement between our methodologies. These aqueous pK_a_ predictions could be used as reference values for predictions in other solvents [12]. In the future, when more experimental data are available, it would be ideal to extend these correlations to a larger set of complex phenolic molecules to create an even better pK_a_ predictive tool.

## Figures and Tables

**Table 1:** Summary of mean absolute pK_a_ errors (MAE) for the phenols in the training set (obtained from direct calculations and using the correlations between the experimental pK_a_ values and calculated ΔG° values) at several levels of theory using Reactions (R1)–(R3) at 298.15 K.

Solvent Model	SMD					PCM				
Reaction Used	M06-2X	B3LYP	BHandHLYP	PBE0 ^e^	TPSS	M06-2X	B3LYP	BHandHLYP	PBE0 ^e^	TPSS
1 (direct)	3.09	3.43	5.24	4.66	3.48	4.76	4.77	7.05	6.15	4.84
1 (corrected) ^b^	0.22	0.24	0.25	0.22	0.27	0.28	0.34	0.30	0.36	0.40
2 (direct)	5.75	5.89	5.22	4.73	7.38	4.75	6.58	5.43	5.27	6.83
2 (11-set, corrected) ^b^	0.20	0.33	0.28	0.33	0.38	0.30	0.47	0.35	0.48	0.47
3 (direct)	1.43	1.61	1.51	1.34	1.61	2.01	2.28	2.15	1.99	2.23
3 (direct, excl. NO, NO_2_) ^a^	0.78	0.88	0.74	0.50	0.94	1.16	1.38	1.25	0.90	1.37
3 (corrected) ^b^	0.22	0.24	0.25	0.22	0.27	0.28	0.34	0.30	0.36	0.40
${\Delta G}_{aq(BA)}^{{}^{\circ}}$ (20-set, corrected) ^c^	0.22	0.24	0.25	0.22	0.27	0.28	0.34	0.30	0.36	0.40
${\Delta G}_{aq(BA)}^{{}^{\circ}}$ (27-set, corrected) ^c^	0.27	0.26	0.27		0.27	0.26	0.27	0.26		0.32
Using Ref. [38] (20-set) ^d^	0.22	0.26	0.27	0.46	0.21					
Using Ref. [38] (27-set) ^d^	0.26	0.30	0.29		0.26					

^a^ MAE calculated excluding the results for the nitrophenols and nitrosophenol (**1**–**4**); ^b^ Corrected values obtained using the corresponding correlation equation: pK_a (exp)_ vs. ${\Delta G}_{aq(BA)}^{{}^{\circ}}$; ^c^ Corrected values obtained using the corresponding correlation equation between experimental pK_a_ values and the calculated difference ${\Delta G}_{aq(BA)}^{{}^{\circ}}={\Delta G}_{aq\left(A^{-}\right)}^{{}^{\circ}}-{\Delta G}_{aq(HA)}^{{}^{\circ}}$ for the phenols in the training set; ^d^ Using the pK_a (exp)_ vs. ${\Delta G}_{aq(BA)}^{{}^{\circ}}$ correlation equations reported; ^e^ Values reported for 11 phenols.

**Table 2:** Experimental aqueous pK_a_ values [49,50,51] and calculated errors (MAE and MSE) at several levels of theory using Reaction (R3) (HRef = phenol) for direct calculations at 298.15 ^a,b^.

Solvent Model		SMD					PCM				
Name/Functional	Exp. pK_a_	M06-2X	B3LYP	BHandHLYP	PBE0 ^g^	TPSS	M06-2X	B3LYP	BHandHLYP	PBE0 ^g^	TPSS
**(1)** 2,4-dinitrophenol	4.07 ^d^	−5.02	−5.13	−5.37	−4.26	−4.69	−6.55	−6.40	−7.08	−5.53	−5.92
**(2)** 4-nitrosophenol	6.33 ^e^	−3.85	−5.12	−4.47		−5.25	−5.97	−7.29	−6.49		−7.59
**(3)** 4-nitrophenol	7.15 ^d^	−3.77	−4.71	−4.33	−3.87	−4.83	−5.74	−6.59	−6.23	−5.71	−6.79
**(4)** 2-nitrophenol	7.23 ^d^	−3.36	−3.17	−4.07	−2.60	−2.55	−3.44	−3.24	−3.20	−2.37	−2.46
**(5)** 4-hydroxy-3-methoxybenzaldehyde	7.396 ^e^	−1.59	−2.12	−1.55		−2.34	−1.72	−2.17	−1.59		−2.55
**(6)** 2,3-dichlorophenol	7.44 ^d^	−1.71	−1.63	−1.57		−1.83	−3.06	−2.77	−2.93		−2.82
**(7)** 3-cyanophenol	8.61 ^d^	−1.04	−1.04	−0.90	−1.06	−1.18	−2.46	−2.44	−2.29	−2.44	−2.64
**(8)** 4-trifluoromethylphenol	8.675 ^d^	−1.68	−1.29	−0.89		−1.56	−1.51	−3.06	−3.11		−3.08
**(9)** 2-fluorophenol	8.73 ^d^	−0.79	−1.03	−0.88		−0.91	−1.47	−1.50	−1.46		−1.50
**(10)** 3-hydroxybenzaldehyde	8.98 ^d^	−0.28	−0.48	−0.22	−0.42	−0.65	−1.17	−1.42	−1.11	−1.25	−1.66
**(11)** 3-chlorophenol	9.12 ^d^	−0.94	−0.98	−0.92		−1.05	−1.88	−1.85	−1.82		−2.00
**(12)** 4-bromophenol	9.37 ^d^	−0.67	−0.60	−0.66	−0.71	−0.63	−1.54	−1.41	−1.49	−1.51	−1.53
**(13)** acetaminophen	9.50 ^f^	0.27	−0.01	0.42	0.35	−0.05	0.38	0.92	−0.07		0.28
**(14)** 3-methoxyphenol	9.65 ^d^	0.19	0.36	0.17	0.18	0.45	−0.09	0.14	−0.11	−0.02	0.21
**(15)** 4-(2-aminoethyl)phenol	9.74 ^d^	0.86	0.92	0.97		0.79	0.74	0.77	0.82		0.61
**(16)** phenol	9.99 ^d^	−0.19	−0.36	−0.17	−0.18	−0.45	0.09	−0.14	0.11	0.02	−0.21
**(17)** 3-aminophenol	9.82 ^d^	0.59	0.78	0.49		0.87	0.75	1.02	0.67		1.11
**(18)** 4-methoxyphenol	10.21 ^d^	0.55	1.09	0.89	0.78	1.23	0.29	0.93	0.75	0.99	0.88
**(19)** 4-methylphenol	10.26 ^d^	0.43	0.21	0.16	0.36	−0.10	1.06	1.06	1.06	−0.04	0.62
**(20)** 2-(tertbutyl)phenol	10.62 ^d^	0.74	1.18	1.05		0.89	0.37	0.55	0.67		0.19
**MAE (20 phenols)**		**1.43**	**1.61**	**1.51**	**1.34**	**1.61**	**2.01**	**2.28**	**2.15**	**1.99**	**2.23**
**MAE (exc. NO, NO_2_) ^c^**		**0.78**	**0.88**	**0.74**	**0.50**	**0.94**	**1.16**	**1.38**	**1.25**	**0.90**	**1.37**
**MSE (exc. NO, NO_2_) ^c^**		**−0.33**	**−0.31**	**−0.23**	**−0.09**	**−0.41**	**−0.70**	**−0.71**	**−0.74**	**−0.61**	**−0.88**

^a^ Mean absolute (MAE) and signed errors (MSE); ^b^ The calculated aqueous pK_a_ values are reported in Table S10; ^c^ Calculated excluding the results for the nitrophenols and nitrosophenol (**1**–**4**); ^d^ Ref. [42]; ^e^ Ref. [43]; ^f^ Ref. [44]; ^g^ Values reported for 11 phenols.

**Table 3:** Errors in the calculated aqueous pK_a_ values (displayed in Table S11) after using the corresponding pK_a (exp)_ vs. ${\Delta G}_{aq(BA)}^{{}^{\circ}}$ correlation equation at 298.15 K ^a,b,c,d^.

Solvation Method	SMD					PCM				
Name/Functional	M06-2X	B3LYP	BHandHLYP	PBE0 ^f^	TPSS	M06-2X	B3LYP	BHandHLYP	PBE0 ^f^	TPSS
**(1)** 2,4-dinitrophenol	0.46	0.76	0.59	0.55	0.91	0.72	1.11	0.73	0.75	1.27
**(2)** 4-nitrosophenol	−0.15	−0.49	−0.24		−0.58	−0.39	−0.63	−0.42		−0.76
**(3)** 4-nitrophenol	−0.54	−0.76	−0.63	−0.75	−0.84	−0.78	−0.88	−0.82	−1.01	−0.97
**(4)** 2-nitrophenol	−0.38	−0.12	−0.56	−0.14	0.15	0.11	0.32	0.31	0.46	0.63
**(5)** 4-hydroxy-3-methoxybenzaldehyde	0.39	0.25	0.49		0.15	0.72	0.62	0.83		0.49
**(6)** 2,3-dichlorophenol	0.31	0.44	0.46		0.36	0.14	0.37	0.28		0.36
**(7)** 3-cyanophenol	0.02	0.06	0.11	−0.33	0.01	−0.30	−0.24	−0.18	−0.33	−0.30
**(8)** 4-trifluoromethylphenol	−0.32	−0.09	0.08		−0.19	0.04	−0.51	−0.54		−0.50
**(9)** 2-fluorophenol	0.08	0.00	0.06		0.07	0.03	0.04	0.07		0.06
**(10)** 3-hydroxybenzaldehyde	0.20	0.10	0.22	0.13	0.05	0.00	−0.09	0.05	0.02	−0.15
**(11)** 3-chlorophenol	−0.19	−0.19	−0.17		−0.21	−0.37	−0.33	−0.31		−0.37
**(12)** 4-bromophenol	−0.19	−0.16	−0.20	−0.21	−0.15	−0.38	−0.33	−0.34	−0.32	−0.34
**(13)** acetaminophen	0.20	0.02	0.22	0.27	0.04	0.32	0.47	0.13		0.27
**(14)** 3-methoxyphenol	0.08	0.11	0.03	0.11	0.18	0.05	0.08	0.02	0.21	0.15
**(15)** 4-(2-aminoethyl)phenol	0.35	0.31	0.34		0.29	0.33	0.26	0.33		0.24
**(16)** phenol	−0.19	−0.24	−0.24	−0.15	−0.21	−0.12	−0.18	−0.14	0.03	−0.14
**(17)** 3-aminophenol	0.19	0.20	0.08		0.28	0.29	0.30	0.23		0.38
**(18)** 4-methoxyphenol	−0.04	0.12	0.04	0.14	0.23	−0.13	0.03	0.02	0.36	0.05
**(19)** 4-methylphenol	−0.12	−0.30	−0.32	−0.09	−0.40	0.15	0.05	0.11	−0.13	−0.08
**(20)** 2-(tertbutyl)phenol	−0.16	−0.07	−0.11		−0.15	−0.34	−0.37	−0.26		−0.46
**MAE (20-set)**	**0.22**	**0.24**	**0.25**	**0.22**	**0.27**	**0.28**	**0.34**	**0.30**	**0.36**	**0.40**
Correlations from Ref. [38] (20-set) ^e^	0.22	0.26	0.27	0.46	0.21					

^a^ Mean absolute (MAE) and signed errors (MSE); ^b^ ${\Delta G}_{aq(BA)}^{{}^{\circ}}={\Delta G}_{aq\left(A^{-}\right)}^{{}^{\circ}}-{\Delta G}_{aq(HA)}^{{}^{\circ}}$; ^c^ Details of the correlation equations are displayed in Table 4; ^d^ The calculated aqueous pK_a_ values are reported in Table S11; ^e^ Using the pK_a (exp)_ vs. ${\Delta G}_{aq(BA)}^{{}^{\circ}}$ correlation equations reported; ^f^ Values reported for 11 phenols.

**Table 6:** Predicted aqueous pK_a_ values for other phenols at 298.15 K with the M06-2X, B3LYP, BHandHLYP, and TPSS functionals combined with the PCM solvation method using the corresponding pK_a (exp)_ vs. ${\Delta G}_{aq(BA)}^{{}^{\circ}}$ correlation equation for 27-phenols listed in Table 4.

Name/Functional	M06-2X	B3LYP	BHandHLYP	TPSS	Range (Spread)	Average
**(25)** ketobemidone	9.96	10.86	10.97	10.85	9.96–10.97 (1.01)	10.66
**(26)** profadol	10.22	10.24	10.27	10.26	10.22–10.27 (0.05)	10.25
**Antioxidants**						
**(31)** 2-butylated hydroxyanisole	10.75	10.64	10.72	10.66	10.64–10.75 (0.11)	10.69
**(32)** 3-butylated hydroxyanisole	10.77	10.64	10.70	10.68	10.64–10.77 (0.13)	10.70
**(33)** tocol	10.68	10.62	10.66	10.66	10.62–10.68 (0.06)	10.66
**(34)** δ-tocopherol	10.88	10.85	10.87	10.88	10.85–10.88 (0.03)	10.87
**(35)** β-tocopherol	11.09	11.09	11.16	11.09	11.09–11.16 (0.07)	11.11
**(36)** γ-tocopherol	11.16	11.06	11.14	11.07	11.06–11.16 (0.10)	11.11
**(37)** α-tocopherol	11.32	11.26	11.37	11.27	11.26–11.37 (0.11)	11.31
**(38)** N,N-dimethyl-4-aminophenol	10.57	10.64	10.49	10.76	10.49–10.76 (0.27)	10.62
**(39)** 6-hydroxy-5,7,8-trimethyl-1,2,3,4-tetrahydroquinoline	11.47	11.55	11.50	11.53	11.47–11.55 (0.08)	11.51
**(40)** 9-hydroxyjulolidine	11.11	10.86	11.07	11.12	10.86–11.12 (0.26)	11.04
**(41)** 4-butadienylphenol	9.27	9.17	9.21	8.95	8.95–9.27 (0.32)	9.15
**(42)** 4-hydroxystilbene	9.29	9.42	9.29	9.02	9.02–9.42 (0.40)	9.26
**Cannabinoids**						
**(29)** ∆^9^-tetrahydrocannabinol (Δ^9^-THC) ^b^	10.11	10.18	10.14	10.20	10.11–10.20 (0.09)	10.16
**(30)** cannabidiol (CBD) ^a,c^	9.98	9.93	9.95	9.96	9.93–9.98 (0.05)	9.96
**(43)** ∆^8^-tetrahydrocannabinol (Δ^8^-THC)	10.11	10.14	10.13	10.22	10.11–10.22 (0.11)	10.15
**(44)** iso-tetrahydrocannabinol (iso-THC)	10.31	10.29	10.21	10.41	10.21–10.41 (0.20)	10.31
**(45)** ∆^9^-tetrahydrocannabivarin (THCV)	9.83	10.14	10.69	10.21	9.83–10.69 (0.86)	10.22
**(46)** 3-homotetrahydrocannibinol	9.68	9.94	9.95	9.92	9.68–9.95 (0.27)	9.87
**(47)** nabilone	9.96	10.07	10.05	10.02	9.96–10.07 (0.11)	10.03
**(48)** cannabinol (CBN)	9.01	9.43	9.05	9.53	9.01–9.53 (0.52)	9.26
**(49)** cannabichromene (CBC)	9.17	9.19	9.16	9.32	9.16–9.32 (0.16)	9.21
**(50)** cannabigerol (CBG) ^a^	9.95	9.90	10.05	9.94	9.90–10.05 (0.15)	9.96

^a^ Macroscopic pK_a_ values have been calculated by accounting for the degenerate deprotonation sites; ^b^ pK_a_ = 10.60 (Ref. [71]); ^c^ pK_a_ = 9.7 (Ref. [73], minimum experimental value).

## Data Availability

Data available upon request.

## Supplementary Materials

The following information is available online at https://www.mdpi.com/article/10.3390/antiox12071420/s1, The absolute Gibbs free energies of the different species considered in this study at the various levels of theory (Tables S1–S5); individual pK_a_ values and their errors are displayed in Tables S6 and S7 (Tables S8 and S9) when using Reaction (R1) (Reaction (R2)); individual pK_a_ errors when using Reaction (R3) (Table S10); correlated pK_a_ values (Table S11); individual pK_a_ values in test set predictions (Table S12); predicted pK_a_ values for prediction sets not shown in Table 6 (Table S13); optimized structures at the M06-2X(SMD)/6-311++G(d,p) level of theory.
