# Supplementary material for: Calculating the Aqueous pKa of Phenols: Predictions for Antioxidants and Cannabinoids
Source: Antioxidants (Basel). 2023 Jul 13;12(7):1420. doi: 10.3390/antiox12071420 (PMC10376140; doi:10.3390/antiox12071420)
Supplement: Supplementary file 1 [file antioxidants-12-01420-s001.zip › antioxidants-2451499-supplementary.pdf]

Calculating the aqueous pK_a of phenols: Predictions for antioxidants and cannabinoids

Max Walton-Raaby[1,2], Tyler Floen[1], Guillermo García-Díez[1], and Nelaine Mora-Diez[1*]

[1] Department of Chemistry, Thompson Rivers University, Kamloops, BC V2C 0C8, Canada

[2] Department of Chemistry, University of Waterloo, Waterloo, ON N2L 3G1, Canada

Electronic Supplementary Information

(108 pages)

Contents:

Table S1. Absolute Gibbs free energies of the different species considered in this study calculated at the M06–2X/6–311++G(d,p) level of theory in water at 298.15 K.

Table S2. Absolute Gibbs free energies of the different species considered in this study calculated at the B3LYP/6–311++G(d,p) level of theory in water at 298.15 K.

Table S3. Absolute Gibbs free energies of the different species considered in this study calculated at the BHandHLYP/6–311++G(d,p) level of theory in water at 298.15 K.

Table S4. Absolute Gibbs free energies of the different species considered in this study calculated at the PBE0/6–311++G(d,p) level of theory in water at 298.15 K.

Table S5. Absolute Gibbs free energies of the different species considered in this study calculated at the TPSSTPSS/6–311G(d,p) level of theory in water at 298.15 K.

Table S6. Calculated aqueous pK_a values at 298.15 K and the mean absolute error (MAE) at several levels of theory using Reaction 1 for direct calculations.

Table S7. Calculated pK_a errors and the mean absolute error (MAE) at several levels of theory using Reaction 1 for direct calculations.

Table S8. Calculated aqueous pK_a values at 298.15 K and the mean absolute error (MAE) at several levels of theory using Reaction 2 for direct calculations.

Table S9. Calculated pK_a errors and the mean absolute error (MAE) at several levels of theory using Reaction 2 for direct calculations.

* Corresponding author e-mail: nmora@tru.ca

Table S10. Calculated aqueous pK_a values at 298.15 K and the mean absolute error (MAE) at several levels of theory using Reaction 3 (HRef=phenol) for direct calculations.

Table S11. Calculated aqueous pK_a values at 298.15 K and the mean absolute error (MAE) at several levels of theory after using the corresponding $pK_{a\,(exp)}$ *vs.* $\Delta G^{\circ}_{aq\,(BA)}$ correlation equation.

Table S12. Predicted aqueous pK_a values for the phenols in the test set (**21**–**30**) using the corresponding $pK_{a\,(exp)}$ *vs.* $\Delta G^{\circ}_{aq\,(BA)}$ correlation equation listed in Table 4 (obtained from a training set of 20 phenols) at several levels of theory at 298.15 K.

Table S13. Predicted aqueous pK_a values for molecules **25**, **26**, **29**–**50** at levels of theory not shown in Table 6.

Cartesian coordinates of the optimized acids and conjugate bases studied at the M06–2X(SMD)/ 6–311++G(d,p) level of theory.

Table S1. Absolute Gibbs free energies (G° in au) of the different species considered in this study calculated at the M06–2X/6–311++G(d,p) level of theory in water at 298.15 K.

**Solvent Method**	**SMD**			**PCM**		
**Species**	**HA**	**A^-**	**$A^-(H_2O)$**	**HA**	**A^-**	**$A^-(H_2O)$**
**(1)** 2,4-dinitrophenol	-716.34127	-715.90372	-792.32794	-716.34311	-715.90374	-792.32614
**(2)** 4-nitrosophenol	-436.65558	-436.21057		-436.65073	-436.20520	
**(3)** 4-nitrophenol	-511.85569	-511.40873	-587.83366	-511.85344	-511.40563	-587.82822
**(4)** 2-nitrophenol	-511.85052	-511.40248	-587.82714	-511.85085	-511.39785	-587.82189
**(5)** 4-hydroxy-3-methoxybenzaldehyde	-535.15960	-534.70737		-535.15670	-534.69960	
**(6)** 2,3-dichlorophenol	-1226.57647	-1226.12438		-1226.57482	-1226.12056	
**(7)** 3-cyanophenol	-399.60362	-399.14755	-475.57331	-399.60147	-399.14334	-475.56889
**(8)** 4-trifluoromethylphenol	-644.41405	-643.95923		-644.41197	-643.95163	
**(9)** 2-fluorophenol	-406.60540	-406.14853		-406.60271	-406.14217	
**(10)** 3-hydroxybenzaldehyde	-420.67384	-420.21532	-496.64183	-420.66927	-420.20753	-496.63329
**(11)** 3-chlorophenol	-766.97063	-766.51324		-766.96732	-766.50684	
**(12)** 4-bromophenol	-2880.94417	-2880.48565	-2956.91193	-2880.94031	-2880.47854	-2956.90369
**(13)** acetaminophen	-515.32025	-514.85938	-591.28665	-515.31299	-514.84677	-591.27351
**(14)** 3-methoxyphenol	-421.84403	-421.38302	-497.80982	-421.84015	-421.37461	-497.80060
**(15)** 4-(2-aminoethyl)phenol	-441.25260	-440.78994		-441.24535	-440.77782	
**(15)** 4-(2-aminoethyl)phenol$^+$	-441.69972					
**(16)** phenol	-307.35763	-306.89629	-383.32352	-307.35455	-306.88809	-383.31441
**(17)** 3-aminophenol	-362.70511	-362.24286		-362.69944	-362.23170	
**(17)** 3-aminophenol$^+$	-363.13448					
**(18)** 4-methoxyphenol	-346.63854	-346.17569	-422.60274	-346.63663	-346.16726	-422.59243
**(19)** 4-methylphenol	-421.84041	-421.37739	-497.80418	-421.83586	-421.36828	-497.79514
**(20)** 2-(tertbutyl)phenol	-464.46606	-464.00176		-464.46447	-463.99582	
$OH^-(3H_2O)$ [a]	-305.23665			-305.21521		
$3H_2O$ [a]	-229.27658			-229.26291		
**(21)** 2-bromophenol	-2880.94086	-2880.48449		-2880.94011	-2880.48187	
**(22)** 2-chlorophenol	-766.96795	-766.51298		-766.96637	-766.50746	
**(23)** 4-(methylthio)phenol	-744.82252	-744.36302		-744.82025	-744.35738	

**(24)** 4-aminophenol	-362.70156	-362.23638	-362.69550	-362.22452
**(24)** 4-aminophenol+	-363.13416			
**(25)** ketobemidone	-789.02875	-788.56744	-789.01867	-788.55183
**(25)** ketobemidone+	-789.47983			
**(26)** profadol	-675.71002	-675.24765	-675.70625	-675.23790
**(26)** profadol+	-676.15987			
**(27)** tapentadol	-676.89110	-676.42847	-676.88750	-676.41988
**(27)** tapentadol+	-677.34209			
**(28)** (R)-Trolox	-844.47987	-844.01079	-844.46292	-843.98648
**(29)** Δ⁹-tetrahydrocannabinol (Δ⁹-THC)	-968.19007	-967.72885	-968.18884	-967.72114
**(30)** cannabidiol anion C1′ (anion 2)	-968.17420	-967.71181	-968.17034	-967.70117
**(30)** cannabidiol anion C3′ (anion 1)	-968.17420	-967.71231	-968.17034	-967.70159
**(31)** 2-tert-butyl-4-methoxyphenol	-578.94974	-578.48286	-578.94781	-578.47639
**(32)** 3-tert-butyl-4-methoxyphenol	-578.94860	-578.48340	-578.94721	-578.47563
**(33)** tocol	-577.79209	-577.32746	-577.78791	-577.31685
**(34)** δ-tocopherol	-617.07329	-616.60811	-617.06993	-616.59773
**(35)** β-tocopherol	-656.35341	-655.88700	-656.35021	-655.87680
**(36)** γ-tocopherol	-656.35192	-655.88513	-656.34913	-655.87531
**(37)** α-tocopherol	-695.63050	-695.16339	-695.62799	-695.15320
**(38)** N,N-dimethyl-p-aminophenol	-441.23290	-440.76782	-441.22770	-440.75729
**(39)** 6-hydroxy-5,7,8-trimethyl-1,2,3,4-tetrahydroquinoline	-597.18576	-596.71719	-597.18073	-596.70509
**(40)** 9-hydroxyjulolidine	-596.00086	-595.53438	-595.99503	-595.52150
**(41)** 4-butadienylphenol	-462.071469	-461.612632	-462.06967	-461.60683
**(42)** 4-hydroxystilbene	-615.66222	-615.20169	-615.65803	-615.19510
**(43)** Δ⁸-tetrahydrocannabinol (Δ⁸-THC)	-968.19554	-967.73290	-968.19321	-967.72550
**(44)** iso-tetrahydrocannabinol (iso-THC)	-968.18463	-967.72254	-968.18302	-967.71412
**(45)** Δ⁹-tetrahydrocannabivarin (THCV)	-889.63655	-889.17526	-889.63460	-889.16854
**(46)** 3-homotetrahydrocannibinol	-1007.47165	-1007.01038	-1007.47049	-1007.00530
**(47)** nabilone	-1161.26352	-1160.80174	-1161.26039	-1160.79355
**(48)** cannabinol (CBN)	-965.85158	-965.39495	-965.85009	-965.38877
**(49)** cannabichromene (CBC)	-968.16769	-967.71093	-968.16662	-967.70439
**(50)** cannabigerol anion C1′ (anion 2)	-969.35211	-968.89016	-969.34985	-968.88129

**(50)** cannabigerol anion C3′ (anion 1)	-969.35211	-968.89024	-969.34985	-968.88108

[a] These are not HA values; [+] These are species with the protonated amino group.

Table S2. Absolute Gibbs free energies (G° in au) of the different species considered in this study calculated at the B3LYP/6–311++G(d,p) level of theory in water at 298.15 K.

**Solvent Method**	**SMD**			**PCM**		
**Species**	**HA**	**A^-**	**$A^-(H_2O)$**	**HA**	**A^-**	**$A^-(H_2O)$**
**(1)** 2,4-dinitrophenol	-716.62725	-716.18925	-792.64812	-716.62928	-716.18964	-792.64714
**(2)** 4-nitrosophenol	-436.83813	-436.39520		-436.83290	-436.39028	
**(3)** 4-nitrophenol	-512.06668	-511.62107	-588.08251	-512.06448	-511.61855	-588.07884
**(4)** 2-nitrophenol	-512.06231	-511.61319	-588.07310	-512.06286	-511.60949	-588.06826
**(5)** 4-hydroxy-3-methoxybenzaldehyde	-535.38104	-534.92926		-535.37874	-534.92266	
**(6)** 2,3-dichlorophenol	-1226.75443	-1226.30151		-1226.75337	-1226.29852	
**(7)** 3-cyanophenol	-399.76768	-399.31093	-475.77417	-399.76575	-399.30763	-475.76928
**(8)** 4-trifluoromethylphenol	-644.64666	-644.19031		-644.64548	-644.18856	
**(9)** 2-fluorophenol	-406.76788	-406.31084		-406.76606	-406.30564	
**(10)** 3-hydroxybenzaldehyde	-420.85081	-420.39203	-496.85481	-420.84655	-420.38541	-496.84767
**(11)** 3-chlorophenol	-767.12829	-766.67029		-767.12545	-766.66493	
**(12)** 4-bromophenol	-2881.05018	-2880.59081	-2957.05347	-2881.04666	-2880.58466	-2957.04925
**(13)** acetaminophen	-515.53818	-515.07726	-591.53859	-515.53236	-515.06500	-591.52886
**(14)** 3-methoxyphenol	-422.02400	-421.56193	-498.02496	-422.02069	-421.55469	-498.01719
**(15)** 4-(2-aminoethyl)phenol	-441.45095	-440.98748		-441.44434	-440.97678	
**(16)** phenol	-307.49340	-307.03138	-383.49481	-307.49086	-307.02444	-383.48697
**(17)** 3-aminophenol	-362.86187	-362.39853		-362.85687	-362.38860	
**(18)** 4-methoxyphenol	-346.79550	-346.33243	-422.79559	-346.79446	-346.32514	-422.78799
**(19)** 4-methylphenol	-422.02145	-421.55658	-498.02017	-422.01794	-421.54901	-498.01270
**(20)** 2-(tertbutyl)phenol	-464.67803	-464.21209		-464.67697	-464.20799	
$OH^-(3H_2O)$ [a]	-305.38436			-305.36986		
$3H_2O$ [a]	-229.38818			-229.37576		
**(21)** 2-bromophenol	-2881.04637	-2880.58911		-2881.04598	-2880.58708	
**(22)** 2-chlorophenol	-767.12505	-766.66937		-767.12400	-766.66482	

**(23)** 4-(methylthio)phenol	-745.00849	-744.54830	-745.00675	-744.54386
**(24)** 4-aminophenol	-362.85854	-362.39233	-362.85314	-362.38161
**(25)** ketobemidone	-789.36711	-788.90336	-789.36287	-788.88986
**(26)** profadol	-676.01239	-675.54887	-676.00947	-675.54039
**(27)** tapentadol	-677.20492	-676.74097	-677.20268	-676.73465
**(28)** (R)-Trolox	-844.82100	-844.35242	-844.80887	-844.32976
**(29)** Δ^9-tetrahydrocannabinol (Δ^9-THC)	-968.62019	-968.15638	-968.61997	-968.15126
**(30)** cannabidiol anion C1′ (anion 2)	-968.60956	-968.14501	-968.60749	-968.13820
**(30)** cannabidiol anion C3′ (anion 1)	-968.60956	-968.14465	-968.60749	-968.13845
**(31)** 2-tert-butyl-4-methoxyphenol	-579.20572	-578.73756	-579.20449	-578.73288
**(32)** 3-tert-butyl-4-methoxyphenol	-579.20424	-578.73826	-579.20361	-578.73196
**(33)** tocol	-578.03835	-577.57284	-578.03530	-577.56379
**(34)** δ-tocopherol	-617.33956	-616.87327	-617.33727	-616.86434
**(35)** β-tocopherol	-656.63845	-656.17058	-656.63648	-656.16200
**(36)** γ-tocopherol	-656.63808	-656.16925	-656.63560	-656.16131
**(37)** α-tocopherol	-695.93509	-695.46599	-695.93349	-695.45796
**(38)** N,N-dimethyl-p-aminophenol	-441.42997	-440.96324	-441.42595	-440.95435
**(39)** 6-hydroxy-5,7,8-trimethyl-1,2,3,4-tetrahydroquinoline	-597.44981	-596.97890	-597.44533	-596.96794
**(40)** 9-hydroxyjulolidine	-596.25409	-595.78634	-596.24927	-595.77487
**(41)** 4-butadienylphenol	-462.28147	-461.82250	-462.28000	-461.81848
**(42)** 4-hydroxystilbene	-615.93072	-615.47043	-615.92749	-615.46548
**(43)** Δ^8-tetrahydrocannabinol (Δ^8-THC)	-968.62446	-968.15973	-968.62359	-968.15511
**(44)** iso-tetrahydrocannabinol (iso-THC)	-968.61274	-968.14870	-968.61234	-968.14292
**(45)** Δ^9-tetrahydrocannabivarin (THCV)	-890.02377	-889.56026	-890.02308	-889.55463
**(46)** 3-homotetrahydrocannibinol	-1007.92487	-1007.46107	-1007.92536	-1007.45817
**(47)** nabilone	-1161.77384	-1161.30984	-1161.77091	-1161.30288
**(48)** cannabinol (CBN)	-966.27597	-965.81533	-966.27540	-965.81144
**(49)** cannabichromene (CBC)	-968.61209	-968.15309	-968.61232	-968.14988
**(50)** cannabigerol anion C1′ (anion 2)	-969.81028	-969.34779	-969.80888	-969.34005
**(50)** cannabigerol anion C3′ (anion 1)	-969.81028	-969.34760	-969.80888	-969.33990

[a] These are not HA values.

Table S3. Absolute Gibbs free energies (G° in au) of the different species considered in this study calculated at the BHandHLYP/6–311++G(d,p) level of theory in water at 298.15 K.

**Solvent Method**	**SMD**			**PCM**		
**Species**	**HA**	**A^-**	**$A^-(H_2O)$**	**HA**	**A^-**	**$A^-(H_2O)$**
**(1)** 2,4-dinitrophenol	-716.20270	-715.76102	-792.17651	-716.20380	-715.76029	-792.17333
**(2)** 4-nitrosophenol	-436.57252	-436.12396		-436.56743	-436.11772	
**(3)** 4-nitrophenol	-511.75837	-511.30773	-587.72474	-511.75590	-511.30385	-587.72017
**(4)** 2-nitrophenol	-511.75301	-511.30162	-587.71662	-511.75316	-511.29434	-587.70948
**(5)** 4-hydroxy-3-methoxybenzaldehyde	-535.06272	-534.60549		-535.05970	-534.59703	
**(6)** 2,3-dichlorophenol	-1226.53704	-1226.07976		-1226.53543	-1226.07558	
**(7)** 3-cyanophenol	-399.52327	-399.06201	-475.48042	-399.52109	-399.05730	-475.47468
**(8)** 4-trifluoromethylphenol	-644.32771	-643.86628		-644.32536	-643.86322	
**(9)** 2-fluorophenol	-406.54132	-406.07973		-406.53890	-406.07304	
**(10)** 3-hydroxybenzaldehyde	-420.59818	-420.13463	-496.55273	-420.59324	-420.12607	-496.54411
**(11)** 3-chlorophenol	-766.92338	-766.46105		-766.92023	-766.45431	
**(12)** 4-bromophenol	-2880.81902	-2880.35559	-2956.77379	-2880.81534	-2880.34815	-2956.76606
**(13)** acetaminophen	-515.22530	-514.75923	-591.17749	-515.21769	-514.74714	-591.16430
**(14)** 3-methoxyphenol	-421.76640	-421.30054	-497.71903	-421.76288	-421.29211	-497.71013
**(15)** 4-(2-aminoethyl)phenol	-441.16937	-440.70158		-441.16222	-440.68921	
**(16)** phenol	-307.30117	-306.83495	-383.25348	-307.29828	-306.82652	-383.24449
**(17)** 3-aminophenol	-362.63582	-362.16891		-362.63044	-362.15759	
**(18)** 4-methoxyphenol	-346.57483	-346.10768	-422.52680	-346.57338	-346.09873	-422.51719
**(19)** 4-methylphenol	-421.76351	-421.29487	-497.71338	-421.75959	-421.28572	-497.70429
**(20)** 2-(tertbutyl)phenol	-464.37427	-463.90440		-464.37295	-463.89836	
$OH^-(3H_2O)$ [a]	-305.20053			-305.18356		
$3H_2O$ [a]	-229.25496			-229.24263		
**(21)** 2-bromophenol	-2880.81498	-2880.35358		-2880.81439	-2880.35067	
**(22)** 2-chlorophenol	-766.92007	-766.46032		-766.91870	-766.45453	
**(23)** 4-(methylthio)phenol	-744.76868	-744.30360		-744.76582	-744.29779	
**(24)** 4-aminophenol	-362.63215	-362.16168		-362.62630	-362.14946	
**(25)** ketobemidone	-788.86648	-788.39958		-788.86239	-788.38373	

**(26)** profadol	-675.57133	-675.10386	-675.56805	-675.09374
**(27)** tapentadol	-676.75530	-676.28727	-676.75245	-676.27926
**(28)** (R)-Trolox	-844.30813	-843.83323	-844.29279	-843.80896
**(29)** Δ^9-tetrahydrocannabinol (Δ^9-THC)	-967.99408	-967.52695	-967.99343	-967.51988
**(30)** cannabidiol anion C1′ (anion 2)	-967.97908	-967.51080	-967.97643	-967.50218
**(30)** cannabidiol anion C3′ (anion 1)	-967.97908	-967.51050	-967.97643	-967.50186
**(31)** 2-tert-butyl-4-methoxyphenol	-578.83484	-578.36429	-578.83504	-578.35792
**(32)** 3-tert-butyl-4-methoxyphenol	-578.83527	-578.36527	-578.83419	-578.35722
**(33)** tocol	-577.67851	-577.20884	-577.67480	-577.19804
**(34)** δ-tocopherol	-616.95166	-616.48134	-616.94875	-616.47072
**(35)** β-tocopherol	-656.22238	-655.75068	-656.21996	-655.74013
**(36)** γ-tocopherol	-656.22142	-655.75073	-656.21889	-655.73918
**(37)** α-tocopherol	-695.49044	-695.01743	-695.48854	-695.00740
**(38)** N,N-dimethyl-p-aminophenol	-441.14601	-440.67608	-441.14175	-440.66606
**(39)** 6-hydroxy-5,7,8-trimethyl-1,2,3,4-tetrahydroquinoline	-597.06268	-596.58865	-597.05815	-596.57622
**(40)** 9-hydroxyjulolidine	-595.87716	-595.40544	-595.87174	-595.39243
**(41)** 4-butadienylphenol	-461.98384	-461.52009	-461.98196	-461.51416
**(42)** 4-hydroxystilbene	-615.53886	-615.07383	-615.53523	-615.06695
**(43)** Δ^8-tetrahydrocannabinol (Δ^8-THC)	-967.99981	-967.53003	-967.99727	-967.52376
**(44)** iso-tetrahydrocannabinol (iso-THC)	-967.98671	-967.51871	-967.98518	-967.51117
**(45)** Δ^9-tetrahydrocannabivarin (THCV)	-889.45387	-888.98641	-889.45267	-888.97572
**(46)** 3-homotetrahydrocannibinol	-1007.27006	-1006.80207	-1007.26992	-1006.79758
**(47)** nabilone	-1161.03001	-1160.56209	-1161.02578	-1160.55278
**(48)** cannabinol (CBN)	-965.65736	-965.19267	-965.65434	-965.18752
**(49)** cannabichromene (CBC)	-967.97878	-967.51647	-967.97881	-967.51134
**(50)** cannabigerol anion C1′ (anion 2)	-969.16884	-968.70201	-969.16796	-968.69313
**(50)** cannabigerol anion C3′ (anion 1)	-969.16884	-968.70274	-969.16796	-968.69312

[a] These are not HA values.

Table S4. Absolute Gibbs free energies (G° in au) of the different species considered in this study calculated at the PBE0/6–311++G(d,p) level of theory in water at 298.15 K.

**Solvent Method**	**SMD**			**PCM**		
**Species**	**HA**	**A^-**	**$A^-(H_2O)$**	**HA**	**A^-**	**$A^-(H_2O)$**
**(1)** 2,4-dinitrophenol	-715.77315	-715.33045	-791.69793	-715.77564	-715.33070	-791.69719
**(3)** 4-nitrophenol	-511.44603	-510.99578	-510.99578	-511.44416	-510.99290	-587.36058
**(4)** 2-nitrophenol	-511.44164	-510.98848	-587.35679	-511.44251	-510.98383	-587.35115
**(7)** 3-cyanophenol	-399.26706	-398.80752	-475.17851	-399.26501	-398.80348	-475.17344
**(10)** 3-hydroxybenzaldehyde	-420.33080	-419.86907	-496.23966	-420.32652	-419.86158	-496.23188
**(12)** 4-bromophenol	-2880.23489	-2879.77295	-2956.14457	-2880.23129	-2879.76608	-2956.13639
**(13)** acetaminophen	-514.90449	-514.43995	-590.81114	-514.89842		-590.79857
**(14)** 3-methoxyphenol	-421.50221	-421.03773	-497.40884	-421.49896	-421.02991	-497.40053
**(16)** phenol	-307.10769	-306.64285	-383.01508	-307.10478	-306.63494	-383.00573
**(18)** 4-methoxyphenol	-346.35993	-345.89373	-422.26531	-346.35583	-345.88549	-422.25734
**(19)** 4-methylphenol	-421.49930	-421.03230	-497.40410	-421.49599	-421.02352	-497.39493
**(27)** tapentadol	-676.35816	-675.89170		-676.35491	-675.88363	
**(28)** (R)-Trolox	-843.80360	-843.33028		-843.78816	-843.30682	
$OH^-(3H_2O)$ [a]	-305.01088			-304.99414		
$3H_2O$ [a]	-229.11229			-229.09912		

[a] These are not HA values.

Table S5. Absolute Gibbs free energies (G° in au) of the different species considered in this study calculated at the TPSSTPSS/6–311G(d,p) level of theory in water at 298.15 K.

**Solvent Method**	**SMD**			**PCM**		
**Species**	**HA**	**A^-**	**$A^-(H_2O)$**	**HA**	**A^-**	**$A^-(H_2O)$**
**(1)** 2,4-dinitrophenol	-716.75430	-716.31505	-792.77469	-716.75718	-716.31623	-792.77538
**(2)** 4-nitrosophenol	-436.91077	-436.46783		-436.90558	-436.46333	
**(3)** 4-nitrophenol	-512.15294	-511.70732	-588.16961	-512.15108	-511.70531	-588.16581
**(4)** 2-nitrophenol	-512.15016	-511.69940	-588.16080	-512.15121	-511.69586	-588.15548
**(5)** 4-hydroxy-3-methoxybenzaldehyde	-535.46218	-535.01060		-535.46033	-535.00480	
**(6)** 2,3-dichlorophenol	-1226.81756	-1226.36476		-1226.81725	-1226.36223	
**(7)** 3-cyanophenol	-399.83039	-399.37365	-475.83672	-399.82877	-399.37081	-475.83407
**(8)** 4-trifluoromethylphenol	-644.72074	-644.26467		-644.72023	-644.26309	
**(9)** 2-fluorophenol	-406.82243	-406.36484		-406.82076	-406.36007	
**(10)** 3-hydroxybenzaldehyde	-420.91385	-420.45515	-496.91950	-420.91009	-420.44919	-496.91283
**(11)** 3-chlorophenol	-767.18279	-766.72465		-767.18021	-766.71976	
**(12)** 4-bromophenol	-2880.97876	-2880.51916	-2956.98332	-2880.97551	-2880.51349	-2956.97777
**(13)** acetaminophen	-515.61381	-515.15267	-591.61548	-515.60794	-515.14168	-591.60679
**(14)** 3-methoxyphenol	-422.08726	-421.62471	-498.08952	-422.08439	-421.61797	-498.08216
**(15)** 4-(2-aminoethyl)phenol	-441.51485	-441.05136		-441.50870	-441.04121	
**(16)** phenol	-307.53979	-307.07748	-383.54275	-307.53756	-307.07086	-383.53506
**(17)** 3-aminophenol	-362.91386	-362.45002		-362.90912	-362.44039	
**(18)** 4-methoxyphenol	-346.84785	-346.38516	-422.84918	-346.84687	-346.37824	-422.84321
**(19)** 4-methylphenol	-422.08451	-421.61904	-498.08443	-422.08162	-421.61253	-498.07772
**(20)** 2-(tertbutyl)phenol	-464.74994	-464.28434		-464.74929	-464.28079	
$OH^-(3H_2O)$ [a]	-305.38977			-305.37434		
$3H_2O$ [a]	-229.38937			-229.37862		
**(21)** 2-bromophenol	-2880.97585	-2880.51810		-2880.97572	-2880.51656	
**(22)** 2-chlorophenol	-767.17996	-766.72401		-767.17943	-766.72401	
**(23)** 4-(methylthio)phenol	-745.06852	-744.60612		-745.06939	-744.61005	
**(24)** 4-aminophenol	-362.91052	-362.44426		-362.90557	-362.43394	
**(25)** ketobemidone	-789.49271	-789.02899		-789.48866	-789.01582	

**(26)** profadol	-676.12207	-675.65849	-676.11960	-675.65055
**(27)** tapentadol	-677.31063	-676.84663	-677.30868	-676.84040
**(28)** (R)-Trolox	-844.94665	-844.47691	-844.93496	-844.45595
**(29)** Δ^9-tetrahydrocannabinol (Δ^9-THC)	-968.77469	-968.31119	-968.77524	-968.30656
**(30)** cannabidiol anion C1′ (anion 2)	-968.75933	-968.29419	-968.75801	-968.28789
**(30)** cannabidiol anion C3′ (anion 1)	-968.75933	-968.29512	-968.75801	-968.28895
**(31)** 2-tert-butyl-4-methoxyphenol			-579.29377	-578.82214
**(32)** 3-tert-butyl-4-methoxyphenol			-579.29310	-578.82136
**(33)** tocol			-578.12562	-577.65398
**(34)** δ-tocopherol			-617.43375	-616.96070
**(35)** β-tocopherol			-656.73978	-656.26542
**(36)** γ-tocopherol			-656.73903	-656.26482
**(37)** α-tocopherol			-696.04380	-695.56827
**(38)** N,N-dimethyl-p-aminophenol			-441.49514	-441.02286
**(39)** 6-hydroxy-5,7,8-trimethyl-1,2,3,4-tetrahydroquinoline			-597.54080	-597.06360
**(40)** 9-hydroxyjulolidine			-596.34740	-595.87282
**(41)** 4-butadienylphenol			-462.35405	-461.89339
**(42)** 4-hydroxystilbene			-616.03097	-615.56982
**(43)** Δ^8-tetrahydrocannabinol (Δ^8-THC)			-968.77869	-968.30987
**(44)** iso-tetrahydrocannabinol (iso-THC)			-968.76895	-968.29897
**(45)** Δ^9-tetrahydrocannabivarin (THCV)			-890.16662	-889.69787
**(46)** 3-homotetrahydrocannibinol			-1008.08723	-1007.62033
**(47)** nabilone			-1161.95430	-1161.48680
**(48)** cannabinol (CBN)			-966.43415	-965.96980
**(49)** cannabichromene (CBC)			-968.76591	-968.30286
**(50)** cannabigerol anion C1′ (anion 2)			-969.95558	-969.48664
**(50)** cannabigerol anion C3′ (anion 1)			-969.95558	-969.48632

[a] These are not HA values.

Table S6. Calculated aqueous pK_a values at 298.15 K and the mean absolute error (MAE) at several levels of theory using Reaction 1.

**Solvent Method**			**SMD**					**PCM**		
**Name**	**M06-2X**	**B3LYP**	**BHandHLYP**	**PBE0**	**TPSSTPSS**	**M06-2X**	**B3LYP**	**BHandHLYP**	**PBE0**	**TPSSTPSS**
**(1)** 2,4-dinitrophenol	3.13	3.34	5.03	5.50	3.91	3.97	4.10	5.88	6.53	4.70
**(2)** 4-nitrosophenol	6.56	5.61	8.20		5.61	6.81	5.47	8.72		5.30
**(3)** 4-nitrophenol	7.46	6.84	9.16	8.98	6.85	7.85	6.99	9.80	9.44	6.92
**(4)** 2-nitrophenol	7.96	8.46	9.50	10.32	9.21	10.24	10.41	12.92	12.86	11.32
**(5)** 4-hydroxy-3-methoxybenzaldehyde	9.89	9.68	12.19		9.59	12.13	11.66	14.69		11.40
**(6)** 2,3-dichlorophenol	9.82	10.21	12.21		10.15	10.82	11.09	13.39		11.17
**(7)** 3-cyanophenol	11.66	11.97	14.04	13.25	11.96	12.60	12.60	3.20	14.16	12.52
**(8)** 4-trifluoromethylphenol	11.08	11.78	14.12		11.65	13.62	12.05	14.45		12.15
**(9)** 2-fluorophenol	12.02	12.10	14.19		12.35	13.71	13.66	16.16		13.78
**(10)** 3-hydroxybenzaldehyde	12.78	12.90	15.09	14.26	12.87	14.26	13.99	16.76	15.73	13.88
**(11)** 3-chlorophenol	12.26	12.55	14.54		12.60	13.68	13.70	16.18		13.67
**(12)** 4-bromophenol	12.78	13.17	15.04	14.36	13.28	14.27	14.38	16.77	15.86	14.39
**(13)** acetaminophen	13.86	13.89	16.25	15.55	13.98	16.32	16.85	18.32		16.34
**(14)** 3-methoxyphenol	13.93	14.41	16.16	15.52	14.64	16.01	16.22	18.42	17.63	16.41
**(15)** 4-(2-aminoethyl)phenol	14.68	15.06	17.05		15.07	16.92	16.94	19.45		16.91
**(16)** phenol	14.08	14.39	16.33	15.68	14.52	16.44	16.42	18.87	17.99	16.54
**(17)** 3-aminophenol	14.50	15.00	16.64		15.23	17.02	17.27	19.38		17.48
**(18)** 4-methoxyphenol	14.85	15.70	17.44	16.68	15.97	16.95	17.57	19.84	19.20	17.64
**(19)** 4-methylphenol	14.55	15.08	16.90	16.19	15.11	17.30	17.64	20.20	17.63	17.62
**(20)** 2-(tertbutyl)phenol	15.44	16.20	18.01		16.04	17.44	17.59	20.18		17.37
**MAE (20 phenols)**	**3.09**	**3.43**	**5.24**	**4.66**	**3.48**	**4.76**	**4.77**	**7.05**	**6.15**	**4.84**
**MSE (20 phenols)**	**3.00**	**3.25**	**5.24**	**4.66**	**3.36**	**4.75**	**4.66**	**6.51**	**6.15**	**4.71**

Table S7. Calculated pK_a errors and the mean absolute error (MAE) at several levels of theory using Reaction 1.[a]

**Solvent Model**			**SMD**					**PCM**		
**Name**	**M06-2X**	**B3LYP**	**BHandHLYP**	**PBE0**	**TPSSTPSS**	**M06-2X**	**B3LYP**	**BHandHLYP**	**PBE0**	**TPSSTPSS**
**(1)** 2,4-dinitrophenol	-0.94	-0.73	0.96	1.43	-0.16	-0.10	0.03	1.81	2.46	0.63
**(2)** 4-nitrosophenol	0.23	-0.72	1.87		-0.72	0.48	-0.86	2.39		-1.03
**(3)** 4-nitrophenol	0.31	-0.31	2.01	1.83	-0.30	0.70	-0.16	2.65	2.29	-0.23
**(4)** 2-nitrophenol	0.73	1.23	2.27	3.09	1.98	3.01	3.18	5.69	5.63	4.09
**(5)** 4-hydroxy-3-methoxybenzaldehyde	2.49	2.28	4.79		2.19	4.73	4.26	7.30		4.01
**(6)** 2,3-dichlorophenol	2.13	2.51	4.52		2.45	3.13	3.40	5.70		3.47
**(7)** 3-cyanophenol	3.05	3.36	5.43	4.64	3.35	3.99	3.99	-5.41	5.55	3.91
**(8)** 4-trifluoromethylphenol	2.41	3.11	5.45		2.98	4.94	3.37	5.77		3.47
**(9)** 2-fluorophenol	3.32	3.40	5.48		3.65	5.01	4.95	7.45		5.08
**(10)** 3-hydroxybenzaldehyde	3.80	3.92	6.11	5.28	3.89	5.28	5.01	7.78	6.75	4.90
**(11)** 3-chlorophenol	3.14	3.42	5.41		3.48	4.56	4.58	7.06		4.55
**(12)** 4-bromophenol	3.41	3.80	5.67	4.99	3.91	4.90	5.01	7.40	6.49	5.02
**(13)** acetaminophen	4.36	4.39	6.75	6.05	4.48	6.82	7.35	8.82		6.84
**(14)** 3-methoxyphenol	4.28	4.76	6.51	5.87	4.99	6.36	6.57	8.77	7.98	6.76
**(15)** 4-(2-aminoethyl)phenol	4.94	5.32	7.31		5.33	7.18	7.20	9.71		7.17
**(16)** phenol	4.09	4.40	6.34	5.69	4.53	6.45	6.43	8.88	8.00	6.55
**(17)** 3-aminophenol	4.48	4.98	6.62		5.21	7.00	7.25	9.36		7.46
**(18)** 4-methoxyphenol	4.64	5.49	7.23	6.47	5.76	6.74	7.36	9.63	8.99	7.43
**(19)** 4-methylphenol	4.29	4.82	6.64	5.93	4.85	7.04	7.38	9.94	7.37	7.36
**(20)** 2-(tertbutyl)phenol	4.82	5.58	7.39		5.42	6.82	6.97	9.56		6.75
**MAE (20 phenols)**	**3.09**	**3.43**	**5.24**	**4.66**	**3.48**	**4.76**	**4.77**	**7.05**	**6.15**	**4.84**
**MSE (20 phenols)**	**3.00**	**3.25**	**5.24**	**4.66**	**3.36**	**4.75**	**4.66**	**6.51**	**6.15**	**4.71**

[a] The calculated aqueous pK_a values at 298.15 K are reported in Table S6.

Table S8. Calculated aqueous pK_a values at 298.15 K and the mean absolute error (MAE) at several levels of theory using Reaction 2.

**Solvent Method**	**SMD**					**PCM**				
**Name**	**M06-2X**	**B3LYP**	**BHandHLYP**	**PBE0**	**TPSSTPSS**	**M06-2X**	**B3LYP**	**BHandHLYP**	**PBE0**	**TPSSTPSS**
**(1)** 2,4-dinitrophenol	6.99	7.87	6.24	7.18	10.03	5.09	8.30	6.07	7.02	8.89
**(3)** 4-nitrophenol	11.00	10.20	9.67	9.73	11.75	8.90	9.91	8.50	9.39	10.49
**(4)** 2-nitrophenol	11.62	12.52	10.93	11.62	14.53	10.62	14.03	12.16	12.97	15.30
**(7)** 3-cyanophenol	14.81	14.49	13.91	13.32	16.51	12.28	14.90	13.41	13.06	14.83
**(10)** 3-hydroxybenzaldehyde	15.59	15.64	15.10	14.51	16.82	13.85	16.01	14.66	14.47	16.01
**(12)** 4-bromophenol	15.70	15.97	15.00	14.13	17.32	14.14	15.33	14.73	14.60	16.23
**(13)** acetaminophen	16.32	17.29	16.19	15.53	18.65	15.46	18.13	16.62	16.87	17.79
**(14)** 3-methoxyphenol	16.60	17.04	15.99	15.54	18.38	15.49	18.13	16.33	16.22	18.29
**(16)** phenol	16.56	16.83	16.13	15.18	18.05	15.76	18.31	16.81	16.50	18.42
**(18)** 4-methoxyphenol	17.53	18.06	17.26	16.37	19.45	16.03	18.93	17.50	17.43	19.06
**(19)** 4-methylphenol	17.33	17.44	16.29	16.11	18.81	17.63	19.50	17.91	16.25	18.95
**MAE (11 phenols)**	**5.75**	**5.89**	**5.22**	**4.73**	**7.38**	**4.75**	**6.58**	**5.43**	**5.27**	**6.83**
**MSE (11 phenols)**	**5.54**	**5.84**	**4.87**	**4.56**	**7.38**	**4.19**	**6.58**	**5.06**	**5.06**	**6.83**

Table S9. Calculated pK_a errors and the mean absolute error (MAE) at several levels of theory using Reaction 2.[a]

Solvent Model	SMD					PCM				
**Name**	**M06-2X**	**B3LYP**	**BHandHLYP**	**PBE0**	**TPSSTPSS**	**M06-2X**	**B3LYP**	**BHandHLYP**	**PBE0**	**TPSSTPSS**
**(1)** 2,4-dinitrophenol	2.92	3.80	2.17	3.11	5.96	1.02	4.23	2.00	2.95	4.82
**(3)** 4-nitrophenol	3.85	3.05	2.52	2.58	4.60	1.75	2.76	1.35	2.24	3.34
**(4)** 2-nitrophenol	4.39	5.29	3.70	4.39	7.30	3.39	6.80	4.93	5.74	8.07
**(7)** 3-cyanophenol	6.20	5.88	5.30	4.71	7.90	3.67	6.29	4.80	4.45	6.22
**(10)** 3-hydroxybenzaldehyde	6.61	6.66	6.12	5.53	7.84	4.87	7.03	5.68	5.49	7.03
**(12)** 4-bromophenol	6.33	6.60	5.63	4.76	7.95	4.77	5.96	5.36	5.23	6.86
**(13)** acetaminophen	6.82	7.79	6.69	6.03	9.15	5.96	8.63	7.12	7.37	8.29
**(14)** 3-methoxyphenol	6.95	7.39	6.34	5.89	8.73	5.84	8.48	6.68	6.57	8.64
**(16)** phenol	6.57	6.84	6.14	5.19	8.06	5.77	8.32	6.82	6.51	8.43
**(18)** 4-methoxyphenol	7.32	7.85	7.05	6.16	9.24	5.82	8.72	7.29	7.22	8.85
**(19)** 4-methylphenol	7.07	7.18	6.03	5.85	8.55	7.37	9.24	7.65	5.99	8.69
**MAE (11 phenols)**	**5.75**	**5.89**	**5.22**	**4.73**	**7.38**	**4.75**	**6.58**	**5.43**	**5.27**	**6.83**
**MSE (11 phenols)**	**5.54**	**5.84**	**4.87**	**4.56**	**7.38**	**4.19**	**6.58**	**5.06**	**5.06**	**6.83**

[a] The calculated aqueous pK_a values at 298.15 K are reported in Table S8.

Table S10. Calculated aqueous pK_a values at 298.15 K and the mean absolute error (MAE) at several levels of theory using Reaction 3 (HRef=phenol) for direct calculations.

**Solvent Method**	**SMD**					**PCM**				
**Name**	**M06-2X**	**B3LYP**	**BHandHLYP**	**PBE0**	**TPSSTPSS**	**M06-2X**	**B3LYP**	**BHandHLYP**	**PBE0**	**TPSSTPSS**
**(1)** 2,4-dinitrophenol	-0.95	-1.06	-1.30	-0.19	-0.62	-2.48	-2.33	-3.01	-1.46	-1.85
**(2)** 4-nitrosophenol	2.48	1.21	1.86		1.08	0.36	-0.96	-0.16		-1.26
**(3)** 4-nitrophenol	3.38	2.44	2.82	3.28	2.32	1.41	0.56	0.92	1.44	0.36
**(4)** 2-nitrophenol	3.87	4.06	3.16	4.63	4.68	3.79	3.99	4.03	4.86	4.77
**(5)** 4-hydroxy-3-methoxybenzaldehyde	5.80	5.28	5.85		5.05	5.68	5.23	5.81		4.85
**(6)** 2,3-dichlorophenol	5.73	5.81	5.87		5.61	4.38	4.67	4.51		4.62
**(7)** 3-cyanophenol	7.57	7.57	7.71	7.55	7.43	6.15	6.17	6.32	6.17	5.97
**(8)** 4-trifluoromethylphenol	6.99	7.38	7.78		7.12	7.17	5.62	5.56		5.59
**(9)** 2-fluorophenol	7.94	7.70	7.85		7.82	7.26	7.23	7.27		7.23
**(10)** 3-hydroxybenzaldehyde	8.70	8.50	8.76	8.56	8.33	7.81	7.56	7.87	7.73	7.32
**(11)** 3-chlorophenol	8.18	8.14	8.20		8.07	7.24	7.27	7.30		7.12
**(12)** 4-bromophenol	8.70	8.77	8.71	8.66	8.74	7.83	7.96	7.88	7.86	7.84
**(13)** acetaminophen	9.77	9.49	9.92	9.85	9.45	9.88	10.42	9.43		9.78
**(14)** 3-methoxyphenol	9.84	10.01	9.82	9.83	10.10	9.56	9.79	9.54	9.63	9.86
**(15)** 4-(2-aminoethyl)phenol	10.60	10.66	10.71		10.53	10.48	10.51	10.56		10.35
**(16)** phenol	9.80	9.63	9.82	9.81	9.54	10.08	9.85	10.10	10.01	9.78
**(17)** 3-aminophenol	10.41	10.60	10.31		10.69	10.57	10.84	10.49		10.93
**(18)** 4-methoxyphenol	10.76	11.30	11.10	10.99	11.44	10.50	11.14	10.96	11.20	11.09
**(19)** 4-methylphenol	10.69	10.47	10.42	10.62	10.16	11.32	11.32	11.32	10.22	10.88
**(20)** 2-(tertbutyl)phenol	11.36	11.80	11.67		11.51	10.99	11.17	11.29		10.81
**MAE (20 phenols)**	**1.43**	**1.61**	**1.51**	**1.34**	**1.61**	**2.01**	**2.28**	**2.15**	**1.99**	**2.23**
**MAE (exc. NO, NO_2)** [a]	**0.78**	**0.88**	**0.74**	**0.50**	**0.94**	**1.16**	**1.38**	**1.25**	**0.90**	**1.37**
**MSE (20 phenols)**	**-1.06**	**-1.16**	**-1.09**	**-1.04**	**-1.19**	**-1.64**	**-1.74**	**-1.74**	**-1.79**	**-1.84**

[a] Calculated excluding the results for the nitrophenols and nitrosophenol (**1–4**).

Table S11. Calculated aqueous pK_a values at 298.15 K and the mean absolute error (MAE) at several levels of theory after using the corresponding $pK_{a\,(exp)}$ *vs.* $\Delta G^{\circ}_{aq\,(BA)}$ correlation equation.

**Solvent Method**			**SMD**					**PCM**		
**Name**	**M06-2X**	**B3LYP**	**BHandHLYP**	**PBE0**	**TPSSTPSS**	**M06-2X**	**B3LYP**	**BHandHLYP**	**PBE0**	**TPSSTPSS**
**(1)** 2,4-dinitrophenol	4.53	4.83	4.66	4.62	4.98	4.79	5.18	4.80	4.82	5.34
**(2)** 4-nitrosophenol	6.18	5.84	6.09		5.75	5.94	5.70	5.91		5.57
**(3)** 4-nitrophenol	6.61	6.39	6.52	6.40	6.31	6.37	6.27	6.33	6.14	6.18
**(4)** 2-nitrophenol	6.85	7.11	6.67	7.09	7.38	7.34	7.55	7.54	7.69	7.86
**(5)** 4-hydroxy-3-methoxybenzaldehyde	7.78	7.65	7.89		7.55	8.11	8.02	8.23		7.89
**(6)** 2,3-dichlorophenol	7.75	7.88	7.90		7.80	7.58	7.81	7.72		7.80
**(7)** 3-cyanophenol	8.63	8.67	8.72	8.59	8.62	8.31	8.37	8.43	8.28	8.31
**(8)** 4-trifluoromethylphenol	8.36	8.59	8.76		8.48	8.72	8.17	8.13		8.17
**(9)** 2-fluorophenol	8.81	8.73	8.79		8.80	8.76	8.77	8.80		8.79
**(10)** 3-hydroxybenzaldehyde	9.18	9.08	9.20	9.11	9.03	8.98	8.89	9.03	9.00	8.83
**(11)** 3-chlorophenol	8.93	8.93	8.95		8.91	8.75	8.79	8.81		8.75
**(12)** 4-bromophenol	9.18	9.21	9.17	9.16	9.22	8.99	9.04	9.03	9.05	9.03
**(13)** acetaminophen	9.70	9.52	9.72	9.77	9.54	9.82	9.97	9.63		9.77
**(14)** 3-methoxyphenol	9.73	9.76	9.68	9.76	9.83	9.70	9.73	9.67	9.86	9.80
**(15)** 4-(2-aminoethyl)phenol	10.09	10.05	10.08		10.03	10.07	10.00	10.07		9.98
**(16)** phenol	9.80	9.75	9.75	9.84	9.78	9.87	9.81	9.85	10.02	9.85
**(17)** 3-aminophenol	10.01	10.02	9.90		10.10	10.11	10.12	10.05		10.20
**(18)** 4-methoxyphenol	10.17	10.33	10.25	10.35	10.44	10.08	10.24	10.23	10.57	10.26
**(19)** 4-methylphenol	10.14	9.96	9.94	10.17	9.86	10.41	10.31	10.37	10.13	10.18
**(20)** 2-(tertbutyl)phenol	10.46	10.55	10.51		10.47	10.28	10.25	10.36		10.16
**MAE (20 phenols)**	**0.22**	**0.24**	**0.25**	**0.22**	**0.27**	**0.28**	**0.34**	**0.30**	**0.36**	**0.40**

Table S12. Predicted aqueous pK_a values for the phenols in the test set (**21**-**30**) using the corresponding p$K_{a\,(exp)}$ *vs.* $\Delta G^{\circ}_{aq\,(BA)}$ correlation equation listed in Table 4 (obtained from a training set of 20 phenols) at several levels of theory at 298.15 K.

**Solvation method**	**SMD**				**PCM**				**Other Predictions**	
**Functional**	**M06-2X**	**B3LYP**	**BHandHLYP**	**TPSS**	**M06-2X**	**B3LYP**	**BHandHLYP**	**TPSS**	**Ref. 39** [a]	**Ref. 32** [b]
**(21)** 2-bromophenol	8.94	9.01	9.03	8.93	8.33	8.51	8.41	8.53	8.94	
**(22)** 2-chlorophenol	8.42	8.50	8.47	8.45	8.45	8.56	8.49	7.87	8.46	DNC [c]
**(23)** 4-(methylthio)phenol	9.40	9.39	9.52	9.80	9.19	9.19	9.18	8.56	9.34	
**(24)** 4-aminophenol	10.65	10.63	10.63	10.60	10.72	10.69	10.76	10.71	10.47	10.73 [d]
**(25)** ketobemidone	9.79	10.12	9.89	10.08	9.94	10.94	11.08	10.92	9.70	
**(26)** profadol	10.03	10.08	10.01	10.05	10.22	10.27	10.31	10.26	9.91	
**(27)** tapentadol	10.09	10.16	10.13	10.14	10.09	10.08	10.10	10.12	9.97	
**(28)** (R)-Trolox	11.52	11.11	11.55	11.33	11.74	12.00	12.01	12.00	11.25	11.45
**(29)** Δ^9-tetrahydrocannabinol (Δ^9-THC)	9.77	10.14	9.94	10.03	10.10	10.20	10.17	10.19	9.69	9.23
**(30)** cannabidiol (CBD) [c,e]	9.62	9.99	9.88	9.88	10.00	9.96	9.99	9.96	9.52	
**(4)** 2-nitrophenol	6.85	7.11	6.67	7.38	7.34	7.55	7.54	7.86	6.13	3.92

[a] Predicted pK_a values using the correlation equation at the M06-2X(SMD) level of theory; [b] Predicted pK_a values using the three-water clusters for the acid and the conjugate base as done in Ref. 32; [c] Waters would not equilibrate; [d] Value taken from Ref. 32; [e] Macroscopic pK_a values have been calculated by accounting for the degenerate deprotonation sites.

Table S13. Predicted aqueous pKa values for molecules **25**, **26**, **29**-**50** at levels of theory not shown in Table 6.

**Solvation method**			**SMD**		
**Functional**	**M06-2X**	**B3LYP**	**BHandHLYP**	**Spread**	**Average**
**(25)** ketobemidone	9.75	10.09	9.82	0.34	9.89
**(26)** profadol	9.97	10.04	9.93	0.11	9.98
**Antioxidants**					
**(31)** o-butylated hydroxyanisole	10.89	10.94	10.52	0.42	10.78
**(32)** m-butylated hydroxyanisole	10.55	10.52	10.41	0.14	10.49
**(33)** tocol	10.43	10.43	10.35	0.08	10.40
**(34)** delta-tocopherol	10.54	10.58	10.48	0.10	10.53
**(35)** beta-tocopherol	10.79	10.88	10.74	0.14	10.80
**(36)** gamma-tocopherol	10.87	11.07	10.55	0.52	10.83
**(37)** alpha-tocopherol	10.94	11.12	10.99	0.18	11.02
**(38)** N,N-dimethyl-p-aminophenol	10.52	10.66	10.40	0.26	10.53
**(39)** 6-hydroxy-5,7,8-trimethyl-1,2,3,4-tetrahydroquinoline	11.23	11.47	11.18	0.29	11.29
**(40)** 9-hydroxyjulolidine	10.81	10.86	10.74	0.12	10.80
**(41)** 4-butadienylphenol	9.25	9.17	9.22	0.08	9.21
**(42)** 4-hydroxystilbene	9.60	9.42	9.47	0.18	9.50
**Cannabinoids**					
**(29)** Δ^9-tetrahydrocannabinol (Δ^9-THC)	9.74	10.10	9.87	0.36	9.90
**(30)** cannabidiol (CBD) [a]	9.57	9.94	9.79	0.37	9.77
**(43)** Δ^8-tetrahydrocannabinol (Δ^8-THC)	10.03	10.27	10.37	0.34	10.22
**(44)** iso-tetrahydrocannabinol (iso-THC)	9.91	10.14	10.03	0.23	10.03
**(45)** Δ^9-tetrahydrocannabivarin (THCV)	9.75	10.04	9.93	0.29	9.91
**(46)** 3-homotetrahydrocannibinol	9.75	10.10	10.03	0.35	9.96
**(47)** nabilone	9.85	10.14	10.02	0.29	10.00
**(48)** cannabinol (CBN)	8.80	9.49	9.40	0.69	9.23
**(49)** cannabichromene (CBC)	8.83	9.17	8.95	0.34	8.98
**(50)** cannabigerol (CBG) [a]	9.57	9.54	9.37	0.17	9.49

[a] Macroscopic pKa values have been calculated by accounting for the degenerate deprotonation sites.

Cartesian coordinates of the optimized acids and conjugate bases for the phenols with experimental pK_a values studied at the M06–2X(SMD)/6–311++G(d,p) level of theory in water at 298.15 K.

2,4-dinitrophenol

Charge=0, Multiplicity=1

C	1.21275100	1.53999600	-0.00061800
C	1.37493300	0.15137100	-0.00000200
C	0.30360600	-0.71087900	0.00034800
C	-0.97434600	-0.16671300	0.00083300
C	-1.18598100	1.22856000	-0.00009700
C	-0.05672300	2.06357400	-0.00047000
H	2.07717700	2.19044500	-0.00102600
H	0.44098200	-1.78340500	0.00046500
H	-0.22055200	3.13341400	-0.00117200
O	-2.37358900	1.82435100	-0.00022900
H	-3.08154700	1.15368800	-0.00187400
N	2.72285500	-0.40974700	-0.00023700
O	3.66159100	0.36312900	0.00363300
O	2.84526700	-1.61955300	-0.00418200
N	-2.09183200	-1.09558100	0.00078200
O	-3.22722900	-0.63250800	-0.00142100
O	-1.86587200	-2.28445800	0.00217700

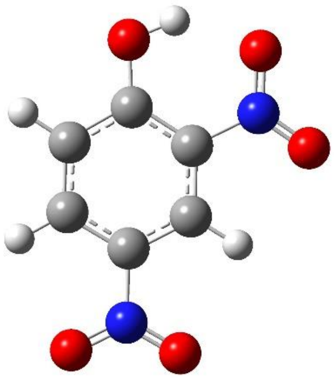

2,4-dinitrophenol anion

Charge=-1, Multiplicity=1

C	-1.21107200	1.54843500	-0.02928500
C	-1.35082700	0.13994800	-0.00019200
C	-0.24470300	-0.68531900	0.00552300
C	1.02099100	-0.12236400	-0.00190600
C	1.25051500	1.31304300	-0.03955300
C	0.03132300	2.09846100	-0.05283800
H	-2.09391100	2.17452700	-0.03314400
H	-0.35767600	-1.76057100	0.03279000
H	0.15741300	3.17421600	-0.08039000
O	2.37056500	1.86437100	-0.08622100
N	-2.65566300	-0.44476600	0.03544100
O	-3.63072300	0.29849400	0.03582700
O	-2.76562500	-1.66576100	0.06245400
N	2.12587600	-1.04501000	0.01340500
O	3.22481400	-0.65550100	0.37676400
O	1.92913400	-2.20572500	-0.33278400

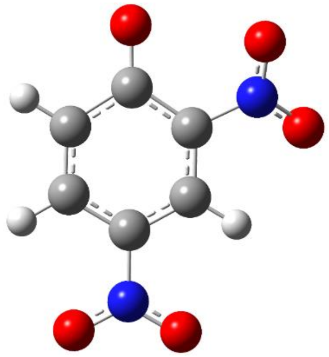

2,4-dinitrophenol anion-H_2O

Charge=-1, Multiplicity=1

C	-1.52680700	-1.58455500	-0.05288200
C	-1.77671300	-0.19570900	0.02319400
C	-0.74561700	0.71938200	-0.04014700
C	0.55220100	0.26061100	-0.19264800
C	0.89337000	-1.14604600	-0.24936000
C	-0.24753200	-2.03046400	-0.18115800
H	-2.35351100	-2.28148200	-0.00580900
H	-0.94285700	1.78181700	0.00736300
H	-0.03567200	-3.09169700	-0.23161000
O	2.05968600	-1.60596400	-0.31966100
N	-3.12258000	0.27907800	0.15410800
O	-4.03014300	-0.54219500	0.20324100
O	-3.32760500	1.48543300	0.21506600
N	1.57724500	1.27193900	-0.28395300
O	2.63080600	1.00034400	-0.83770700
O	1.35185400	2.38050700	0.18561100
O	4.24252600	-0.57603400	1.05248200
H	4.35995400	0.33967400	0.77822500
H	3.47902200	-0.88147100	0.52650000

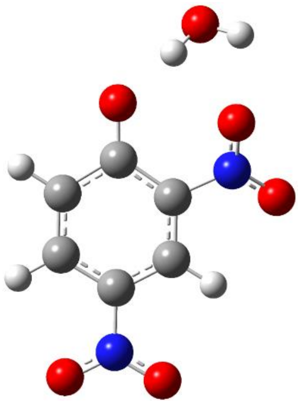

4-nitrosophenol

Charge=0, Multiplicity=1

C	-0.18974600	1.34641600	0.00000200
C	-1.02048600	0.22408000	0.00002400
C	-0.47995100	-1.07110200	0.00003200
C	0.88643900	-1.23203100	0.00001100
C	1.71927500	-0.10019000	-0.00000800
C	1.18432200	1.19019900	0.00000200
H	-0.63625400	2.33434300	-0.00000300
H	-1.13783400	-1.93140100	0.00003800
H	1.33995500	-2.21569200	0.00000600
H	1.84835800	2.04693200	-0.00000300
O	3.05171200	-0.32339700	-0.00001900
H	3.53600900	0.51368300	-0.00003400
N	-2.40662300	0.49581700	0.00002600
O	-3.13958700	-0.47195500	-0.00005200

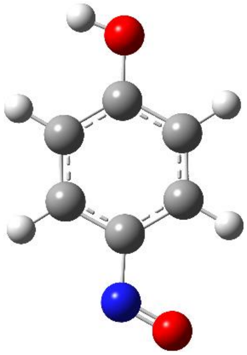

4-nitrosophenol anion

Charge=-1, Multiplicity=1

C	-0.12260500	1.35693100	-0.00000500
C	-0.98789800	0.23073300	0.00000100
C	-0.42122400	-1.07696100	0.00000600
C	0.92912900	-1.24030200	0.00000400
C	1.83532800	-0.10559000	-0.00000200
C	1.23288100	1.20693400	-0.00000600
H	-0.57277700	2.34482900	-0.00000800
H	-1.08143700	-1.93657500	0.00001000
H	1.37330200	-2.22998000	0.00000800
H	1.89451600	2.06595700	-0.00001000
O	3.08587800	-0.26678500	-0.00000200
N	-2.32177200	0.49585400	0.00000200
O	-3.10523700	-0.47642500	0.00000200

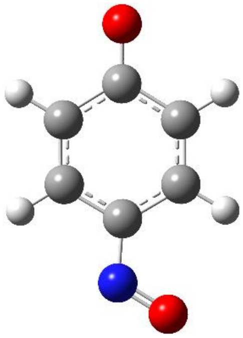

4-nitrophenol

Charge=0, Multiplicity=1

C	-0.01659400	1.22347700	-0.00004700
C	-0.68282800	0.00036100	0.00000200
C	-0.00256300	-1.21280300	0.00003700
C	1.38013700	-1.20217800	0.00004300
C	2.06234200	0.01792500	0.00001100
C	1.36402900	1.22959300	0.00000800
H	-0.57334500	2.15065400	-0.00000500
H	-0.54647400	-2.14762200	-0.00002600
H	1.94055100	-2.12986100	0.00017200
H	1.91717200	2.16022800	-0.00003800
O	3.41367900	0.08527000	-0.00010000
H	3.79774100	-0.80176800	0.00046800
N	-2.13664900	-0.00854800	-0.00002700
O	-2.72607100	1.05908100	0.00010600
O	-2.71338800	-1.08310700	-0.00009400

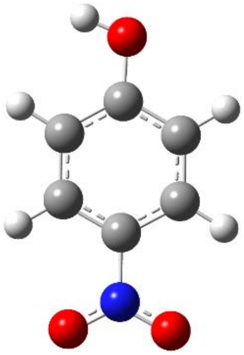

4-nitrophenol anion

Charge=-1, Multiplicity=1

C	-0.04777500	-1.22271200	-0.00005900
C	0.65220900	-0.00006200	-0.00009200
C	-0.04781800	1.22269500	-0.00003000
C	-1.41565700	1.22400800	0.00002700
C	-2.17655500	-0.00013600	0.00001800
C	-1.41571200	-1.22418300	-0.00000300
H	0.50345400	-2.15437700	-0.00006300

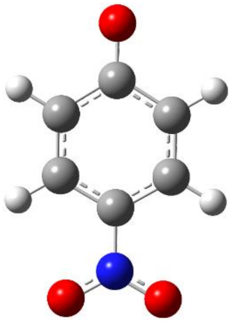

H	0.50340600	2.15436000	-0.00000800
H	-1.96319000	2.15975700	0.00009900
H	-1.96288700	-2.16013000	0.00004500
O	-3.44394900	0.00017400	0.00009200
N	2.06113000	0.00001500	-0.00023500
O	2.67202600	-1.07464800	0.00009800
O	2.67181800	1.07480200	0.00011200

4-nitrophenol anion-H_2O

Charge=-1, Multiplicity=1

C	0.31522700	-1.04095900	-0.01830200
C	1.27124000	-0.01015200	-0.00008200
C	0.87082300	1.33769400	0.00821700
C	-0.46298500	1.64968400	-0.00141000
C	-1.47435000	0.63129800	-0.01990500
C	-1.01907900	-0.73036600	-0.02827900
H	0.63964100	-2.07360300	-0.02474700
H	1.61904600	2.11987100	0.02198300
H	-0.78439400	2.68484600	0.00461700
H	-1.76344500	-1.51836800	-0.04301000
O	-2.71578600	0.92656300	-0.02994500
N	2.64922200	-0.33259700	0.00948400
O	2.99439700	-1.51672100	0.00250100
O	3.48477900	0.57482200	0.02460700
O	-4.53485200	-1.07876200	-0.02754600
H	-3.86828700	-0.35357400	-0.01119200
H	-5.22067300	-0.80141200	0.58758900

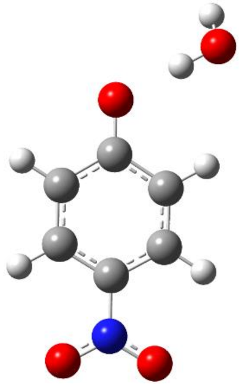

2-nitrophenol

Charge=0, Multiplicity=1

C	-2.54195900	-0.18055500	0.00002000
C	-1.88843200	-1.42008200	0.00011000
C	-0.51223000	-1.45665000	0.00010900
C	0.21539200	-0.26093100	0.00003200
C	-0.42797600	0.99103500	-0.00004100
C	-1.82737400	1.00018500	-0.00005400
H	-3.62480400	-0.14252600	0.00001800
H	-2.45578200	-2.34113500	0.00018100
H	0.02478300	-2.39533500	0.00017700
H	-2.32402900	1.96263000	-0.00011600
O	0.18987700	2.18349700	-0.00010400
H	1.15346200	2.04719400	0.00005600
N	1.65921700	-0.35604600	0.00001500

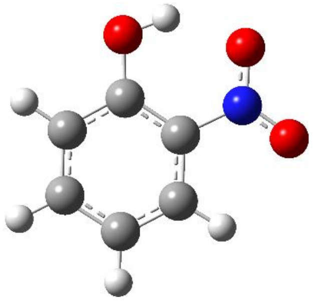

O	2.31718100	0.68334900	0.00011800
O	2.18135900	-1.45141200	-0.00019900

2-nitrophenol anion

Charge=-1, Multiplicity=1

C	-2.52288400	-0.24791000	0.00974900
C	-1.80386200	-1.46978000	0.02717800
C	-0.43712300	-1.42996200	0.02135700
C	0.25539800	-0.19639500	0.00709400
C	-0.43403100	1.07433800	-0.01818600
C	-1.87424900	0.95347600	-0.01305700
H	-3.60770600	-0.26790800	0.01563700
H	-2.32668800	-2.41689700	0.04883500
H	0.14032200	-2.34425700	0.03837100
H	-2.42865900	1.88550500	-0.02841700
O	0.10578300	2.21630400	-0.05418500
N	1.67282000	-0.26384500	0.00580300
O	2.34480700	0.76081200	0.11930900
O	2.22609700	-1.36613100	-0.10510600

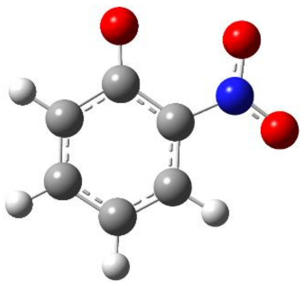

2-nitrophenol anion-H_2O

Charge=-1, Multiplicity=1

C	2.73836000	-0.73669500	0.16150100
C	2.62261100	0.65708700	0.37188900
C	1.39196500	1.24373700	0.24018900
C	0.26109300	0.47661800	-0.11207600
C	0.32788100	-0.94673800	-0.31600500
C	1.64813300	-1.49705600	-0.16441000
H	3.70813100	-1.21271400	0.26090100
H	3.49064900	1.25144500	0.62494800
H	1.26999800	2.30898800	0.38355300
H	1.74028300	-2.56672200	-0.31694800
O	-0.65583500	-1.70938500	-0.58114900
N	-0.97077300	1.18444300	-0.25082900
O	-1.92890600	0.65134300	-0.80433000
O	-1.05049700	2.33763200	0.17979800
O	-2.86694000	-1.42904600	1.01672400
H	-3.23850500	-0.56333800	0.81609600
H	-2.09798600	-1.49482700	0.41237700

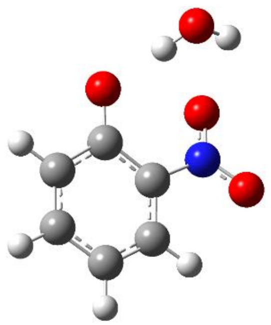

4-hydroxy-3-methoxybenzaldehyde

Charge=0, Multiplicity=1

C	1.41128200	-0.17268800	-0.00011300
C	0.20200000	-0.88415100	-0.00012600
C	-0.99730800	-0.19503200	-0.00007000
C	-0.98812100	1.21220400	0.00000500
C	0.21174900	1.91299200	0.00001900
C	1.41413800	1.22181300	-0.00003700
H	0.22146100	-1.96783000	-0.00018700
H	0.18071900	2.99595600	0.00007900
H	2.35445200	1.75973200	-0.00002500
O	-2.15700200	1.90102800	0.00006200
H	-2.89807000	1.27729000	0.00009400
O	-2.23888300	-0.75291100	-0.00009000
C	2.66374900	-0.93653200	-0.00019300
H	2.54853500	-2.03351200	0.00015000
O	3.77578600	-0.43905200	0.00022500
C	-2.31548900	-2.17727900	0.00013700
H	-1.84146900	-2.58620100	0.89527200
H	-3.37524100	-2.41943500	0.00024400
H	-1.84159000	-2.58648700	-0.89493100

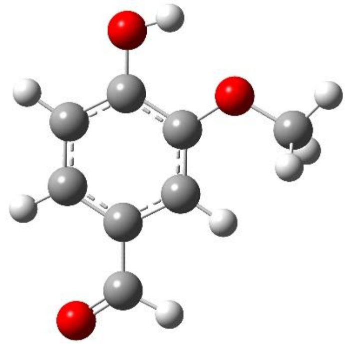

4-hydroxy-3-methoxybenzaldehyde anion

Charge=-1, Multiplicity=1

C	1.38112800	-0.18262800	-0.00002000
C	0.13455000	-0.85565800	0.00000200
C	-1.04215500	-0.14889000	0.00001000
C	-1.05662300	1.29705700	-0.00000600
C	0.22051500	1.93502500	-0.00003400
C	1.39827400	1.22097400	-0.00003900
H	0.13340600	-1.94032200	0.00001500
H	0.22898600	3.01996400	-0.00005000
H	2.35120700	1.73982500	-0.00005800
O	-2.15574800	1.94567400	-0.00001300
O	-2.29247700	-0.71800800	0.00002900
C	2.59221400	-0.96205700	-0.00002500
H	2.44609800	-2.05655700	-0.00000800
O	3.73940700	-0.51148300	-0.00004600
C	-2.35069300	-2.13678900	0.00010600
H	-1.87259600	-2.54862900	0.89333100
H	-3.40717500	-2.39722600	0.00014900
H	-1.87264700	-2.54872700	-0.89310000

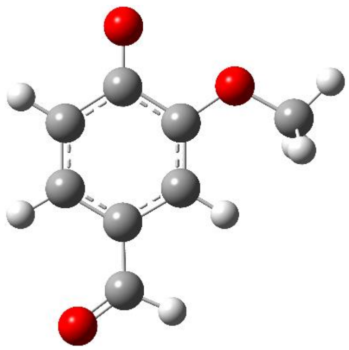

2,3-dichlorophenol

Charge=0, Multiplicity=1

C	-0.36186100	1.95730000	0.00000100
C	-0.75864200	0.62869300	0.00000000
C	0.18271100	-0.39987600	0.00000000
C	1.54625100	-0.08898800	-0.00000100
C	1.94599400	1.24450000	0.00000000
C	0.99701700	2.25433300	0.00000000
H	-1.10838500	2.74059900	0.00000100
H	3.00647600	1.46430100	-0.00000100
H	1.31575900	3.28913700	0.00000100
O	2.51679900	-1.03913500	-0.00000100
H	2.13796600	-1.92890100	-0.00000100
Cl	-0.29557800	-2.06919700	-0.00000100
Cl	-2.45707000	0.25579500	0.00000100

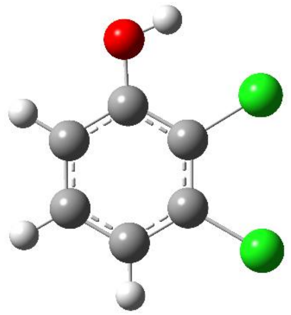

2,3-dichlorophenol anion

Charge=-1, Multiplicity=1

C	-0.17235400	1.96624600	0.00000100
C	-0.66252500	0.66626500	0.00000000
C	0.18425200	-0.43738600	0.00000000
C	1.60971900	-0.29278800	-0.00000100
C	2.07175700	1.05480900	0.00000000
C	1.21205400	2.13772000	0.00000000
H	-0.85338500	2.80630700	0.00000100
H	3.14598100	1.20637700	-0.00000100
H	1.61819900	3.14332700	0.00000100
O	2.41308700	-1.29620000	-0.00000100
Cl	-0.46232000	-2.05753600	0.00000000
Cl	-2.40079300	0.44838300	0.00000100

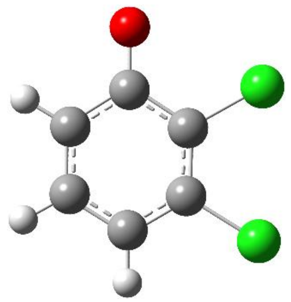

3-cyanophenol

Charge=0, Multiplicity=1

C	-0.68742600	1.79909700	-0.00005000
C	0.64517300	1.40878300	0.00009100
C	0.93813600	0.04261000	0.00016400
C	-0.06673600	-0.92479400	0.00012700
C	-1.39275200	-0.50722600	0.00000800
C	-1.70483500	0.85150400	-0.00007900
H	-0.94006400	2.85220600	-0.00013800
H	1.44594300	2.13680100	0.00016500
H	0.17302900	-1.98088600	0.00018600
H	-2.74593600	1.15659100	-0.00021800

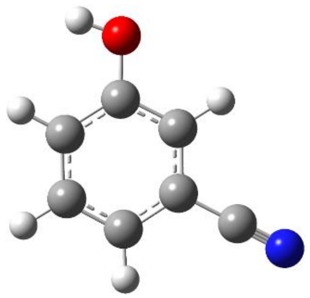

O	-2.35386000	-1.47731700	-0.00008900
H	-3.23285000	-1.07583000	0.00030900
C	2.30932200	-0.38036100	-0.00001400
N	3.41221100	-0.71543100	-0.00015300

3-cyanophenol anion

Charge=-1, Multiplicity=1

C	-0.77899800	1.75526600	-0.00004900
C	0.57199700	1.41424000	-0.00001000
C	0.88944800	0.05165100	0.00002100
C	-0.08627900	-0.94387100	0.00001400
C	-1.46796900	-0.61060600	-0.00002500
C	-1.76801200	0.78254900	-0.00005700
H	-1.06251400	2.80225000	-0.00007500
H	1.35270000	2.16333300	-0.00000400
H	0.20413100	-1.98869700	0.00003900
H	-2.81380800	1.07354700	-0.00008800
O	-2.39201000	-1.51806400	-0.00003300
C	2.27086800	-0.33897800	0.00006100
N	3.38132000	-0.65249000	0.00009400

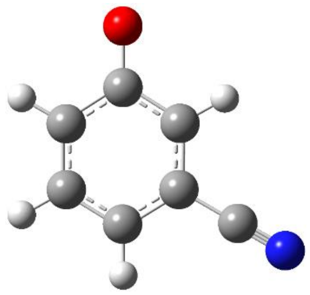

3-cyanophenol anion-H_2O

Charge=-1, Multiplicity=1

C	-0.07638700	1.86255600	0.00000200
C	-1.38794100	1.39507500	-0.00000100
C	-1.57742200	0.00921300	-0.00000100
C	-0.51137600	-0.88883600	0.00000100
C	0.82669400	-0.42298900	0.00000500
C	1.00102000	0.98729000	0.00000500
H	0.10734000	2.93137200	0.00000300
H	-2.23509200	2.06805900	-0.00000200
H	-0.69889700	-1.95669600	0.00000100
H	2.01377200	1.37797800	0.00000800
O	1.83178800	-1.25206900	0.00001100
C	-2.91585200	-0.50993400	-0.00000400
N	-3.99217700	-0.92529100	-0.00000500
O	4.29441000	-0.31806800	-0.00001000
H	3.34382900	-0.61979400	-0.00001000
H	4.25229200	0.64295700	-0.00001700

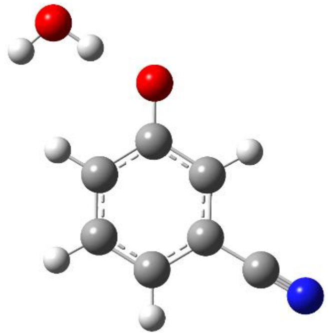

4-trifluoromethylphenol

Charge=0, Multiplicity=1

C	-0.34620700	1.22085600	0.00003700
C	-1.73509700	1.19956500	0.00000400
C	-1.69416800	-1.22409400	0.00001000
C	-0.31064000	-1.19715100	0.00003000
C	0.36254500	0.02521200	0.00005800
H	0.17602600	2.16951900	0.00005200
H	-2.30376100	2.12278500	-0.00001200
H	-2.23596800	-2.16196500	0.00000800
H	0.24815000	-2.12668900	0.00004600
C	1.85502500	0.01369600	0.00000400
F	2.38806000	1.24450400	0.00082400
F	2.36299300	-0.62595900	1.07429000
F	2.36294500	-0.62448300	-1.07519000
C	-2.40463600	-0.02212400	-0.00000500
O	-3.76498500	-0.10346300	-0.00002600
H	-4.15147500	0.78174700	-0.00004000

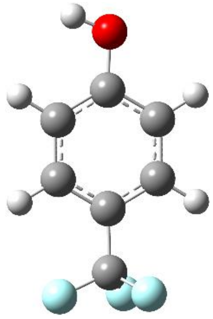

4-trifluoromethylphenol anion

Charge=-1, Multiplicity=1

C	0.39713000	1.22518000	-0.00002700
C	1.78083700	1.21136300	0.00003400
C	1.75097100	-1.20975300	-0.00007300
C	0.37131500	-1.18858800	-0.00012900
C	-0.32178300	0.02834400	-0.00012700
H	-0.12984500	2.17288600	-0.00000400
H	2.33190200	2.14603900	0.00009900
H	2.28112700	-2.15644700	-0.00010000
H	-0.18313400	-2.12255700	-0.00018900
C	-1.80282700	0.00978300	-0.00000900
F	-2.34850500	1.23873000	-0.00107000
F	-2.32633000	-0.63446000	-1.07287800
F	-2.32623300	-0.63249500	1.07411000
C	2.52326300	-0.00652600	0.00001600
O	3.81451600	-0.02559000	0.00007700

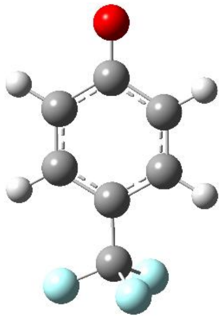

2-fluorophenol

Charge=0, Multiplicity=1

C	1.90315100	-0.64262700	0.00000200
C	0.72025200	-1.37877800	0.00000100
C	-0.48029100	-0.70095900	0.00000000
C	-0.55621100	0.68805800	-0.00000100
C	0.62911900	1.41344700	0.00000000
C	1.85176500	0.74829700	0.00000100
H	2.85527100	-1.15775400	0.00000300
H	0.71617500	-2.46217000	0.00000200
H	0.57554700	2.49577600	-0.00000100
H	2.76821800	1.32565100	0.00000100
O	-1.75058200	1.34911700	-0.00000200
H	-2.48372200	0.71901000	-0.00000300
F	-1.64817200	-1.38645300	0.00000000

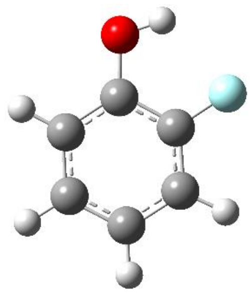

2-fluorophenol anion

Charge=-1, Multiplicity=1

C	1.83718700	-0.73491000	0.00000100
C	0.60080500	-1.38709200	0.00000100
C	-0.55564000	-0.64100400	0.00000000
C	-0.60722500	0.77745300	-0.00000200
C	0.67122700	1.39251100	-0.00000100
C	1.85403700	0.65825500	0.00000100
H	2.75591800	-1.30757600	0.00000300
H	0.52702600	-2.46934500	0.00000200
H	0.70176500	2.47782500	-0.00000100
H	2.80265800	1.18479100	0.00000100
O	-1.73006100	1.42888000	-0.00000200
F	-1.74991300	-1.30089000	0.00000000

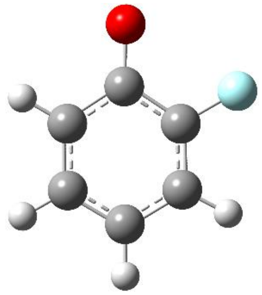

3-hydroxybenzaldehyde

Charge=0, Multiplicity=1

C	0.95397500	1.79644300	0.00004600
C	-0.41914300	1.57217900	0.00016400
C	-0.89509900	0.26124800	-0.00009600
C	-0.01781100	-0.82424700	-0.00014700
C	1.34854900	-0.58572100	0.00011200
C	1.83728200	0.72416600	-0.00012100
H	1.34263300	2.80747300	0.00003500
H	-1.11895900	2.40045500	0.00035200
H	-0.39353800	-1.84101600	-0.00033300
H	2.90993000	0.88911600	-0.00033400

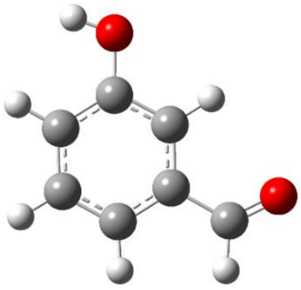

C	-2.35470700	0.04327100	-0.00017600
H	-2.96796300	0.95902200	-0.00040200
O	-2.88270100	-1.05097500	0.00016600
O	2.18343300	-1.66927100	0.00009200
H	3.10377300	-1.37711300	-0.00007300

3-hydroxybenzaldehyde anion

Charge=-1, Multiplicity=1

C	-1.04466200	1.73631000	-0.00006300
C	0.33956800	1.56408900	-0.00009600
C	0.84239900	0.25991200	-0.00004900
C	-0.00202600	-0.85119900	-0.00000100
C	-1.41169000	-0.70482200	-0.00001200
C	-1.89338500	0.63998700	-0.00001400
H	-1.46556100	2.73618000	-0.00008200
H	1.01607500	2.41126800	-0.00013600
H	0.42296600	-1.85012200	0.00003900
H	-2.96919500	0.78810000	0.00001700
C	2.30495900	0.09524200	-0.00007800
H	2.88361600	1.03458800	0.00006700
O	2.88610600	-0.97553600	0.00013700
O	-2.22346700	-1.71910400	0.00010900

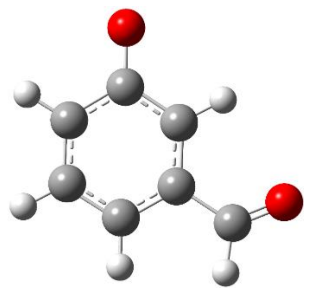

3-hydroxybenzaldehyde anion-H_2O

Charge=-1, Multiplicity=1

C	1.14040200	-2.15154000	-0.15019000
C	1.91207200	-0.98987400	-0.15241300
C	1.26223600	0.23736500	-0.00192500
C	-0.12296700	0.31680400	0.14923700
C	-0.92412700	-0.84728000	0.15200600
C	-0.23704200	-2.08430200	-0.00366700
H	1.61794400	-3.11862000	-0.26470600
H	2.98953500	-1.02671400	-0.26715500
H	-0.59512000	1.28654200	0.27135300
H	-0.82933800	-2.99406000	-0.00377000
C	2.08926500	1.45542500	-0.00402400
H	3.17329400	1.28830700	-0.12298300
O	1.65799600	2.58839900	0.11257400
O	-2.22238800	-0.80410600	0.29107200
O	-3.36418700	1.48142500	-0.32607700
H	-2.74723000	2.18363700	-0.09852400
H	-2.89948500	0.63557400	-0.06891100

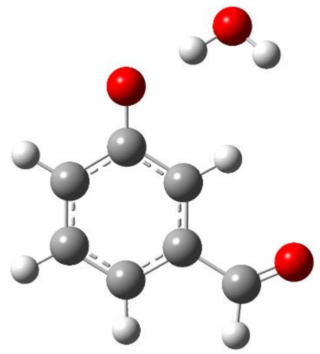

3-chlorophenol

Charge=0, Multiplicity=1

C	-0.56400100	1.41704800	-0.00000800
C	-0.82612800	0.05470000	-0.00001100
C	0.18236300	-0.89840200	0.00000500
C	1.50541900	-0.45997500	-0.00000600
C	1.80375500	0.90107100	-0.00000300
C	0.76772700	1.82554300	0.00000700
H	-1.37338300	2.13513200	-0.00000400
H	-0.04578600	-1.95803100	0.00001700
H	2.83961300	1.21718900	0.00000700
H	0.99751900	2.88440200	0.00000800
O	2.55524900	-1.33549600	0.00001200
H	2.23566000	-2.24689900	-0.00008000
Cl	-2.48884800	-0.49339600	0.00000300

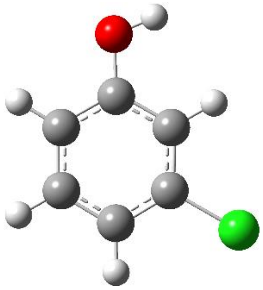

3-chlorophenol anion

Charge=-1, Multiplicity=1

C	-0.49140400	1.41465100	-0.00000200
C	-0.77936700	0.05540500	-0.00000100
C	0.19227100	-0.92880800	0.00000300
C	1.57422000	-0.57967600	0.00001000
C	1.86534200	0.81516300	0.00000300
C	0.86056000	1.76931600	-0.00000200
H	-1.27845800	2.15669500	-0.00000600
H	-0.08508600	-1.97673000	0.00000200
H	2.90721700	1.11876500	0.00000100
H	1.12503800	2.82166000	-0.00000400
O	2.50307400	-1.48410500	0.00000400
Cl	-2.47194300	-0.44257900	-0.00000500

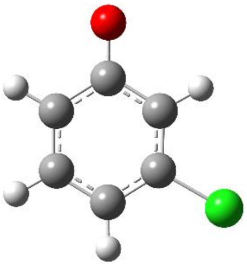

4-bromophenol

Charge=0, Multiplicity=1

C	0.36657500	1.21648500	-0.00000300
C	-0.30924700	0.00253000	-0.00000600
C	0.37848500	-1.20368000	0.00001000
C	1.76902800	-1.19464500	-0.00000600
C	2.45424400	0.01776200	-0.00000700
C	1.75589600	1.22243500	0.00000500
H	-0.17725800	2.15265700	0.00001000
H	-0.15488100	-2.14581700	0.00001400
H	2.32336700	-2.12681800	-0.00001900
H	2.30276500	2.15759600	0.00000900

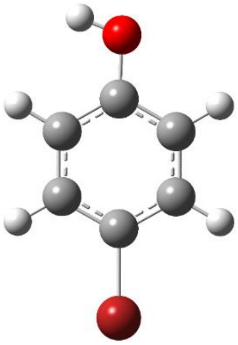

O	3.82132100	0.08125100	-0.00000100
H	4.19214900	-0.81031200	0.00003900
Br	-2.21561700	-0.00693200	0.00000000

4-bromophenol anion

Charge=-1, Multiplicity=1

C	-0.42681100	-1.20664100	0.00000200
C	0.26439700	0.00039200	0.00000100
C	-0.42675000	1.20699000	-0.00000300
C	-1.81530000	1.20446200	-0.00000500
C	-2.57300000	-0.00030400	-0.00000200
C	-1.81500100	-1.20491400	0.00000000
H	0.11360300	-2.14630500	0.00000300
H	0.11301200	2.14705600	-0.00000500
H	-2.35038700	2.14881900	-0.00000900
H	-2.35017600	-2.14932300	0.00000000
O	-3.87382200	0.00014100	-0.00000800
Br	2.17769500	-0.00003700	0.00000400

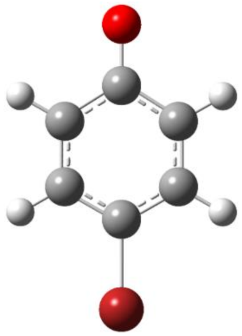

4-bromophenol anion-H_2O

Charge=-1, Multiplicity=1

C	-0.35947500	1.39050200	0.12502800
C	-0.82234700	0.08803100	-0.02727300
C	0.06867500	-0.95578000	-0.24837500
C	1.43095400	-0.69427400	-0.31755900
C	1.95094600	0.61737500	-0.16835300
C	1.00311600	1.64773600	0.05700200
H	-1.05516900	2.20386000	0.29661500
H	-0.29369600	-1.97018800	-0.36943200
H	2.12269200	-1.51111900	-0.49753000
H	1.36397600	2.66404200	0.17658800
O	3.23358300	0.86679800	-0.23714800
Br	-2.69863200	-0.26888400	0.06004600
O	4.78061000	-1.14239300	0.43339900
H	4.17403900	-0.39843200	0.15391200
H	4.39551700	-1.93400100	0.04542000

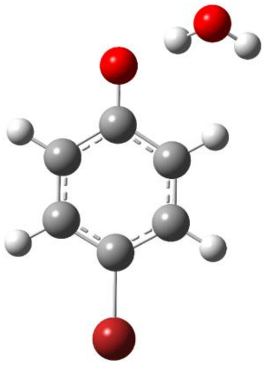

acetaminophen

Charge=0, Multiplicity=1

C	2.57768100	-0.07462300	0.03093200
C	1.73255700	-1.14265900	-0.25417400
C	0.36148800	-0.93746400	-0.36584000
C	-0.17091900	0.33604300	-0.17323800
C	0.68433400	1.40249300	0.10710600
C	2.05355100	1.20344700	0.20430900
H	2.14945100	-2.13340100	-0.40030400
H	-0.28558700	-1.76796000	-0.61113800
H	0.27053200	2.39519600	0.24712000
H	2.72013000	2.02998300	0.41980900
O	3.93737000	-0.22467200	0.14732700
H	4.17997200	-1.14628500	-0.00726100
N	-1.55764600	0.60518100	-0.29952800
H	-1.80331900	1.54462300	-0.58514600
C	-2.57295700	-0.19417700	0.10904400
O	-2.39277300	-1.30808800	0.60296300
C	-3.95803600	0.35716700	-0.08758800
H	-4.49331700	0.28843000	0.86024600
H	-3.96179100	1.38774300	-0.43914000
H	-4.47552300	-0.27387800	-0.81312700

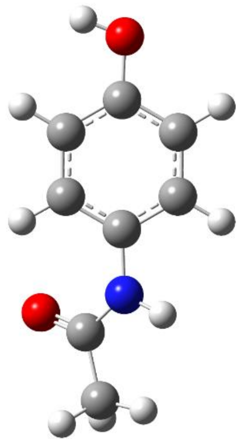

acetaminophen anion

Charge=-1, Multiplicity=1

C	2.68351000	-0.10469400	0.04828500
C	1.79120500	-1.14801900	-0.32509700
C	0.42776400	-0.93710400	-0.47058400
C	-0.12485800	0.32170800	-0.23265200
C	0.72083300	1.37001100	0.12855400
C	2.08773900	1.16926600	0.25803600
H	2.20495600	-2.13493400	-0.50729300
H	-0.21632900	-1.75512900	-0.77336600
H	0.29800100	2.35445200	0.30592500
H	2.73100700	1.99825000	0.53595600
O	3.96358600	-0.30340100	0.18690300
N	-1.52127800	0.56957500	-0.39230400
H	-1.78700400	1.42904100	-0.85485900
C	-2.51265600	-0.17026800	0.14444100
O	-2.30488600	-1.18996400	0.81186500
C	-3.91382500	0.30846400	-0.12159800
H	-4.42670300	0.41937600	0.83497300
H	-3.94590000	1.25130800	-0.66576600

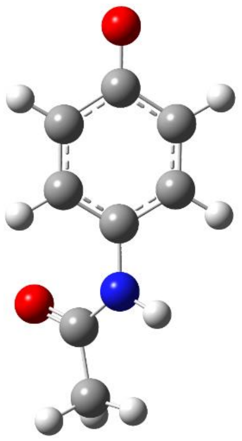

H	-4.43695100	-0.45866400	-0.69589700

acetaminophen anion-H_2O

Charge=-1, Multiplicity=1

C	2.07291400	0.63074600	-0.02820000
C	1.39895800	-0.54156200	-0.45599600
C	0.01518500	-0.59687400	-0.55434400
C	-0.76378100	0.50789300	-0.21232900
C	-0.12897600	1.67797600	0.20339700
C	1.25428900	1.74357600	0.28742500
H	1.98978100	-1.41111900	-0.72626700
H	-0.46463600	-1.50352000	-0.90404000
H	-0.73017200	2.54485200	0.46040800
H	1.73440800	2.66175700	0.60980000
O	3.37970300	0.69039600	0.06789000
N	-2.18547200	0.48738100	-0.32110800
H	-2.63065600	1.32670700	-0.66907200
C	-2.99590500	-0.48642100	0.14352900
O	-2.57293600	-1.51338900	0.68487300
C	-4.47080700	-0.25900000	-0.04448400
H	-4.96161900	-0.36172300	0.92440000
H	-4.69772900	0.71758600	-0.46943100
H	-4.85870700	-1.03921700	-0.70235700
O	4.63557700	-1.60080200	0.16166100
H	3.95894800	-2.27684400	0.26553200
H	4.12867400	-0.73778400	0.10940400

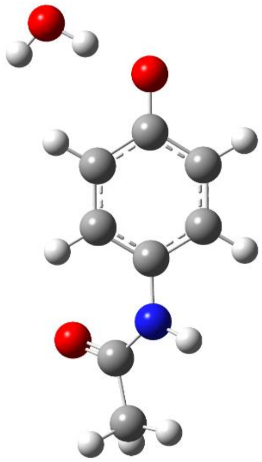

3-methoxyphenol

Charge=0, Multiplicity=1

C	0.55117000	1.79218500	-0.00000400
C	-0.70067000	1.17417400	0.00004200
C	-0.75604800	-0.21870100	-0.00001000
C	0.41604600	-0.97964400	0.00004600
C	1.64427600	-0.33598800	0.00004800
C	1.72631400	1.05783300	-0.00003600
H	0.60150200	2.87485800	-0.00000200
H	-1.59884400	1.77527600	0.00001200
H	0.35621000	-2.06279300	-0.00000600
H	2.69733700	1.53725200	-0.00002100
O	2.82246900	-1.03625300	-0.00002400
H	2.63877200	-1.98389300	-0.00006800
O	-1.91845900	-0.93447500	0.00001200
C	-3.14004100	-0.20178000	-0.00002500

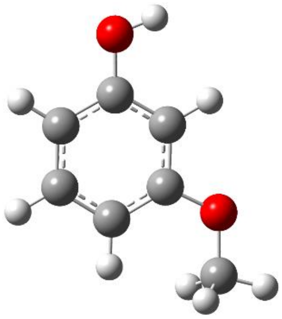

H	-3.21962200	0.42075400	-0.89432400
H	-3.93398300	-0.94515600	0.00009300
H	-3.21973100	0.42105100	0.89404400

3-methoxyphenol anion

Charge=-1, Multiplicity=1

C	-0.63444700	1.75065600	0.00002600
C	0.64133200	1.17319500	0.00002700
C	0.71434500	-0.22113300	-0.00000200
C	-0.43353700	-1.01040800	-0.00001900
C	-1.72937300	-0.43789500	-0.00000200
C	-1.78817300	0.98799700	0.00000800
H	-0.71447500	2.83324100	0.00003900
H	1.52455900	1.79547600	0.00004100
H	-0.32881200	-2.09071700	-0.00004500
H	-2.76390500	1.46282100	0.00000100
O	-2.80853100	-1.16793100	-0.00005300
O	1.90504000	-0.91249200	-0.00000600
C	3.10449300	-0.14934200	0.00002100
H	3.17124600	0.47685600	0.89334000
H	3.92022100	-0.86961300	0.00000900
H	3.17125800	0.47689700	-0.89326800

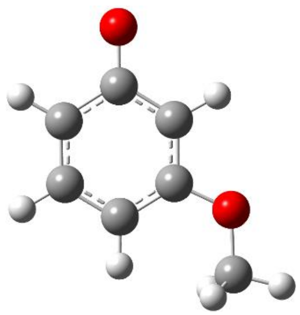

3-methoxyphenol anion-H_2O

Charge=-1, Multiplicity=1

C	-0.76496500	2.10865700	0.16050800
C	-1.62424600	1.00392300	0.12782300
C	-1.04791100	-0.25595200	-0.03859400
C	0.33105600	-0.41213200	-0.16658500
C	1.20366300	0.69818000	-0.13321500
C	0.60672100	1.97770100	0.03638500
H	-1.19417500	3.09724000	0.28810900
H	-2.69186000	1.13789900	0.22695600
H	0.73504900	-1.41017700	-0.30286300
H	1.25175100	2.84944900	0.06475100
O	2.50104200	0.56178200	-0.25509300
O	-1.77875500	-1.41978200	-0.09165000
C	-3.19295500	-1.31006700	0.00774200
H	-3.59699000	-0.70552000	-0.80830000
H	-3.57925400	-2.32464800	-0.06580700
H	-3.48664500	-0.87689700	0.96720200
O	3.48724300	-1.80671500	0.24629700
H	2.74637700	-2.39402600	0.42413100

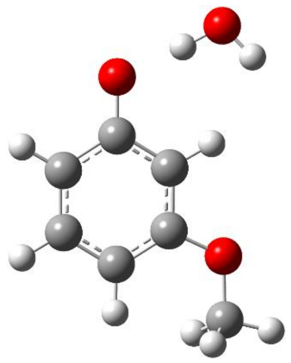

H	3.07132500	-0.91747300	0.04500900

4-(2-aminoethyl)phenol

Charge=0, Multiplicity=1

C	0.31482100	1.20467300	-0.29701800
C	1.67589300	1.22175400	-0.01399900
C	2.36144200	0.01818300	0.12348700
C	1.69065800	-1.19185300	-0.02511100
C	0.32789200	-1.18964800	-0.30777400
C	-0.38282000	0.00338800	-0.45052700
H	-0.21618800	2.14549200	-0.40430700
H	2.21304700	2.15635100	0.09989400
H	2.23634100	-2.12411300	0.07864300
H	-0.19067600	-2.13640000	-0.42305100
O	3.70650400	0.07534800	0.40282200
H	4.05414100	-0.82086800	0.49269300
C	-1.87042400	-0.00414000	-0.68893200
H	-2.15327300	-0.89028800	-1.26549200
H	-2.16218500	0.87752800	-1.26794800
C	-2.65090500	-0.00638600	0.63088400
H	-2.35878200	-0.88191700	1.21623700
H	-2.36862200	0.87409100	1.21363800
N	-4.10739700	-0.01484200	0.47973100
H	-4.38128100	0.79243000	-0.07489800
H	-4.37213000	-0.82703200	-0.07215900

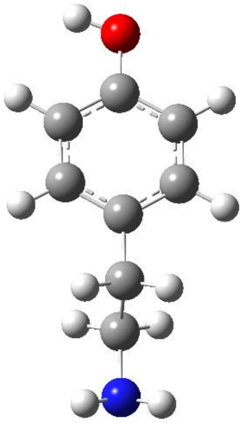

4-(2-aminoethyl)phenol anion

Charge=-1, Multiplicity=1

C	-0.38177900	1.19260800	0.29187800
C	-1.74198800	1.20201400	0.00541400
C	-2.48278300	-0.00000300	-0.15223600
C	-1.74198100	-1.20201500	0.00542300
C	-0.38177200	-1.19259900	0.29188700
C	0.33333700	0.00000700	0.44418700
H	0.14205000	2.13911000	0.40423600
H	-2.26889300	2.14591900	-0.10053600
H	-2.26888100	-2.14592400	-0.10051800
H	0.14206200	-2.13909600	0.40425300
O	-3.76291800	-0.00000800	-0.42064000
C	1.82196200	0.00001100	0.68454600
H	2.11053200	-0.88252500	1.26571700
H	2.11053300	0.88256500	1.26569000
C	2.61831000	-0.00000900	-0.62484200

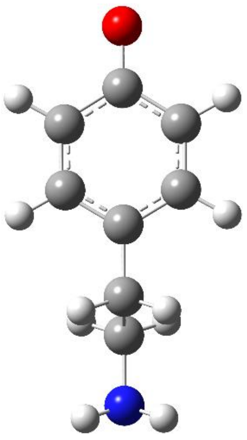

H	2.33817200	-0.87781000	-1.21303000
H	2.33816900	0.87777100	-1.21305900
N	4.07504400	-0.00000400	-0.46005000
H	4.33722200	0.80920800	0.09758500
H	4.33722800	-0.80920800	0.09759400

phenol

Charge=0, Multiplicity=1

C	-1.85157500	0.03361100	0.00000500
C	-1.17499600	-1.18458800	-0.00001300
C	0.21518000	-1.22313200	0.00000400
C	0.93187400	-0.02899400	-0.00001200
C	0.26919400	1.19641600	-0.00001700
C	-1.12246000	1.22008600	0.00001300
H	-2.93443200	0.05748000	0.00002000
H	-1.73147800	-2.11481800	-0.00000700
H	0.75200600	-2.16451300	0.00001400
H	0.84498400	2.11593300	-0.00001200
H	-1.63637600	2.17443800	0.00001700
O	2.30206200	-0.11499100	0.00001600
H	2.68550600	0.77101200	-0.00003000

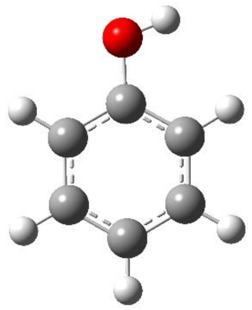

phenol anion

Charge=-1, Multiplicity=1

C	-1.09857800	1.19851600	-0.00000300
C	-1.81532300	0.00000000	0.00000900
C	-1.09858700	-1.19850700	0.00000000
C	0.29053800	-1.20546200	-0.00000900
C	1.04651000	-0.00001000	0.00001000
C	0.29053500	1.20545900	-0.00000700
H	-1.63227800	2.14416100	0.00000300
H	-2.89862600	0.00000800	0.00000300
H	-1.63227800	-2.14415800	0.00000800
H	0.83175600	-2.14711000	-0.00002900
H	0.83178000	2.14709100	-0.00002200
O	2.35113500	0.00000400	0.00000500

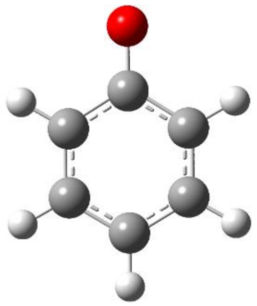

phenol anion-H_2O

Charge=-1, Multiplicity=1

C	-2.20445800	-0.70132400	0.02180900
C	-2.33562500	0.68849900	0.02030700
C	-1.17594700	1.46435100	-0.00341400
C	0.08240000	0.87453300	-0.02499400
C	0.23904500	-0.53545500	-0.02375700
C	-0.95258700	-1.30347200	0.00091100
H	-3.09207800	-1.32600400	0.04013700
H	-3.31386400	1.15345700	0.03720400
H	-1.25216100	2.54724900	-0.00528400
H	0.97392100	1.49400500	-0.04409700
H	-0.86450900	-2.38555600	0.00259500
O	1.42090000	-1.10543100	-0.04438800
O	3.52787400	0.42130300	0.09790400
H	3.34709100	1.20170600	-0.43493700
H	2.69444700	-0.13461800	0.03108000

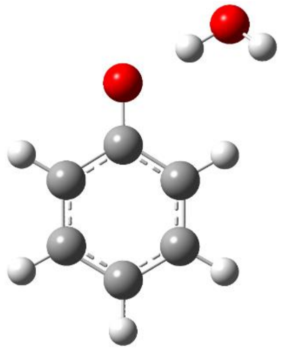

3-aminophenol

Charge=0, Multiplicity=1

C	1.15875400	1.13455000	-0.00373700
C	1.21553800	-0.26717900	-0.00974900
C	0.02515000	-1.00048900	-0.00731700
C	-1.19588500	-0.33663200	0.00000700
C	-1.26517900	1.05497600	0.00482700
C	-0.07292100	1.77489700	0.00410800
H	2.08012600	1.70605700	-0.00348000
H	0.04657400	-2.08503400	-0.00994000
H	-2.22879300	1.55156300	0.01158500
H	-0.10839400	2.85852200	0.01118600
O	-2.32742100	-1.11560700	0.00364700
H	-3.10777200	-0.54740000	0.01083600
N	2.44189400	-0.92322900	-0.07819900
H	2.43177400	-1.86841400	0.28304500
H	3.21984700	-0.38858000	0.28614800

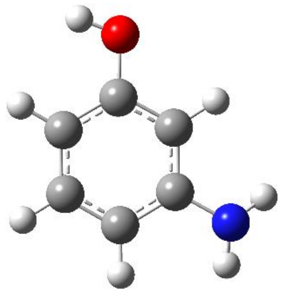

3-aminophenol anion

Charge=-1, Multiplicity=1

C	1.09206600	1.15552600	0.00491100
C	1.17084500	-0.24485400	0.01216900
C	0.00446100	-1.01073300	0.00967000
C	-1.28628100	-0.42065200	-0.00015000
C	-1.33452100	1.00295300	-0.00576800

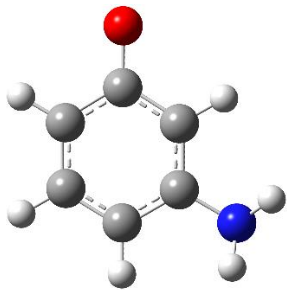

C	-0.16746300	1.75387800	-0.00458800
H	1.99834500	1.75078200	0.00559000
H	0.07953400	-2.09510900	0.01323200
H	-2.30536700	1.48767600	-0.01458100
H	-0.23496400	2.83773000	-0.01298900
O	-2.37337700	-1.14411200	-0.00605600
N	2.42491700	-0.87385100	0.08281000
H	2.42765900	-1.80896200	-0.30533500
H	3.17274600	-0.31896100	-0.31460500

4-methoxyphenol

Charge=0, Multiplicity=1

C	-0.26800900	1.19631800	-0.23012000
C	-0.93184300	-0.02308300	-0.31909900
C	-0.22826900	-1.21597700	-0.21084300
C	1.14995200	-1.19355900	-0.01964000
C	1.81288400	0.02730600	0.06812200
C	1.10767500	1.22372200	-0.03766700
H	-0.83174100	2.11838500	-0.31701800
H	-0.76006000	-2.15799500	-0.28219800
H	1.71140100	-2.11822800	0.05978500
H	1.64227500	2.16389300	0.02916700
O	3.17113100	0.10620400	0.25842900
H	3.54135600	-0.78299800	0.32044400
O	-2.30095200	-0.05030900	-0.54011300
C	-3.05733900	0.02113700	0.67163800
H	-4.10902700	-0.00561900	0.39099600
H	-2.82482700	-0.83196600	1.31514400
H	-2.84111400	0.95218800	1.20280500

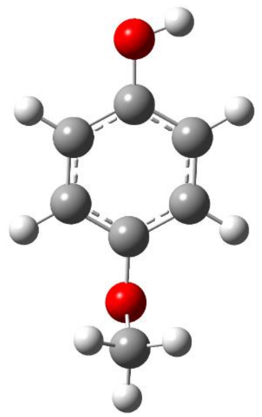

4-methoxyphenol anion

Charge=-1, Multiplicity=1

C	0.19314800	-1.20197300	-0.21700100
C	0.88558800	0.00012700	-0.32006600
C	0.19288900	1.20218700	-0.21675800
C	-1.18296000	1.20432600	-0.01957100
C	-1.93013800	-0.00015800	0.08928200
C	-1.18282000	-1.20438300	-0.01978000
H	0.73990500	-2.13664900	-0.29804900
H	0.73968700	2.13686100	-0.29764900
H	-1.71663400	2.14685900	0.05412300
H	-1.71619000	-2.14710800	0.05373700
O	-3.22361300	-0.00010600	0.27628200

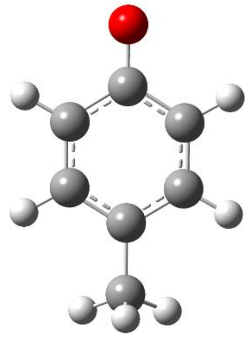

O	2.26256600	0.00044600	-0.54629900
C	3.01987300	-0.00032300	0.66306000
H	4.07310900	0.00045900	0.38381700
H	2.79707400	0.89209300	1.25540300
H	2.79794800	-0.89404800	1.25375300

4-methoxyphenol anion-H_2O

Charge=-1, Multiplicity=1

C	-1.08936200	1.25540200	-0.27786800
C	-1.48264000	-0.07815700	-0.32217900
C	-0.53931800	-1.08115200	-0.12928800
C	0.79277700	-0.75520400	0.09992600
C	1.23125900	0.59168700	0.14918900
C	0.24143300	1.58538300	-0.04988200
H	-1.83239900	2.03249700	-0.42828700
H	-0.85513000	-2.11924500	-0.16232100
H	1.52292700	-1.54461900	0.24838800
H	0.54505600	2.62696500	-0.02292800
O	2.48920000	0.90684200	0.36825300
O	-2.81317800	-0.40874800	-0.57557700
C	-3.59602100	-0.49001500	0.61522700
H	-4.61009700	-0.75322800	0.31621000
H	-3.19852000	-1.26040200	1.28253700
H	-3.60509000	0.47270600	1.13458000
O	4.23161400	-0.94742400	-0.18035000
H	3.53890500	-0.25644100	0.04745700
H	3.88448100	-1.77125700	0.17501900

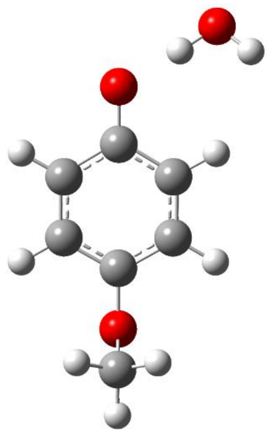

4-methylphenol

Charge=0, Multiplicity=1

C	0.64175600	-1.19870200	-0.00434300
C	1.37568400	-0.01326100	-0.00471400
C	0.66770600	1.19336300	-0.00431400
C	-0.72064500	1.22021000	-0.00070400
C	-1.43044900	0.02145900	0.00157700
C	-0.75203400	-1.19165400	-0.00070500
H	1.16440000	-2.14964100	-0.00781500
H	1.21489700	2.13089300	-0.00776000
H	-1.26310200	2.15870300	-0.00148500
H	-1.31327700	-2.12042500	-0.00150000
C	2.88210000	-0.01985600	0.00548500
H	3.26819700	0.35573400	0.95697700
H	3.26906700	-1.02923900	-0.14063700

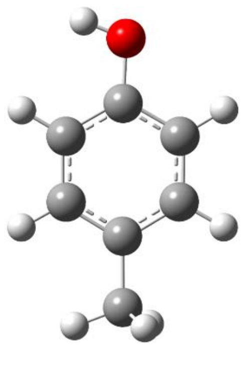

H	3.28264100	0.61977200	-0.78431100
O	-2.80395100	0.09505300	0.00373900
H	-3.17593300	-0.79557900	0.00292800

4-methylphenol anion

Charge=-1, Multiplicity=1

C	-0.60142400	1.19118800	-0.01214900
C	-1.33394000	0.00000400	-0.01288200
C	-0.60142300	-1.19119000	-0.01214800
C	0.78903600	-1.20066700	-0.00285100
C	1.54763100	0.00000000	0.00438300
C	0.78904400	1.20066500	-0.00285200
H	-1.13509200	2.13843900	-0.02004600
H	-1.13508900	-2.13844000	-0.02004900
H	1.32517600	-2.14537000	-0.00584600
H	1.32517300	2.14537200	-0.00584500
C	-2.84205700	0.00000100	0.01680900
H	-3.22523000	-0.00046100	1.04240400
H	-3.24626900	0.88436600	-0.48069600
H	-3.24628800	-0.88391100	-0.48149000
O	2.85705200	-0.00000100	0.01271400

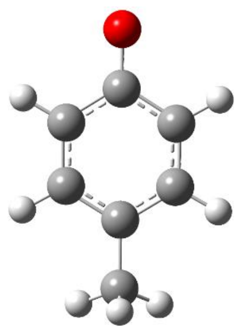

4-methylphenol anion-H_2O

Charge=-1, Multiplicity=1

C	-1.54782900	1.11055400	0.16229400
C	-1.92097000	-0.23038100	0.02854800
C	-0.89920500	-1.15109000	-0.22006000
C	0.43234900	-0.76187100	-0.32374100
C	0.82128300	0.59219400	-0.18019300
C	-0.22161800	1.51694100	0.06466800
H	-2.31631800	1.85666700	0.34717100
H	-1.15233500	-2.20125300	-0.34084000
H	1.19837900	-1.50330100	-0.53139300
H	0.03614500	2.56631600	0.17137900
C	-3.35726300	-0.66724500	0.17287200
H	-3.59400300	-0.93151600	1.20842100
H	-4.04242200	0.12916700	-0.12521600
H	-3.56629400	-1.54451100	-0.44313800
O	2.07837100	0.97460700	-0.27520000
O	3.81051900	-0.84505200	0.40296600
H	3.12280300	-0.16841300	0.12056000
H	3.36244200	-1.69421100	0.34460300

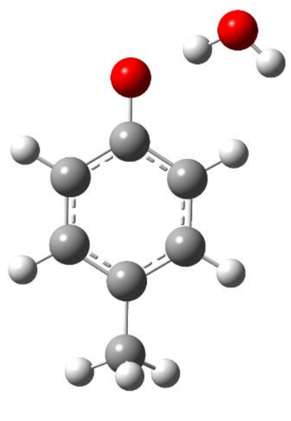

2-(tertbutyl)phenol

Charge=0, Multiplicity=1

C	2.13308200	-1.52334200	-0.00004900
C	0.74113800	-1.45338100	-0.00004300
C	0.04332700	-0.24099100	0.00007900
C	0.82881200	0.92876800	0.00006600
C	2.22122700	0.87230400	0.00015600
C	2.87808400	-0.35189400	0.00008500
H	2.62378800	-2.48923900	-0.00014900
H	0.18655200	-2.38237700	-0.00014900
H	2.78274100	1.80154000	0.00023500
H	3.96140100	-0.38181200	0.00011600
O	0.20625000	2.15108500	-0.00006700
H	0.87523900	2.84702300	-0.00083000
C	-1.48987100	-0.19030200	0.00002400
C	-1.99783000	0.52951900	-1.26162700
H	-3.09174400	0.52946000	-1.26447700
H	-1.65733800	1.56308600	-1.31081600
H	-1.65703800	0.00846400	-2.16085100
C	-2.10147400	-1.59614400	0.00021100
H	-1.81322900	-2.16526900	0.88780500
H	-3.19026600	-1.50359700	0.00018300
H	-1.81318100	-2.16552400	-0.88721700
C	-1.99810900	0.52994500	1.26136200
H	-1.65761600	1.56353200	1.31028500
H	-3.09202800	0.52991700	1.26394600
H	-1.65759000	0.00922500	2.16086900

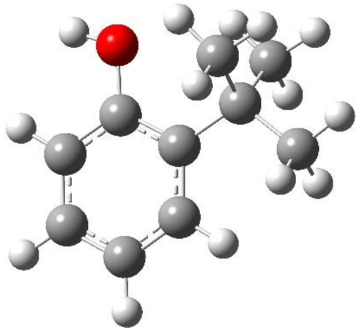

2-(tertbutyl)phenol anion

Charge=-1, Multiplicity=1

C	2.14718500	-1.49268700	-0.00003200
C	0.75236700	-1.40681600	-0.00004800
C	0.05544800	-0.19660600	-0.00001700
C	0.81183100	1.03180700	-0.00001500
C	2.23035300	0.91099000	0.00004800
C	2.88456400	-0.31217300	0.00002900
H	2.63562600	-2.45998800	-0.00005700
H	0.19558600	-2.33638400	-0.00008500
H	2.80179000	1.83490500	0.00009000
H	3.96996500	-0.34194900	0.00006300
O	0.26586000	2.21373400	-0.00006200
C	-1.47935400	-0.17135400	0.00000900
C	-2.00173500	0.53824000	-1.26197800

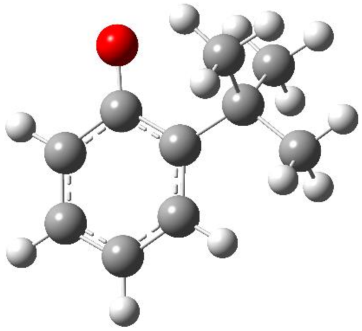

H	-3.09633200	0.55569900	-1.25341700
H	-1.64174000	1.56380600	-1.33137600
H	-1.68117100	-0.00095000	-2.15862200
C	-2.08622100	-1.58051100	-0.00011300
H	-1.79581300	-2.15040800	0.88665100
H	-3.17636500	-1.49544300	-0.00003500
H	-1.79591000	-2.15020300	-0.88704600
C	-2.00167600	0.53800400	1.26216100
H	-1.64125100	1.56339000	1.33204500
H	-3.09626400	0.55597600	1.25337100
H	-1.68157300	-0.00169800	2.15865700

$OH^{-}(3H_2O)$

Charge=-1, Multiplicity=1

O	-0.25123600	-0.20637800	0.69774600
H	-0.26901100	-0.23817600	1.65902700
O	-2.62908900	-0.13608700	-0.30535000
H	-1.70272800	-0.18780000	0.09668600
H	-2.73724900	-0.95392300	-0.79945100
O	1.77810800	-1.52251400	-0.27163200
H	0.99740300	-1.02816700	0.12445000
H	2.39030400	-0.83335700	-0.54706800
O	1.13278900	1.82296800	-0.15999100
H	0.56842200	1.06923700	0.19566000
H	0.50828000	2.50826800	-0.41548700

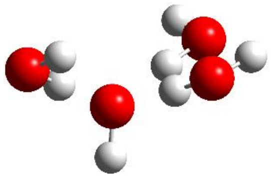

$3H_2O$

Charge=0, Multiplicity=1

O	-1.79998400	-0.76453200	0.03183900
H	-1.28733900	0.06156000	-0.00789200
H	-1.16537500	-1.45019800	-0.20122400
O	-0.08671800	1.49616100	-0.09850200
H	-0.04219300	2.00730500	0.71643700
H	0.65310400	0.86308900	-0.04594900
O	1.65600200	-0.66688000	-0.08231300
H	2.52792200	-0.66314000	0.32831200
H	1.15948400	-1.33659900	0.40212500

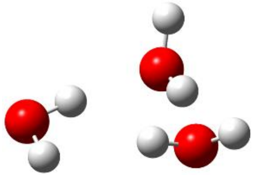

Cartesian coordinates of the optimized acids and conjugate bases of the test set studied at the M06–2X(SMD)/6–311++G(d,p) level of theory in water at 298.15 K.

2-bromophenol

Charge=0, Multiplicity=1

C	0.74291500	-1.45506600	0.00000700
C	0.07891200	-0.23502600	0.00000400
C	0.78886400	0.96785400	0.00002900
C	2.18296000	0.92427100	0.00002500
C	2.84907100	-0.29477600	0.00005200
C	2.13330700	-1.48830500	0.00006400
H	0.17075400	-2.37447100	-0.00004200
H	2.73207800	1.86026500	-0.00001800
H	3.93241200	-0.30789100	0.00009000
H	2.64750700	-2.44108800	0.00008300
O	0.10044300	2.14046600	0.00007400
H	0.72369700	2.87845400	0.00004800
Br	-1.81903300	-0.20722000	-0.00005300

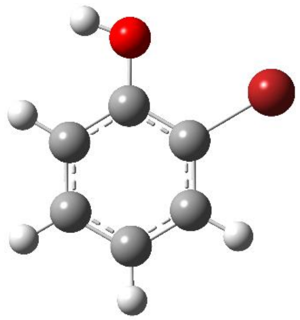

2-bromophenol anion

Charge=-1, Multiplicity=1

C	-0.74686600	-1.41285600	0.00014100
C	-0.09289100	-0.18906000	0.00006000
C	-0.76988600	1.06856400	0.00001800
C	-2.19355800	0.94971800	-0.00015200
C	-2.85261900	-0.26877500	-0.00012000
C	-2.13917000	-1.46931700	0.00007900
H	-0.16470900	-2.32738300	0.00026500
H	-2.75947700	1.87649400	-0.00027900
H	-3.93785000	-0.28455900	-0.00023100
H	-2.64697600	-2.42568000	0.00016900
O	-0.18008400	2.21173700	0.00020800
Br	1.82056100	-0.18864000	-0.00005000

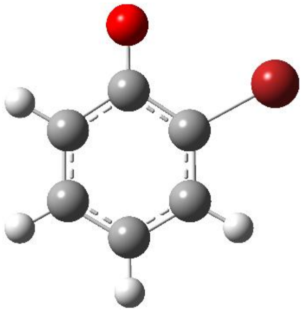

2-chlorophenol

Charge=0, Multiplicity=1

C	-1.78733900	-1.32302400	0.00004000
C	-0.40533000	-1.47465200	0.00003400
C	0.41101900	-0.35155100	0.00000400
C	-0.13183400	0.93379800	-0.00001800
C	-1.51851600	1.07521500	-0.00001400
C	-2.33935100	-0.04481800	0.00001500
H	-2.42402100	-2.19871400	0.00006300

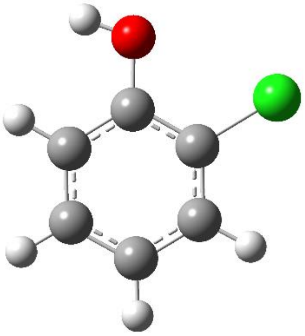

H	0.04635700	-2.45910100	0.00005200
H	-1.93771400	2.07576300	-0.00003400
H	-3.41484000	0.08497900	0.00001900
O	0.71006000	2.00306900	-0.00004500
H	0.19139600	2.81865400	-0.00005300
Cl	2.14626200	-0.54329100	-0.00000300

2-chlorophenol anion

Charge=-1, Multiplicity=1

C	-1.75376800	-1.33537600	0.00000000
C	-0.36475300	-1.43536900	0.00000100
C	0.41801700	-0.29082100	-0.00000100
C	-0.11397200	1.03229200	-0.00000100
C	-1.53894500	1.07644200	0.00000100
C	-2.32862200	-0.06293700	-0.00000100
H	-2.36522700	-2.22888700	0.00000100
H	0.11822100	-2.40627200	-0.00000100
H	-1.99911000	2.05974100	0.00000200
H	-3.40883700	0.04087900	0.00000000
O	0.61239900	2.10044900	0.00000000
Cl	2.16753100	-0.48085000	0.00000000

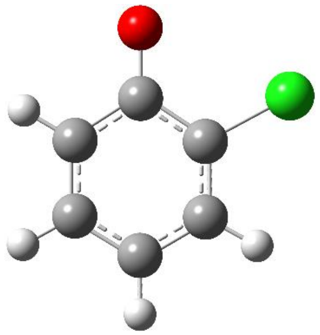

4-(methylthio)phenol

Charge=0, Multiplicity=1

C	0.57240000	0.27442300	0.00008000
C	-0.30393700	1.36676300	-0.00007700
C	-1.67650800	1.17546000	-0.00009700
C	-2.19613800	-0.11809300	-0.00000500
C	-1.33796600	-1.20966700	0.00013900
C	0.04249200	-1.01455600	0.00019800
H	0.08988100	2.37762800	-0.00019900
H	-2.35290000	2.02229000	-0.00022300
H	-1.74546800	-2.21524900	0.00020900
H	0.68701400	-1.88400900	0.00032800
O	-3.56406300	-0.25272700	-0.00007600
H	-3.80266200	-1.18832000	-0.00026200
S	2.31311000	0.63392100	0.00016100
C	3.05537400	-1.01454400	-0.00032800
H	2.77971800	-1.56996800	-0.89633200
H	4.13291500	-0.85156600	-0.00039800
H	2.77994200	-1.57042900	0.89545100

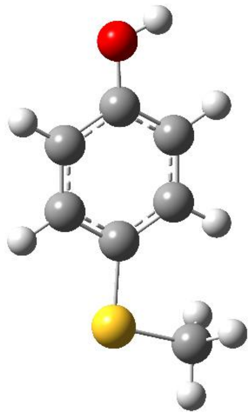

4-(methylthio)phenol anion

Charge=-1, Multiplicity=1

C	0.53841400	-0.00002700	-0.32170100
C	-0.17352100	1.20071500	-0.21590000
C	-1.54400000	1.20621400	-0.00750800
C	-2.29261900	0.00001200	0.10880200
C	-1.54402100	-1.20621100	-0.00743300
C	-0.17354100	-1.20075000	-0.21582300
H	0.35913700	2.14313300	-0.29909900
H	-2.07684500	2.14893500	0.07104000
H	-2.07688300	-2.14891800	0.07117200
H	0.35910100	-2.14318200	-0.29896000
O	-3.57614400	0.00003000	0.30583700
S	2.30013400	-0.00005100	-0.58234500
C	2.88715300	0.00008900	1.14183100
H	2.53886400	-0.89370800	1.65894900
H	3.97756800	0.00008600	1.11530300
H	2.53886600	0.89397000	1.65880500

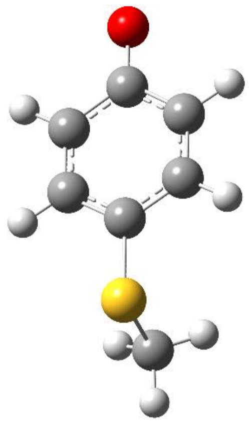

4-aminophenol

Charge=0, Multiplicity=1

C	-1.40892000	0.02200700	0.00220900
C	-0.69817600	1.21736600	-0.00011600
C	0.69118600	1.19997600	-0.00777500
C	1.39510500	-0.00871400	-0.01096200
C	0.66680000	-1.20098500	-0.00773600
C	-0.72467400	-1.18823000	-0.00033900
H	-1.23773100	2.15755500	0.00557800
H	1.24120500	2.13522800	-0.00923200
H	1.19571600	-2.14829700	-0.00915700
H	-1.28150800	-2.11969100	0.00522500
O	-2.78854600	0.09174700	0.01004900
H	-3.15467100	-0.80089100	0.01038100
N	2.79821900	-0.02197500	-0.08211800
H	3.22262700	0.80472700	0.32092700
H	3.20726200	-0.85730300	0.31902500

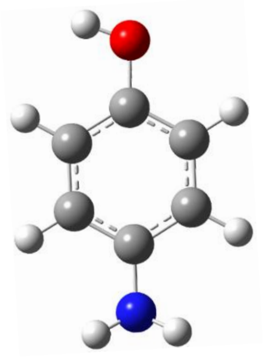

4-aminophenol anion

Charge=-1, Multiplicity=1

C	1.52436300	0.00000500	0.00359000
C	0.76541900	-1.19680300	-0.00085600
C	-0.62682400	-1.19445900	-0.01039900
C	-1.34934800	-0.00000700	-0.01275300
C	-0.62683500	1.19444800	-0.01038300
C	0.76541200	1.19680500	-0.00086200
H	1.29824400	-2.14328300	0.00543500

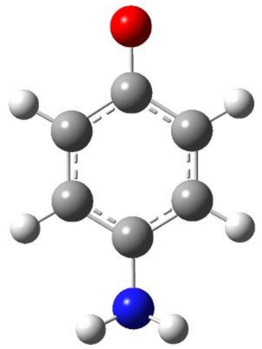

H	-1.16584400	-2.13817700	-0.01299400
H	-1.16586200	2.13816200	-0.01295000
H	1.29822700	2.14329000	0.00543100
O	2.84189300	0.00000700	0.01398600
N	-2.76894200	-0.00003200	-0.08741600
H	-3.16523000	-0.82290600	0.35281800
H	-3.16522000	0.82314400	0.35226300

ketobemidone

Charge=0, Multiplicity=1

C	-3.78957900	-0.03917400	-0.67698800
C	-3.00302000	0.98096600	-1.20903700
C	-1.64560900	1.04817300	-0.92923200
C	-1.04074600	0.09204000	-0.10075600
C	-1.82462700	-0.92390600	0.43420200
C	-3.18874400	-0.98491200	0.14347500
H	-4.85109100	-0.10302700	-0.89078100
H	-3.46132600	1.72795500	-1.84693300
H	-1.04630800	1.85025500	-1.34901900
H	-1.41267900	-1.68976400	1.07929300
O	-3.89539100	-2.01830600	0.70759900
H	-4.82494100	-1.95450100	0.45474400
C	0.46075700	0.22039700	0.15491100
C	0.68638400	1.52681600	0.92974100
C	1.80308600	2.47726700	0.57277300
H	2.20184900	2.87459700	1.50793800
H	2.60464800	1.97435000	0.03453300
C	1.23528700	3.62850200	-0.27263600
H	0.43541700	4.13924900	0.26692700
H	2.02377800	4.35132100	-0.48637600
H	0.83481500	3.27063100	-1.22341700
O	-0.04832300	1.79159700	1.86633500
C	1.21301900	0.13114500	-1.20666200
H	0.60375100	-0.46042100	-1.89483500
H	1.32131800	1.11714700	-1.66151500
C	1.03757200	-0.88060400	1.08400200
H	0.36008400	-1.07675800	1.91551300
H	1.97491900	-0.52574300	1.52659300
C	1.32135200	-2.13675800	0.26489400
H	0.42314600	-2.41364900	-0.29425700
H	1.55852600	-2.97541600	0.92119000
C	2.57537100	-0.53842800	-1.07809800
H	3.19127400	0.00493400	-0.34671900
H	3.10505700	-0.48856800	-2.03217100

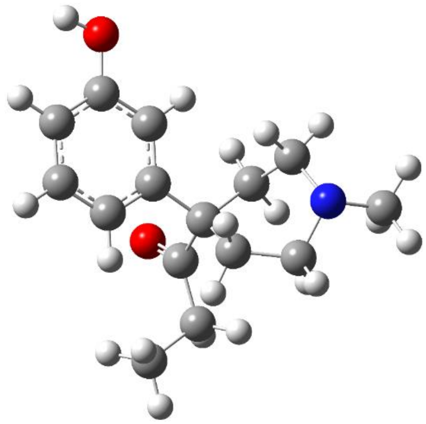

N	2.44621400	-1.95325800	-0.68026700
C	3.70341700	-2.37832900	-0.07013600
H	3.67215900	-3.44541100	0.15464900
H	4.52494200	-2.19968000	-0.76722900
H	3.92336300	-1.83019400	0.85955500

ketobemidone anion

Charge=-1, Multiplicity=1

C	-3.82306500	-0.16916300	-0.64852700
C	-3.07163700	0.87368600	-1.17816500
C	-1.70968100	0.99028600	-0.91539500
C	-1.08163000	0.03985100	-0.09373700
C	-1.83053400	-1.00173800	0.43793000
C	-3.22608900	-1.15139700	0.18248500
H	-4.88440300	-0.24821200	-0.86294500
H	-3.55842800	1.61197800	-1.80828700
H	-1.13794400	1.81107200	-1.33646600
H	-1.37293500	-1.75335300	1.07171000
O	-3.90672500	-2.14060700	0.69188800
C	0.42042100	0.21181800	0.15611400
C	0.61316300	1.52244600	0.93082500
C	1.67238600	2.52874700	0.54719000
H	2.06751200	2.94922100	1.47377300
H	2.48916100	2.06525400	-0.00372100
C	1.03619900	3.64858800	-0.29128400
H	0.20778900	4.10864000	0.25105600
H	1.78026100	4.41828300	-0.50114100
H	0.65771700	3.27343100	-1.24415900
O	-0.09729600	1.74871700	1.89696000
C	1.17177200	0.14859000	-1.20639700
H	0.58552900	-0.47158400	-1.88934600
H	1.23615800	1.13565200	-1.66753600
C	1.04000500	-0.86974700	1.08155100
H	0.36857600	-1.09597600	1.90986600
H	1.96264000	-0.48125100	1.52746400
C	1.37460800	-2.11148000	0.26035900
H	0.48839800	-2.42122100	-0.30005800
H	1.64483600	-2.94225200	0.91414400
C	2.56201500	-0.46151000	-1.08055800
H	3.15457200	0.10693700	-0.34874100
H	3.08954800	-0.38921100	-2.03456600
N	2.49301000	-1.88111600	-0.68321600
C	3.76564000	-2.25232800	-0.07041300

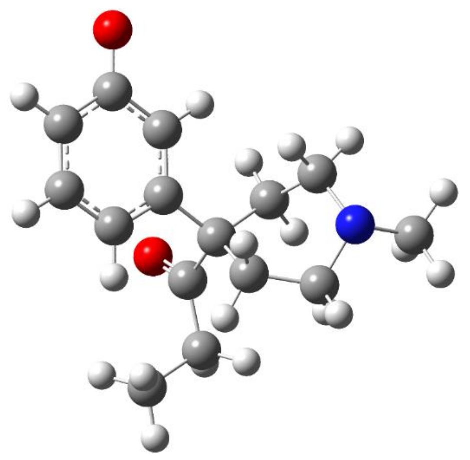

H	3.77983100	-3.31990000	0.15408100
H	4.58069400	-2.03858800	-0.76533200
H	3.96015200	-1.69588100	0.86005900

profadol

Charge=0, Multiplicity=1

C	-3.72848500	-0.85356300	-0.01564600
C	-2.77490500	-1.85378300	0.15659800
C	-1.42228100	-1.54614300	0.21137300
C	-0.97792000	-0.22111700	0.09251000
C	-1.93578600	0.77717700	-0.08750500
C	-3.29456600	0.45883800	-0.13730300
H	-4.78769400	-1.07780800	-0.05757300
H	-3.09649000	-2.88494300	0.24961700
H	-0.69996900	-2.34288600	0.34487300
H	-1.65574300	1.81803800	-0.19743500
O	-4.25049000	1.43188300	-0.31037000
H	-3.82720600	2.29707000	-0.37504300
C	0.81109800	1.57058800	-0.04591000
H	0.44778200	1.87821500	-1.03452800
H	0.22477800	2.12841900	0.69292800
C	2.26656700	2.01929000	0.09775200
H	2.63032800	1.78942700	1.10351800
H	2.91250400	1.47785800	-0.59903300
C	2.39988900	3.51949900	-0.15487900
H	3.43379400	3.85246800	-0.03961200
H	2.07553000	3.77429700	-1.16763600
H	1.78325100	4.08945700	0.54579700
C	1.15220300	-0.45884400	1.46652900
H	1.33148200	0.34959800	2.17752400
H	0.48827900	-1.18277300	1.94070400
C	1.26554300	-0.77171000	-0.94532300
H	1.94052700	-0.12017300	-1.51824000
H	0.57833400	-1.23341400	-1.65710000
C	0.52534000	0.06756500	0.13921600
C	2.42993700	-1.15968900	1.02464400
H	2.78471700	-1.90278000	1.74202200
H	3.24172300	-0.43509300	0.85670400
N	2.03680400	-1.80241700	-0.23348800
C	3.19190900	-2.22811900	-1.00949400
H	3.76005500	-2.97792600	-0.45526300
H	2.86835800	-2.67284300	-1.95276600
H	3.86069500	-1.38228900	-1.23745800

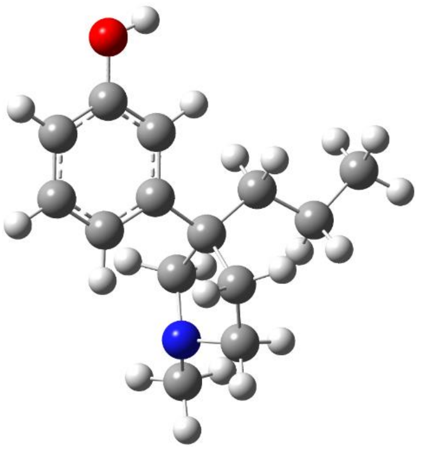

profadol anion

Charge=-1, Multiplicity=1

C	-3.78236100	-0.76119900	-0.02788200
C	-2.85052600	-1.77857600	0.15187800
C	-1.48706300	-1.51140800	0.21417200
C	-1.02519000	-0.18836800	0.09046000
C	-1.96053100	0.82578200	-0.09493500
C	-3.36548900	0.58607500	-0.15858300
H	-4.84389800	-0.98464400	-0.07194600
H	-3.19658400	-2.80367200	0.24676700
H	-0.78284200	-2.32294700	0.35409200
H	-1.64958400	1.85813000	-0.20470200
O	-4.21452900	1.56528000	-0.32960300
C	0.81184000	1.56559200	-0.04605100
H	0.45795900	1.88112500	-1.03537700
H	0.23630100	2.13848300	0.68918200
C	2.27704900	1.98259600	0.09837900
H	2.63525100	1.74944700	1.10545900
H	2.91227500	1.42463200	-0.59545300
C	2.44499300	3.47853800	-0.16010100
H	3.48583700	3.78911400	-0.04370900
H	2.12874600	3.73646600	-1.17466300
H	1.83948300	4.06529100	0.53643700
C	1.10521300	-0.46704600	1.46682300
H	1.29593600	0.33787800	2.17922400
H	0.42850200	-1.18026500	1.93882100
C	1.21545200	-0.78279300	-0.94365800
H	1.89490300	-0.14304200	-1.52501500
H	0.51898800	-1.24120000	-1.64839600
C	0.48786400	0.06966700	0.13927200
C	2.37417100	-1.18746200	1.03057400
H	2.71249800	-1.93912400	1.74736500
H	3.19937900	-0.47628500	0.87137600
N	1.98022100	-1.81991800	-0.23250100
C	3.13289800	-2.25583500	-1.00570200
H	3.69329400	-3.01077500	-0.45029300
H	2.80767200	-2.69800500	-1.94970500
H	3.81064400	-1.41679800	-1.23301800

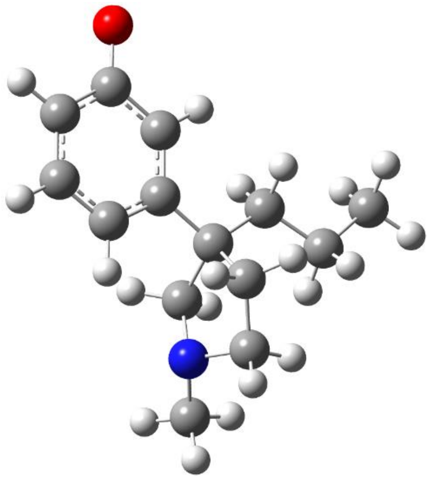

tapentadol

Charge=0, Multiplicity=1

C	3.26237900	-0.74010400	-0.66302200
C	4.04771300	-0.44606900	0.45084300
C	3.49623200	0.30617500	1.47937100
C	2.17871900	0.75801300	1.40564000
C	1.39310600	0.46116000	0.29220800
C	1.94902600	-0.29176100	-0.74640600
H	5.06917500	-0.80507500	0.49448500
H	4.09947100	0.54136600	2.34899600
H	1.75647000	1.33906100	2.21833600
H	1.36079200	-0.53363300	-1.62809500
O	3.83915300	-1.48455300	-1.66355200
H	3.19874200	-1.62759800	-2.37177000
C	-0.04567600	0.92805600	0.19891300
H	-0.24399100	1.54180900	1.08681600
C	-0.25486200	1.81290700	-1.05641400
H	0.71960900	2.15403200	-1.41532000
H	-0.68139400	1.20731500	-1.86340100
C	-1.12094400	3.04628300	-0.80208100
H	-0.65310900	3.68755300	-0.05001200
H	-1.23695800	3.63365200	-1.71626000
H	-2.11778400	2.78671300	-0.44167600
C	-1.00425000	-0.29019000	0.25955100
H	-0.77348800	-0.91505300	-0.61212000
C	-0.77314000	-1.10647400	1.53009300
H	0.26344800	-1.44121400	1.60521800
H	-0.99949100	-0.50263800	2.41593200
H	-1.41259300	-1.98992100	1.55313600
C	-2.45994100	0.17270800	0.15555100
H	-2.64525600	0.92729800	0.92857600
H	-2.61677200	0.65915400	-0.82172100
N	-3.46374100	-0.88565300	0.32312500
C	-3.28485100	-1.92079700	-0.69026800
H	-4.10738300	-2.63555900	-0.62899500
H	-3.27170700	-1.49744700	-1.70883900
H	-2.35325300	-2.46524100	-0.53141600
C	-4.78590000	-0.28875100	0.15789400
H	-4.91503500	0.14975400	-0.84536900
H	-5.55833000	-1.04635800	0.30196700
H	-4.93385700	0.50109100	0.89682300

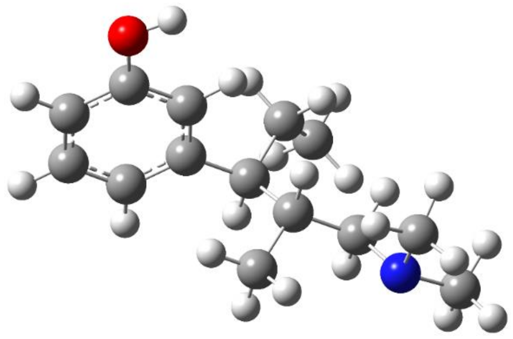

tapentadol anion

Charge=-1, Multiplicity=1

C	3.30794800	-0.80086800	-0.76894600
C	4.08011300	-0.48587700	0.38526400
C	3.54519100	0.25612700	1.42775700
C	2.22654000	0.71812700	1.38626300
C	1.43393100	0.42482500	0.27231600
C	1.97278000	-0.31773200	-0.77789300
H	5.10621900	-0.83849100	0.42945600
H	4.16354600	0.48051600	2.29192400
H	1.81573900	1.29267100	2.20959200
H	1.36114900	-0.54298700	-1.64890400
O	3.80185100	-1.49371100	-1.75991400
C	-0.00617800	0.90274900	0.20418900
H	-0.19429500	1.49379900	1.11041000
C	-0.22438300	1.82638200	-1.02146900
H	0.74891000	2.16989000	-1.38100100
H	-0.66425200	1.24887200	-1.84206900
C	-1.07774300	3.05928400	-0.72338200
H	-0.59717900	3.67435300	0.04265200
H	-1.19757400	3.67511600	-1.61834900
H	-2.07356600	2.79860400	-0.36071400
C	-0.97862200	-0.30508900	0.24013900
H	-0.76297900	-0.90824900	-0.65018900
C	-0.74470600	-1.15980900	1.48465600
H	0.29138700	-1.49922900	1.54286000
H	-0.96327600	-0.58151800	2.38949000
H	-1.38743800	-2.04145400	1.48622200
C	-2.43077500	0.17403300	0.16420400
H	-2.60145500	0.90858000	0.95970800
H	-2.59270500	0.68928800	-0.79741000
N	-3.44488000	-0.87796300	0.31237300
C	-3.28662700	-1.88657000	-0.73066000
H	-4.11518100	-2.59526700	-0.68015200
H	-3.28021100	-1.43553000	-1.73737500
H	-2.35838500	-2.44326000	-0.59709000
C	-4.76199000	-0.26320900	0.17624800
H	-4.89589900	0.20422300	-0.81330600
H	-5.54125400	-1.01631300	0.30691200
H	-4.89482300	0.50756900	0.93791100

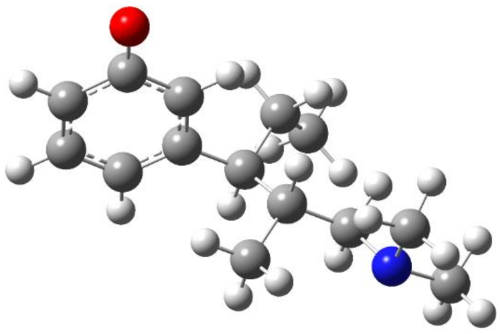

(R)-Trolox

Charge=-1, Multiplicity=1

C	2.41254900	-0.85214600	0.01996600
C	1.12909500	-1.37664200	-0.17205800
C	0.05896000	-0.49096500	-0.36068600
C	0.23395300	0.89349000	-0.33116600
C	1.51009000	1.41800900	-0.07948000
C	2.57614700	0.53695700	0.08336200
O	-1.17249000	-1.06579100	-0.60441900
C	-0.93742200	1.82085800	-0.56143000
H	-1.22512800	2.28693500	0.38889900
H	-0.63761500	2.63572900	-1.22476300
C	-2.12645400	1.07226400	-1.15110200
H	-3.03378200	1.67362800	-1.09350400
H	-1.94357300	0.83298800	-2.20328400
C	-2.33054400	-0.24564500	-0.41167400
O	3.82504000	1.09275900	0.30222100
H	4.46064000	0.40113700	0.51868300
C	1.71886700	2.90802400	-0.00792700
H	1.71520500	3.35416300	-1.00775500
H	0.91975900	3.38574000	0.56253500
H	2.66880700	3.15231800	0.46360500
C	3.62262900	-1.74061500	0.16570600
H	4.45082600	-1.37732400	-0.45023600
H	3.97405900	-1.77557000	1.20266400
H	3.41431700	-2.76052300	-0.14971300
C	0.88733000	-2.86543600	-0.17839500
H	-0.16682300	-3.08703700	-0.02484900
H	1.18955300	-3.31561400	-1.12929200
H	1.45570500	-3.35679700	0.61249900
C	-3.49343100	-1.04560800	-0.97914700
H	-3.59033800	-2.00070900	-0.45723100
H	-4.42191800	-0.48537300	-0.86497000
H	-3.32423800	-1.23788000	-2.04103600
C	-2.58357400	-0.00575300	1.10277400
O	-1.89689500	-0.64507000	1.93284900
O	-3.50248200	0.80728200	1.37751100

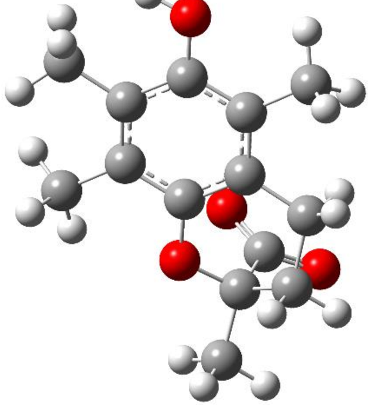

(R)-Trolox anion

Charge=-2, Multiplicity=1

C	-0.08682300	0.47002300	-0.37811900
C	-0.27216800	-0.91328500	-0.32696300
C	-2.66543800	-0.55865200	0.09895300
O	-3.87556000	-1.03605500	0.30889300
C	0.90207100	-1.84509200	-0.55641100
H	1.18404200	-2.31917600	0.39109600
H	0.60697700	-2.65451600	-1.22830800
C	2.10347200	-1.10730600	-1.13580900
H	1.93748700	-0.88212300	-2.19424900
H	3.00704400	-1.71280100	-1.05969700
C	2.30190800	0.22460400	-0.42072300
C	3.48270900	1.00079000	-0.98833000
H	3.57612500	1.97006400	-0.49241000
H	4.40599200	0.43874900	-0.84138700
H	3.33524800	1.16400300	-2.05846300
C	2.54393500	0.03496300	1.10324200
O	1.15507000	1.04052800	-0.65532500
C	-1.15201600	1.35902200	-0.18062200
C	-2.43766900	0.84552100	0.02445600
C	-1.55127400	-1.42395700	-0.05876000
C	-3.63501800	1.74857600	0.17913600
H	-4.47109800	1.37246400	-0.41561800
H	-3.98462400	1.78200200	1.21762000
H	-3.42880900	2.77020700	-0.13749200
C	-0.89522300	2.84842700	-0.19230000
H	0.16142800	3.06262400	-0.04135800
H	-1.19462000	3.30370900	-1.14219400
H	-1.45668000	3.34861400	0.59839600
C	-1.79034300	-2.90815800	0.05479500
H	-2.23110900	-3.32046700	-0.86089100
H	-0.87373900	-3.46107500	0.25897500
H	-2.49759700	-3.11618900	0.86051200
O	3.38024100	-0.84893800	1.42205400
O	1.94464900	0.79459800	1.90065300

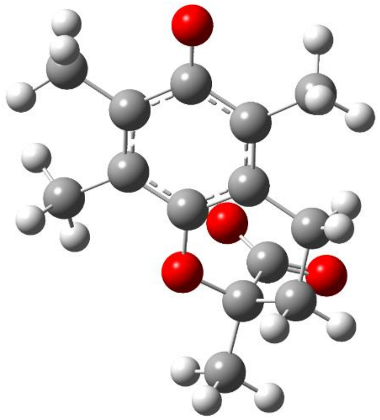

(R)-Trolox Ref 32's methodology

Charge=-1, Multiplicity=1

C	-1.26718500	1.42674500	-0.21783500
C	0.11140200	1.70942800	-0.29122600
C	1.02122000	0.63964400	-0.39014000
C	0.59460700	-0.69582600	-0.41756100
C	-0.77938600	-0.98268200	-0.28797800
C	-1.68373200	0.08343000	-0.17510000
O	2.36138600	0.98732500	-0.49845800
C	1.60502900	-1.81271200	-0.57983000
H	1.73368100	-2.33954900	0.37628300
H	1.22585500	-2.56025800	-1.28476800
C	2.95553400	-1.28786500	-1.07051700
H	3.72688600	-2.05505100	-0.97240500
H	2.89145100	-1.01467300	-2.13081900
C	3.36925600	-0.03313400	-0.29582300
O	-3.03113000	-0.25183800	-0.05499400
H	-3.60243000	0.48519900	0.26194400
C	-1.25065500	-2.41908000	-0.28933800
H	-1.09084200	-2.88780300	-1.26894000
H	-0.69637600	-3.01803100	0.44196100
H	-2.31127300	-2.50038300	-0.05463500
C	-2.28066700	2.54985300	-0.18897700
H	-3.20475800	2.27133100	-0.70391700
H	-2.55031200	2.83757800	0.83531600
H	-1.89476800	3.44158400	-0.68633000
C	0.61036300	3.13746800	-0.27523100
H	1.67748600	3.17883400	-0.05538600
H	0.45446300	3.63244400	-1.24303000
H	0.08758500	3.73361200	0.47875500
C	4.66127900	0.57784100	-0.84423600
H	4.91194500	1.50022300	-0.31000900
H	5.48921300	-0.12610100	-0.73106000
H	4.54185700	0.81012100	-1.90741800
C	3.56334700	-0.32961900	1.22721600
O	2.98050700	0.40806000	2.07342200
O	4.34207300	-1.29247500	1.50609600
O	-5.07426300	1.22915900	1.05203700
H	-5.61719300	0.48012200	1.34148300
H	-5.62124500	1.69743000	0.40363300
O	-4.16921400	-1.40649300	-2.37502100
H	-3.76010500	-1.02645200	-1.56958200
H	-3.66167400	-2.21161600	-2.55083300

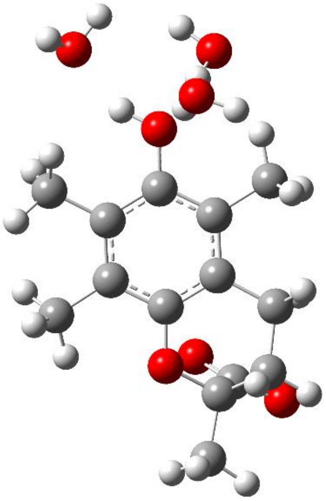

O	-3.91076500	-1.81976300	2.26430800
H	-3.56778100	-1.33498000	1.48874100
H	-4.53289400	-1.20632800	2.68135300

(R)-Trolox anion Ref 32's methodology

Charge=-2, Multiplicity=1

C	-1.31966400	1.34217800	-0.03263200
C	0.04173200	1.63640300	-0.24371400
C	0.95928100	0.57818000	-0.37034700
C	0.54824100	-0.76095700	-0.29567300
C	-0.81208800	-1.05337600	-0.06232000
C	-1.76562300	-0.00695000	0.03180100
O	2.29013800	0.94051700	-0.59594400
C	1.56860900	-1.87004200	-0.47909800
H	1.79667100	-2.33914400	0.48861300
H	1.15083600	-2.66434600	-1.10660100
C	2.85919600	-1.35229300	-1.11633600
H	3.64896300	-2.10504800	-1.05776400
H	2.69214700	-1.12873900	-2.17734000
C	3.32284800	-0.05774500	-0.44139200
O	-3.07779500	-0.28582600	0.18762100
C	-1.26933800	-2.48730200	0.09069900
H	-1.61473200	-2.92031900	-0.85883700
H	-0.46967100	-3.13062700	0.46619800
H	-2.10518100	-2.55313100	0.79222800
C	-2.30812600	2.47540100	0.13302100
H	-2.52121100	2.98080300	-0.81911800
H	-3.25737300	2.11431500	0.53089700
H	-1.92580400	3.24238200	0.81595300
C	0.50133700	3.07578100	-0.33484000
H	1.52931500	3.15102500	-0.69022800
H	-0.13350200	3.65239100	-1.01676300
H	0.45142500	3.57689500	0.64115500
C	4.55394800	0.53585800	-1.13296300
H	4.84033700	1.48527200	-0.66829200
H	5.39803600	-0.15444900	-1.06292100
H	4.33620100	0.71609800	-2.19072300
C	3.66411900	-0.28343800	1.06808600
O	3.15263600	0.48456700	1.93350000
O	4.48211600	-1.22288700	1.31644000
H	-3.95853900	-1.66794300	-0.26905100
O	-4.62012500	-2.36763100	-0.53041800
H	-4.14173500	-3.20453000	-0.45432400

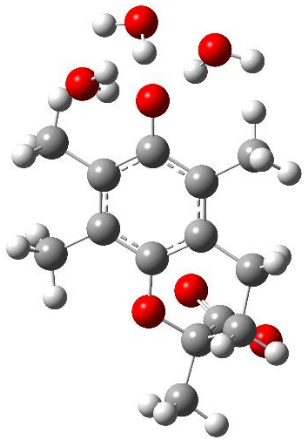

O	-4.03089200	0.20129100	2.61036800
H	-3.66103400	0.04620200	1.68906500
H	-4.02994100	1.16228600	2.72056000
O	-4.70568900	0.82620900	-1.63220300
H	-4.08082100	0.44723800	-0.94797900
H	-5.13432400	0.05325500	-2.02539400

Δ⁹-tetrahydrocannabinol (Δ⁹-THC)

Charge=0, Multiplicity=1

C	-1.20654100	-1.27814300	0.24657700
C	0.16551600	-1.03130200	0.15745600
C	0.74009500	0.14725500	0.64775300
C	-0.11555300	1.01040200	1.34835700
C	-1.48259300	0.77996600	1.44659400
C	-2.04271600	-0.36505600	0.87701500
H	-1.59933000	-2.20107200	-0.16612700
H	-2.10323800	1.48863100	1.98757200
O	0.45631500	2.09106600	1.97073900
H	-0.22925500	2.59464200	2.42739700
O	0.90775900	-2.01958500	-0.43337100
C	2.22555600	0.40481600	0.49674100
H	2.68634700	0.25947600	1.48518100
C	2.84232600	-0.61222400	-0.47160900
H	2.46795500	-0.37630600	-1.47769600
C	2.34291900	-2.02730400	-0.18194300
C	2.59192000	1.79599400	0.01059900
C	3.70363000	2.04271100	-0.68875800
H	1.92761100	2.61775300	0.25240300
C	4.68130600	0.95931800	-1.07656000
H	5.69172500	1.27194000	-0.79253000
H	4.68715300	0.89455200	-2.17226600
C	4.35552600	-0.41312700	-0.47772000
H	4.85792400	-1.19009200	-1.05719500
H	4.73034600	-0.47515200	0.54931500
C	2.59101000	-2.48923000	1.24945900
H	2.25283100	-3.52118100	1.36770400
H	3.65987100	-2.44831200	1.47203300
H	2.06374500	-1.86716600	1.97563000
C	2.88854800	-3.03983900	-1.17449900
H	2.78763300	-2.67179000	-2.19851100
H	3.94142700	-3.24202500	-0.97390600
H	2.33730300	-3.97789000	-1.07884100
C	4.05411200	3.42325200	-1.16793700

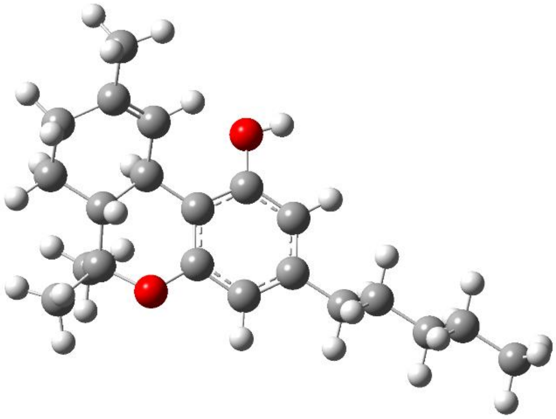

H	5.00819800	3.74793100	-0.74061900
H	4.17616500	3.43086200	-2.25599700
H	3.28664400	4.15043000	-0.89788900
C	-3.53269300	-0.58547700	0.92476700
H	-3.92594600	-0.22919200	1.88186600
H	-3.74717000	-1.65600900	0.85826300
C	-4.25439800	0.14485800	-0.21455100
H	-4.02999900	1.21587800	-0.15268600
H	-3.85724900	-0.20714800	-1.17360100
C	-5.76512700	-0.06383200	-0.17763200
H	-6.15539200	0.28387800	0.78663900
H	-5.98494800	-1.13682400	-0.23490200
C	-6.49411900	0.66058500	-1.30651900
H	-6.27081200	1.73141000	-1.24890400
H	-6.10338800	0.31143500	-2.26842100
C	-8.00399300	0.44677200	-1.25651700
H	-8.41680500	0.81228400	-0.31205100
H	-8.51162400	0.97085600	-2.06950900
H	-8.24870600	-0.61602000	-1.33773400

Δ⁹-tetrahydrocannabinol anion

Charge=-1, Multiplicity=1

C	-1.22732900	-1.22241200	0.27294700
C	0.14686200	-0.98114300	0.17583300
C	0.74260400	0.17848300	0.67227100
C	-0.05903000	1.08540500	1.44181400
C	-1.45623600	0.83540200	1.50011100
C	-2.03597700	-0.29076000	0.92380100
H	-1.63840400	-2.13688600	-0.14086300
H	-2.07373600	1.53942900	2.05224500
O	0.47452100	2.08516700	2.08240900
O	0.87348200	-1.98182200	-0.44082500
C	2.22981000	0.40468200	0.50583400
H	2.70593400	0.25579900	1.48795400
C	2.83117700	-0.61307500	-0.47046500
H	2.45832500	-0.36442300	-1.47448400
C	2.30443700	-2.02043300	-0.19220200
C	2.60918800	1.79082000	0.01743100
C	3.72105900	2.02941600	-0.68510400
H	1.94422200	2.61293000	0.25878900
C	4.69081900	0.93693900	-1.07140300
H	5.70341700	1.23712000	-0.78074100
H	4.70295000	0.87710400	-2.16757100

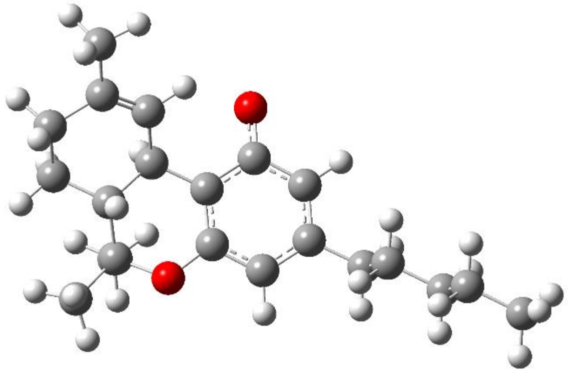

C	4.34733000	-0.43676100	-0.48241700
H	4.83766000	-1.21541800	-1.07060000
H	4.72620800	-0.51268200	0.54231300
C	2.55225500	-2.49858900	1.23473900
H	2.19050900	-3.52324300	1.34911000
H	3.62327900	-2.48523300	1.45113200
H	2.04432800	-1.86805300	1.96707700
C	2.83211400	-3.03586100	-1.19274000
H	2.73239900	-2.66063500	-2.21446900
H	3.88306100	-3.25492000	-0.99892200
H	2.26789800	-3.96685600	-1.10155100
C	4.08059400	3.40511600	-1.17317700
H	5.03914400	3.72600400	-0.75255000
H	4.19698600	3.40828300	-2.26208000
H	3.32002100	4.13944900	-0.90241800
C	-3.53144000	-0.49248800	0.97656200
H	-3.92688600	-0.08115200	1.91074300
H	-3.75931600	-1.56321700	0.96885900
C	-4.24880200	0.17617400	-0.20256000
H	-4.02423800	1.24903100	-0.19663800
H	-3.84818900	-0.22541300	-1.14060700
C	-5.76018900	-0.02927300	-0.16377400
H	-6.15580800	0.36594500	0.77989300
H	-5.98034900	-1.10386600	-0.16823000
C	-6.48438500	0.63711500	-1.33091300
H	-6.26446200	1.71017800	-1.32414100
H	-6.08662200	0.24306500	-2.27241300
C	-7.99402900	0.42141200	-1.28035300
H	-8.41417900	0.83092300	-0.35740800
H	-8.49790200	0.90380700	-2.12106200
H	-8.23525800	-0.64485100	-1.31144100

Δ⁹-tetrahydrocannabinol (Δ⁹-THC) Ref 32's methodology

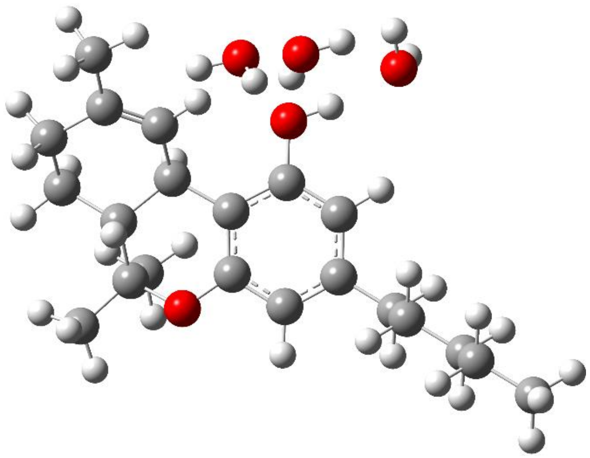

Charge=0, Multiplicity=1

O	1.11535500	4.15103800	-1.76634900
H	0.83301900	4.93709700	-1.27936600
H	0.88940600	4.33724200	-2.68736500
O	-2.67401900	2.64762100	-2.49070500
H	-1.94542300	2.41225800	-1.88670100
H	-3.39769300	2.06749100	-2.22427600
O	0.05352500	3.97279700	1.62202200
H	-0.17782600	3.31837900	0.94452200

H	0.67219100	4.56424800	1.17568100
C	1.31367000	-1.63396300	-0.56916000
C	-0.06193500	-1.47765800	-0.36772000
C	-0.67816600	-0.21526800	-0.34399000
C	0.15138700	0.88008400	-0.65541600
C	1.52367100	0.73635500	-0.85875600
C	2.12350300	-0.52548700	-0.80097200
H	1.72934200	-2.63561400	-0.56368300
H	2.11989800	1.61457000	-1.08052500
O	-0.42990700	2.12519800	-0.77655000
H	0.20708600	2.77881000	-1.15726500
O	-0.76245700	-2.65046100	-0.19969200
C	-2.17730700	-0.10341600	-0.09644700
H	-2.64490000	0.12035700	-1.06733300
C	-2.75523800	-1.44284800	0.40552600
H	-2.37618700	-1.59824600	1.42444300
C	-2.22137100	-2.63655400	-0.39923200
C	-2.60352800	0.98719200	0.87615300
C	-3.71806400	0.91773800	1.61614900
H	-1.97943200	1.86805100	0.95453400
C	-4.64959200	-0.27199600	1.56840600
H	-5.67715100	0.07933100	1.41565900
H	-4.64864000	-0.74376900	2.56088700
C	-4.27977200	-1.30968700	0.49697800
H	-4.74701900	-2.26554100	0.74367700
H	-4.67589800	-1.00049600	-0.47628800
C	-2.51101600	-2.57363000	-1.90025700
H	-2.09646700	-3.45505300	-2.39616200
H	-3.58976100	-2.56120000	-2.07415500
H	-2.08160400	-1.68475000	-2.36629200
C	-2.67431300	-3.97329900	0.18016700
H	-2.50502800	-4.00905800	1.25953800
H	-3.73566800	-4.13822600	-0.01208100
H	-2.11631400	-4.78795300	-0.28841500
C	-4.11206100	2.01542400	2.56862900
H	-5.08701700	2.43758300	2.29747500
H	-4.21408900	1.62750000	3.58930800
H	-3.37910500	2.82528600	2.58113900
C	3.61766100	-0.67603700	-0.97503800
H	3.97553700	0.06325000	-1.69947200
H	3.83657800	-1.66502100	-1.39047000
C	4.39992200	-0.50221000	0.34061700
H	4.17865300	0.48567400	0.76187000

H	4.04295900	-1.23956300	1.06959000
C	5.91323200	-0.65303800	0.15931000
H	6.26469600	0.08253300	-0.57583500
H	6.13004800	-1.64136700	-0.26635700
C	6.70389900	-0.48113200	1.46051600
H	6.48720700	0.50634900	1.88573200
H	6.35250300	-1.21587800	2.19504600
C	8.21492500	-0.63313100	1.27138900
H	8.60190900	0.11064700	0.56696000
H	8.75081600	-0.50529300	2.21675100
H	8.46634800	-1.62380800	0.87831000

Δ⁹-tetrahydrocannabinol anion Ref 32's methodology

Charge=-1, Multiplicity=1

C	-1.34479400	-1.51453700	0.67665900
C	0.03357900	-1.39251900	0.46569000
C	0.66966500	-0.15055900	0.33377200
C	-0.11547100	1.01979500	0.56446900
C	-1.50848700	0.88023200	0.75548900
C	-2.13138700	-0.36818600	0.79976700
H	-1.77921300	-2.50501100	0.75967200
H	-2.09437500	1.78299100	0.89956000
O	0.43888300	2.22935400	0.61669500
O	0.71775800	-2.59507300	0.39761900
C	2.16358800	-0.09184100	0.04903100
H	2.66480200	0.21760900	0.97891100
C	2.71670600	-1.47842800	-0.33727900
H	2.31844000	-1.72250400	-1.33152800
C	2.17588600	-2.58368200	0.58158900
C	2.57404600	0.89296000	-1.03658100
C	3.67468200	0.74366000	-1.78599400
H	1.94249600	1.75957400	-1.18995500
C	4.60397400	-0.44024600	-1.63267600
H	5.63422800	-0.08048900	-1.52166600
H	4.59413800	-1.00263400	-2.57692300
C	4.24074300	-1.37486600	-0.46652200
H	4.69395800	-2.35444800	-0.63548500
H	4.65787000	-0.98594600	0.46871600
C	2.48682400	-2.38275500	2.06672400
H	2.06798000	-3.20729300	2.64964300
H	3.56783400	-2.36805100	2.22695300
H	2.07371100	-1.44782300	2.44963800

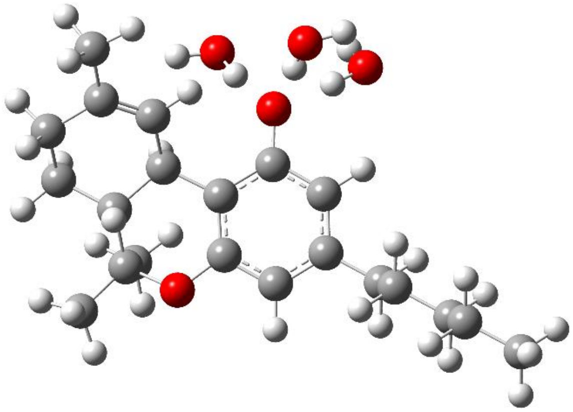

C	2.60796400	-3.97538900	0.12815200
H	2.42521000	-4.11230100	-0.94104200
H	3.67001300	-4.13405000	0.32279400
H	2.04715500	-4.73575300	0.67799800
C	4.05394000	1.73292600	-2.85645700
H	5.03447200	2.17849800	-2.64984200
H	4.13643900	1.23999100	-3.83279400
H	3.32242400	2.53992900	-2.94081200
C	-3.63141900	-0.47839500	0.97019200
H	-3.98960800	0.34995600	1.59106600
H	-3.87098500	-1.40436400	1.50426300
C	-4.39885800	-0.46377900	-0.36543900
H	-4.15980900	0.45980900	-0.90615800
H	-4.04512200	-1.29184500	-0.99149300
C	-5.91624600	-0.57165500	-0.18619800
H	-6.26555600	0.25589000	0.44493800
H	-6.15183400	-1.49484400	0.35919200
C	-6.69106800	-0.55716200	-1.50811600
H	-6.45522600	0.36465700	-2.05358500
H	-6.34343000	-1.38463500	-2.13850000
C	-8.20610800	-0.66316700	-1.31932600
H	-8.58878000	0.16996800	-0.72045600
H	-8.73087300	-0.65023100	-2.27940500
H	-8.47634900	-1.59173400	-0.80555900
H	-0.00491000	3.29312900	-0.64354100
O	-0.23421700	3.91745200	-1.38063700
H	-0.87807800	4.52272400	-0.99473200
O	-0.67470400	4.02295300	2.31798800
H	-0.29259600	3.34641500	1.69896100
H	0.05646300	4.26253900	2.89929000
O	2.69438900	2.92380400	1.91200300
H	1.89964700	2.59902300	1.41215300
H	3.43604900	2.44888600	1.51869200

cannabidiol

Charge=0, Multiplicity=1

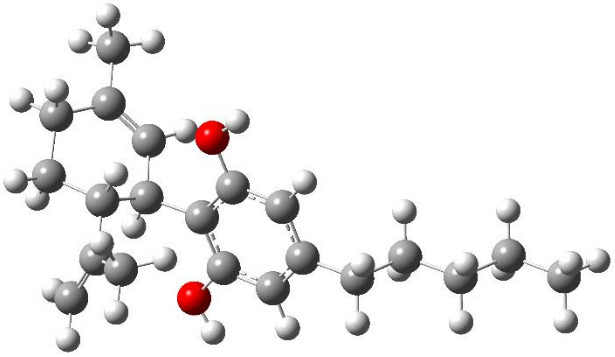

C	-1.22828000	0.27279800	-1.08390200
C	0.10689400	0.52051600	-0.77524500
C	0.74184400	-0.06949300	0.32584700
C	-0.04173900	-0.92644500	1.10751900
C	-1.37915500	-1.18777000	0.81211100
C	-1.98198400	-0.59021200	-0.29095700
H	-1.67168000	0.75156300	-1.95231900

H	-1.94219200	-1.86313400	1.44995500
O	0.55203200	-1.52142900	2.19333700
H	-0.09033200	-2.09411100	2.63043800
C	2.21592100	0.13519000	0.61744300
H	2.41660400	-0.35684300	1.57425800
C	3.11077600	-0.56987100	-0.43052900
H	2.86670300	-0.15110900	-1.41312200
C	2.59334000	1.59417200	0.76642500
C	3.75631700	2.11344200	0.36818800
H	1.85329700	2.24037300	1.23541200
C	4.82144300	1.26820900	-0.28765000
H	5.79682100	1.53802900	0.13136000
H	4.86874700	1.53294000	-1.35220300
C	4.57338800	-0.23018900	-0.12753700
H	5.23402700	-0.79633500	-0.78933400
H	4.80552700	-0.53172400	0.89952300
C	2.02768500	-2.54067500	-1.65686800
H	1.07054100	-2.00998700	-1.71253200
H	2.56256300	-2.32360000	-2.58732400
H	1.83365100	-3.61337500	-1.60526400
C	4.08042900	3.57261300	0.51929000
H	4.96299500	3.70941100	1.15222100
H	4.31957100	4.01221100	-0.45492600
H	3.24864000	4.12814500	0.95578200
C	-3.44116300	-0.82147900	-0.58784500
H	-3.73671300	-1.81010100	-0.22458500
H	-3.60170100	-0.80646400	-1.67007200
C	-4.33343500	0.24156200	0.06489900
H	-4.16701900	0.23280800	1.14822400
H	-4.03143300	1.23207100	-0.29417900
C	-5.81406600	0.02019400	-0.22812900
H	-6.11030000	-0.97323900	0.13000100
H	-5.97282000	0.02103400	-1.31337700
C	-6.71238200	1.07506800	0.41278800
H	-6.55158600	1.07336500	1.49628700
H	-6.41400000	2.06619000	0.05438000
C	-8.18992700	0.84256300	0.11164300
H	-8.51374100	-0.13298500	0.48510400
H	-8.81978400	1.60507400	0.57549500
H	-8.37468000	0.86450600	-0.96604300
C	2.82361100	-2.06062400	-0.47504500
C	3.23203900	-2.89004100	0.48595200
H	3.79956000	-2.53921800	1.34276600

H	3.00105700	-3.95007300	0.44757400
O	0.85803900	1.35088900	-1.56602500
H	0.30777200	1.69173300	-2.28238700

cannabidiol anion C1′ (anion 2)

Charge=-1, Multiplicity=1

C	-1.19743600	0.20959900	-1.21860600
C	0.17355200	0.49305800	-0.95895800
C	0.73699300	-0.01922400	0.25510700
C	-0.08402500	-0.76154600	1.10658300
C	-1.42498000	-1.03098900	0.82754900
C	-1.98474800	-0.53778700	-0.35351500
H	-1.62332400	0.59347400	-2.14213100
H	-2.01671100	-1.61639000	1.52545800
O	0.46976300	-1.25106500	2.27836900
H	-0.21093500	-1.73506000	2.76084900
C	2.20712800	0.16921600	0.58174700
H	2.37352300	-0.27265700	1.56955600
C	3.13578700	-0.60357900	-0.38652800
H	2.94759800	-0.22774800	-1.39741900
C	2.61661000	1.62509800	0.67299100
C	3.81013400	2.10022500	0.30936000
H	1.87101000	2.31245900	1.07038900
C	4.88540000	1.19942600	-0.24970800
H	5.84577000	1.46697600	0.20465400
H	4.99283900	1.40951100	-1.32220600
C	4.59138200	-0.28365100	-0.03061900
H	5.26967400	-0.89567200	-0.63149800
H	4.76829800	-0.53952700	1.01985000
C	2.05983000	-2.60141400	-1.57025500
H	1.12057800	-2.04957100	-1.68836400
H	2.63891500	-2.43768800	-2.48508400
H	1.83632400	-3.66610000	-1.48172600
C	4.16233500	3.55820200	0.40254000
H	5.01581300	3.70942200	1.07141300
H	4.45912100	3.94308600	-0.57908700
H	3.32373900	4.15342300	0.76832600
C	-3.44766300	-0.76974700	-0.64615400
H	-3.73562900	-1.77321400	-0.31571500
H	-3.61840400	-0.72037600	-1.72596900
C	-4.34982200	0.25730700	0.04892800
H	-4.17750300	0.21331300	1.13047600
H	-4.06230100	1.26391000	-0.27628500

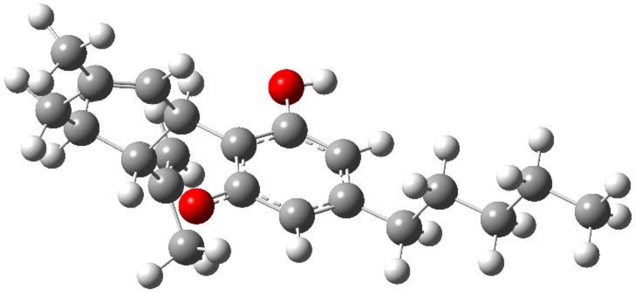

C	-5.83044100	0.02978900	-0.24073000
H	-6.11174200	-0.98042800	0.08116100
H	-5.99874000	0.06988400	-1.32387700
C	-6.73724500	1.04734800	0.44700500
H	-6.56648400	1.00734800	1.52828900
H	-6.45549200	2.05531800	0.12378800
C	-8.21445400	0.80637200	0.15065300
H	-8.52192000	-0.18659400	0.49060600
H	-8.85004300	1.54265600	0.64794100
H	-8.40967100	0.86553200	-0.92379000
C	2.81615900	-2.08737400	-0.37678600
C	3.16222800	-2.88429100	0.63565000
H	3.70190100	-2.50976800	1.50049300
H	2.90663000	-3.93944600	0.63256700
O	0.87608900	1.18202700	-1.81214300

cannabidiol anion C3′ (anion 1)

Charge=-1, Multiplicity=1

C	-1.21674500	0.17284600	-1.13129600
C	0.12131600	0.42071100	-0.81970900
C	0.73599100	-0.06721600	0.33336800
C	-0.04618300	-0.84768300	1.24816700
C	-1.40518800	-1.09374600	0.90433400
C	-1.98468700	-0.59996400	-0.25668800
H	-1.64249700	0.57360200	-2.04669400
H	-1.99821000	-1.69351900	1.59003700
O	0.45311000	-1.32374500	2.35343400
C	2.21077200	0.15170300	0.60938900
H	2.39281400	-0.30305200	1.58889800
C	3.12528800	-0.59755700	-0.38829900
H	2.91667600	-0.21058900	-1.39127400
C	2.60302100	1.61112900	0.70464500
C	3.78269900	2.10338300	0.31833300
H	1.85831800	2.28710500	1.12274100
C	4.85981900	1.21964400	-0.26473100
H	5.82286300	1.49315100	0.18034000
H	4.95167800	1.44283700	-1.33601300
C	4.58528000	-0.26937000	-0.05888800
H	5.25665100	-0.86653700	-0.68217200
H	4.78483200	-0.53913500	0.98397000
C	2.10084500	-2.60064200	-1.60888200
H	1.15630400	-2.06158000	-1.74438800
H	2.69843800	-2.42136700	-2.50885700

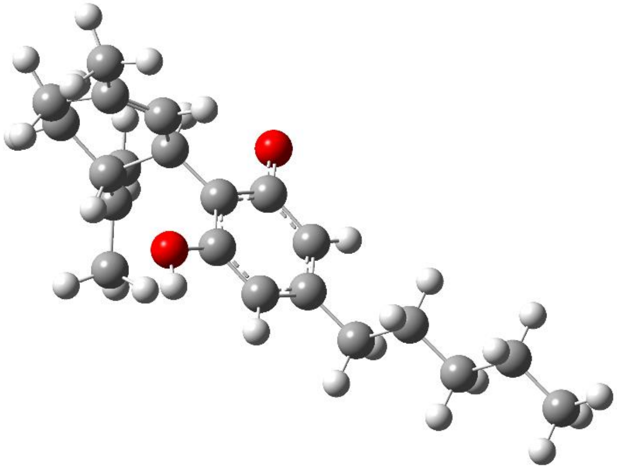

H	1.89033300	-3.66893700	-1.53368900
C	4.11899400	3.56496100	0.41456100
H	4.98264200	3.72192500	1.06887100
H	4.39329200	3.96013500	-0.56951100
H	3.28029100	4.14748400	0.80000000
C	-3.44720000	-0.83960000	-0.54531100
H	-3.74526500	-1.81519900	-0.14850500
H	-3.61034200	-0.86352600	-1.62757200
C	-4.34584600	0.24088500	0.06864400
H	-4.18615900	0.26518500	1.15276400
H	-4.04383600	1.22135100	-0.31757300
C	-5.82525700	0.00996500	-0.22448300
H	-6.11992700	-0.97689000	0.15301900
H	-5.98081700	-0.01243600	-1.31007900
C	-6.73018000	1.07414300	0.39140500
H	-6.57506900	1.09317600	1.47558000
H	-6.43279300	2.05940600	0.01626300
C	-8.20561300	0.83194800	0.08725100
H	-8.52811700	-0.13821300	0.47565600
H	-8.84033700	1.60006300	0.53501500
H	-8.38528100	0.83489600	-0.99153500
C	2.82021600	-2.08469200	-0.39376000
C	3.14695900	-2.88039100	0.62551300
H	3.65697600	-2.50049800	1.50598400
H	2.90405500	-3.93843800	0.61336200
O	0.89318900	1.16746600	-1.69062200
H	0.34629300	1.43709400	-2.43850900

2-nitrophenol Ref 32’s methodology

Charge=0, Multiplicity=1

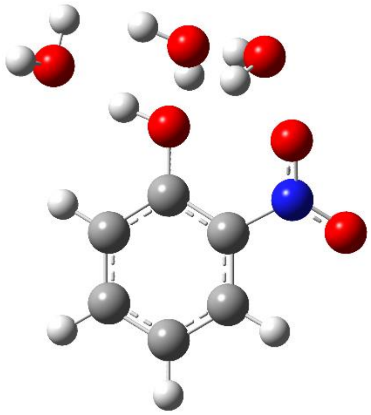

C	-1.91198000	2.37366200	0.01941500
C	-2.91753700	1.40086200	0.07082400
C	-2.56407300	0.06542600	0.08260300
C	-1.21303400	-0.30704100	0.06325800
C	-0.18827100	0.66470900	0.01202900
C	-0.57435600	2.01289800	-0.01402400
H	-2.17530600	3.42486700	0.00528200
H	-3.96102900	1.68646800	0.10389400
H	-3.31661400	-0.70963500	0.12589900
H	0.19885800	2.76988700	-0.06368000
O	1.11045300	0.30962300	-0.04156300
H	1.71106100	1.10213700	-0.13705700
N	-0.91899800	-1.72818100	0.08712700

O	0.18775600	-2.11035800	0.46885800
O	-1.80466000	-2.51316900	-0.26768200
O	2.86961600	2.29174000	-0.33436000
H	3.69655200	1.91122100	-0.66000600
H	3.10317700	2.71530400	0.50270100
O	2.44378800	-1.00126600	2.27088200
H	1.92101100	-0.70017100	1.50942400
H	3.03716700	-0.26533300	2.46348300
O	2.00675100	-1.40924800	-2.27109200
H	1.68428100	-0.88177200	-1.52164600
H	2.23971100	-0.75737800	-2.94315300

2-nitrophenol anion Ref 32's methodology

Charge=-1, Multiplicity=1

C	-1.76611200	2.44812200	0.06824600
C	-2.83693300	1.52919100	0.01658400
C	-2.55986700	0.18406100	-0.02097900
C	-1.22649200	-0.28337700	0.00733000
C	-0.10815200	0.62631300	0.05820000
C	-0.46237600	2.01345000	0.08487600
H	-1.97381500	3.51262800	0.09655000
H	-3.86222200	1.87696300	0.01101700
H	-3.36035600	-0.54141300	-0.05710000
H	0.35672500	2.72278200	0.12080300
O	1.13454500	0.28150900	0.06076200
N	-1.04211400	-1.69964900	-0.04226600
O	0.07148300	-2.19701400	0.18718200
O	-2.02064600	-2.42688200	-0.31341900
H	2.40966900	1.48866700	-0.14192500
O	3.18458400	2.08211100	-0.28192700
H	2.89744300	2.94073500	0.05027400
O	2.32376200	-0.99901500	2.22001300
H	1.87123400	-0.58354200	1.45471800
H	2.03760600	-1.91989400	2.19016900
O	2.29091300	-1.17263500	-2.01094200
H	1.85123300	-0.70225800	-1.27033500
H	2.94973800	-0.54827400	-2.33719400

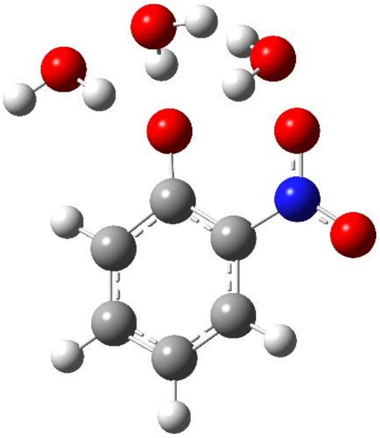

Cartesian coordinates of the optimized acids and conjugate bases of the antioxidants studied at the M06–2X(SMD)/6–311++G(d,p) level of theory in water at 298.15 K.

o-butylated hydroxyanisole

Charge=0, Multiplicity=1

C	-2.02076000	1.11864100	0.00002200
C	-1.85708200	-0.25874100	0.00000000
C	-0.57345200	-0.80936600	0.00000600
C	0.57913700	-0.02734200	0.00000000
C	0.39342600	1.37161800	-0.00000400
C	-0.88019600	1.92087600	0.00002200
H	-2.99807900	1.58184300	0.00004100
H	-0.50543800	-1.88947800	0.00001400
H	-0.98419400	3.00140800	0.00003900
O	1.49477100	2.19802900	-0.00003100
H	1.19654000	3.11575900	-0.00006500
O	-2.89310300	-1.16131500	-0.00005600
C	-4.21108800	-0.62826300	0.00001500
H	-4.88283300	-1.48403200	0.00002400
H	-4.38815700	-0.02408800	0.89369700
H	-4.38825400	-0.02405200	-0.89362200
C	1.97418200	-0.66387000	0.00000500
C	1.89885300	-2.19480800	0.00005400
H	1.38824500	-2.57697300	0.88773000
H	1.38823200	-2.57703000	-0.88759000
H	2.91564500	-2.59479700	0.00005800
C	2.74648100	-0.24170600	-1.26190800
H	2.89648100	0.83614100	-1.31068900
H	3.72785500	-0.72477000	-1.26503700
H	2.21003500	-0.55888200	-2.16068900
C	2.74651300	-0.24163000	1.26187200
H	3.72785800	-0.72475100	1.26504100
H	2.89657700	0.83621300	1.31055000
H	2.21005600	-0.55868900	2.16068700

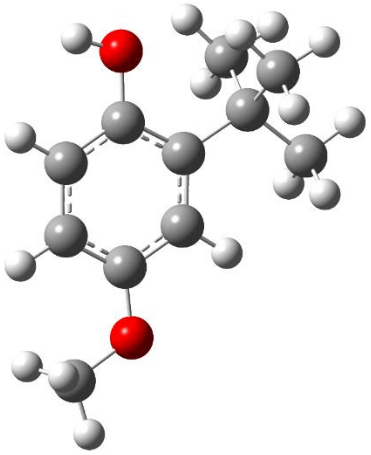

o-butylated hydroxyanisole anion

Charge=-1, Multiplicity=1

C	-1.95652600	1.41210000	-0.19376600
C	-1.89993100	0.03439900	-0.33104800
C	-0.67155700	-0.61744800	-0.26629800
C	0.53211500	0.06229700	-0.06830800
C	0.50020200	1.49642700	0.07482700
C	-0.77629000	2.11797300	0.00116000
H	-2.91304100	1.92331500	-0.24294300
H	-0.68266600	-1.69559500	-0.37951800
H	-0.80571700	3.19856200	0.10442200
O	1.56578000	2.22527400	0.26294200
O	-3.06620200	-0.70354900	-0.54914000
C	-3.63730800	-1.20005600	0.65997500
H	-4.53440300	-1.75646400	0.38916800
H	-2.93566400	-1.86514800	1.17232000
H	-3.90552600	-0.37329400	1.32464800
C	1.85950800	-0.70445100	-0.00201000
C	1.66949100	-2.21436900	-0.19320500
H	1.03132600	-2.64704300	0.58212300
H	1.23563700	-2.45012700	-1.16892500
H	2.64519500	-2.70412000	-0.13385600
C	2.81085200	-0.22658500	-1.11314400
H	3.04339200	0.83310000	-1.01711700
H	3.74689400	-0.79235900	-1.06842500
H	2.36171500	-0.39583600	-2.09652700
C	2.51535000	-0.50315300	1.37535800
H	3.47057800	-1.03642400	1.41507400
H	2.69846500	0.55013200	1.58428500
H	1.87176100	-0.90530200	2.16361000

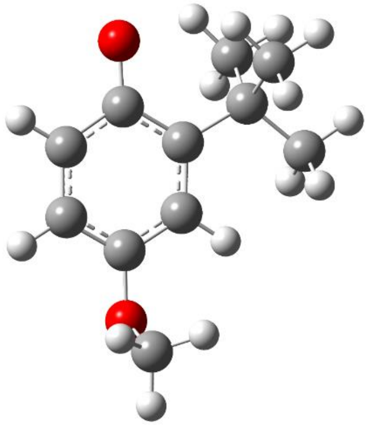

m-butylated hydroxyanisole

Charge=0, Multiplicity=1

C	-1.26939500	1.65578800	-0.00000900
C	-0.02163000	1.03597100	-0.00001200
C	0.08209100	-0.37467000	0.00002000
C	-1.11080400	-1.09816700	0.00004100
C	-2.35680800	-0.47398200	0.00004400
C	-2.44247100	0.90539000	0.00002100
H	-1.34749600	2.73419400	-0.00003600
H	-3.41054400	1.39193100	0.00002200
O	-3.53008800	-1.19937500	0.00007200
H	-3.32468300	-2.14199100	0.00007900

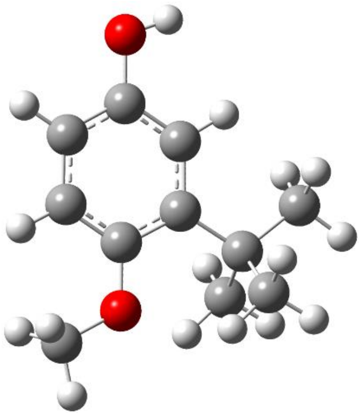

O	1.14346400	1.75700300	-0.00006500
C	1.05209500	3.17484300	-0.00011500
H	0.53745300	3.53705600	0.89384000
H	2.07764600	3.53854600	-0.00015400
H	0.53740700	3.53699000	-0.89406900
H	-1.09379600	-2.18134500	0.00006100
C	1.43902300	-1.09299900	0.00001200
C	1.27418200	-2.61773800	-0.00019000
H	0.74318200	-2.97062000	0.88769900
H	0.74332200	-2.97040000	-0.88825000
H	2.26642700	-3.07511200	-0.00017000
C	2.23701600	-0.72299400	-1.26282800
H	2.45667500	0.34236700	-1.31317000
H	3.18432600	-1.26989000	-1.26538400
H	1.68079100	-1.00635200	-2.16093000
C	2.23687200	-0.72332200	1.26304100
H	3.18416600	-1.27024500	1.26557800
H	2.45655500	0.34201900	1.31367300
H	1.68053000	-1.00688700	2.16100500

m-butylated hydroxyanisole anion

Charge=-1, Multiplicity=1

C	-1.40955100	1.54084400	0.00004800
C	-0.12049300	1.01643600	0.00006100
C	0.06975700	-0.38535100	-0.00003700
C	-1.07213400	-1.18506300	-0.00013100
C	-2.40462100	-0.68962400	-0.00014800
C	-2.53222700	0.71242000	-0.00005800
H	-1.56095600	2.61298600	0.00012400
H	-3.52406600	1.15377900	-0.00006500
O	-3.43792100	-1.50372900	-0.00024600
O	1.00298800	1.82460600	0.00018600
C	0.80109000	3.22698300	0.00029600
H	0.25838500	3.55150500	0.89320200
H	1.79393900	3.67409000	0.00038300
H	0.25847300	3.55165800	-0.89260700
H	-0.97013100	-2.26359100	-0.00020300
C	1.47333300	-1.01313600	-0.00002300
C	1.41715900	-2.54614100	0.00013700
H	0.91139000	-2.93572900	0.88719400
H	0.91130600	-2.93590900	-0.88679400
H	2.43924500	-2.93342800	0.00012800
C	2.24672400	-0.59231700	-1.26228800

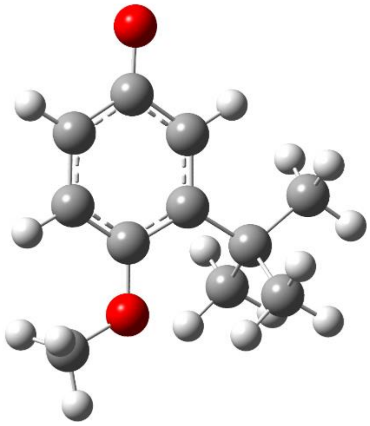

H	2.39088100	0.48587400	-1.31554000
H	3.23058100	-1.07129700	-1.26543000
H	1.71181100	-0.91526400	-2.16028500
C	2.24680200	-0.59205100	1.26210500
H	3.23067000	-1.07101000	1.26527600
H	2.39094100	0.48615600	1.31512700
H	1.71195700	-0.91482600	2.16020400

tocol

Charge=0, Multiplicity=1

C	0.12301300	-0.63979000	-0.15500500
C	0.47708800	0.71189500	-0.09792700
C	2.80704000	0.05733500	0.03063700
O	4.12318500	0.45925500	0.11964700
H	4.69711300	-0.31663200	0.11077400
C	-0.58256500	1.78507500	-0.13757700
H	-0.70773800	2.20535300	0.86564800
H	-0.24916800	2.60302900	-0.77976300
C	-1.90494000	1.21376600	-0.63840600
H	-1.85092600	1.03463500	-1.71680400
H	-2.72364900	1.91293100	-0.45583000
C	-2.22557300	-0.11605400	0.04282800
C	-3.49655200	-0.72299900	-0.52272900
H	-3.67012000	-1.71147300	-0.09124000
H	-4.34886500	-0.08438800	-0.28163400
H	-3.42512600	-0.81751800	-1.60860200
C	-2.31151300	0.01177500	1.56022900
H	-3.06092600	0.76059200	1.82749800
H	-2.60297400	-0.94595000	1.99709300
H	-1.35533200	0.31163600	1.99476200
O	-1.17920600	-1.06933800	-0.28142000
C	1.10451500	-1.62859800	-0.12051200
H	0.80322000	-2.66876100	-0.16810200
C	2.44657200	-1.28675500	-0.03013900
H	3.21235000	-2.05485000	-0.00543500
C	1.83029400	1.04208500	-0.00328500
H	2.12603100	2.08565100	0.03712700

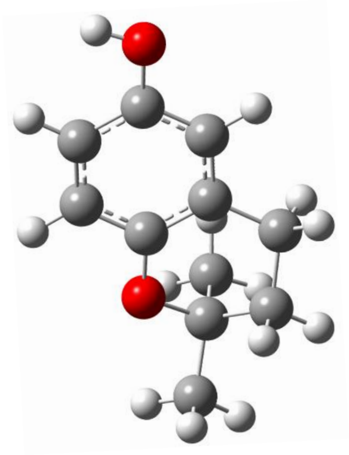

tocol anion

Charge=-1, Multiplicity=1

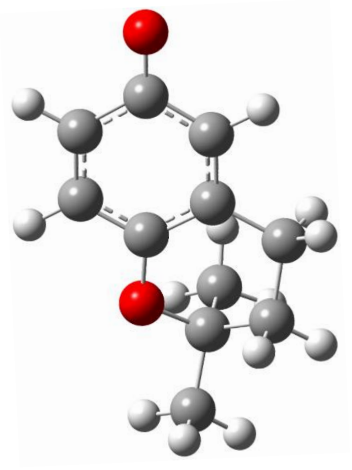

C	0.16438300	-0.63849900	-0.17869300
C	0.52621500	0.70827700	-0.11642700
C	2.91320200	0.07330900	0.04244200
O	4.18098700	0.40081400	0.14795400
C	-0.53532300	1.78378800	-0.14489500
H	-0.64631400	2.20659200	0.85938300
H	-0.21247700	2.60254100	-0.79213100
C	-1.87026100	1.22026400	-0.62358500
H	-1.83991300	1.05160700	-1.70484700
H	-2.68389200	1.91965800	-0.41802800
C	-2.17985200	-0.11858400	0.04782200
C	-3.47173400	-0.70773700	-0.49073600
H	-3.64135100	-1.70094600	-0.06817200
H	-4.31328200	-0.06700200	-0.21899900
H	-3.43063400	-0.78906400	-1.57936300
C	-2.23056400	-0.00685500	1.56916800
H	-2.97242100	0.73944600	1.86364000
H	-2.51320000	-0.96917400	2.00228300
H	-1.26390300	0.28673000	1.98392600
O	-1.15079500	-1.06752800	-0.31960700
C	1.15019600	-1.62284900	-0.13474300
H	0.84906400	-2.66460600	-0.18603500
C	2.49106600	-1.28139700	-0.03161400
H	3.24593400	-2.06107600	-0.00082300
C	1.88012900	1.03854900	-0.00692800
H	2.15611700	2.08940000	0.04151600

δ-tocopherol

Charge=0, Multiplicity=1

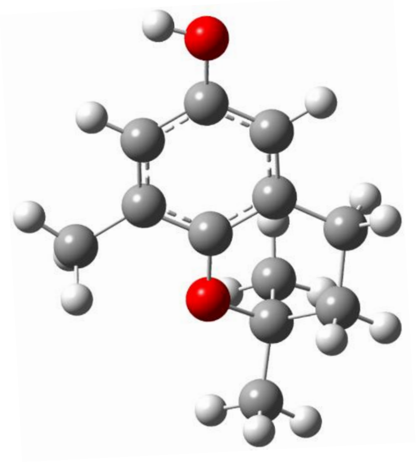

C	-0.09658900	0.38709300	-0.12317600
C	-0.38532700	-0.97899700	-0.10959400
C	-2.73599800	-0.44529300	0.04021400
O	-4.03565000	-0.90288800	0.11722200
H	-4.63949900	-0.15011900	0.12121500
C	0.72461900	-1.99861600	-0.18774700
H	0.87547800	-2.44521100	0.80052600
H	0.42617900	-2.81041300	-0.85455700
C	2.01600900	-1.35133000	-0.67531300
H	1.94692500	-1.13740800	-1.74653400
H	2.86729500	-2.01771400	-0.52095000
C	2.27720700	-0.03284300	0.05102800

C	3.51377900	0.65490000	-0.49816900
H	3.64234400	1.63434400	-0.03169000
H	4.39740300	0.04990300	-0.28443600
H	3.43087700	0.78507400	-1.57955000
C	2.38001100	-0.21030800	1.56255600
H	3.16362800	-0.93415600	1.79892500
H	2.63230100	0.74353300	2.03121700
H	1.44084600	-0.56689500	1.99122300
O	1.18459100	0.87936800	-0.23206400
C	-1.11770000	1.34699000	-0.06128500
C	-2.43732700	0.91541200	0.02042200
H	-3.24245100	1.64270200	0.06828900
C	-0.77401600	2.80985400	-0.08883600
H	-0.11234300	3.07110000	0.74097000
H	-0.24948000	3.07172800	-1.01144200
H	-1.67708300	3.41632600	-0.01927800
C	-1.71973100	-1.38405500	-0.02460900
H	-1.96355700	-2.44146700	-0.01814800

δ-tocopherol anion

Charge=-1, Multiplicity=1

C	-0.13564500	0.38426600	-0.14384300
C	-0.42428600	-0.97977000	-0.12883600
C	-2.83445600	-0.48493100	0.05170700
O	-4.08544300	-0.88026300	0.14713100
C	0.69260400	-1.99596900	-0.20049300
H	0.83223600	-2.44907900	0.78689300
H	0.40795400	-2.80650000	-0.87557200
C	1.99326700	-1.34827200	-0.66463300
H	1.94658700	-1.14020900	-1.73850700
H	2.84334000	-2.01203700	-0.49016900
C	2.23609000	-0.02218700	0.05686700
C	3.49082000	0.65605500	-0.46422900
H	3.61161400	1.63857800	-0.00164800
H	4.36696100	0.04981100	-0.22425600
H	3.43695600	0.77984200	-1.54834700
C	2.30474000	-0.19036000	1.57248600
H	3.08571400	-0.90900600	1.83294100
H	2.54211400	0.76764300	2.04103800
H	1.35732300	-0.54820200	1.98107500
O	1.15601800	0.88406100	-0.26608900
C	-1.16717600	1.33144900	-0.06970900
C	-2.48287400	0.89147300	0.02274900

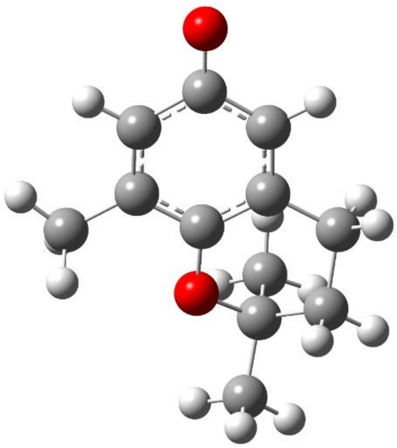

H	-3.28129100	1.62696600	0.07833400
C	-0.83484700	2.79993900	-0.09625600
H	-0.17064100	3.06880400	0.72969200
H	-0.31918700	3.07319600	-1.02099300
H	-1.74266000	3.39922600	-0.01870800
C	-1.75631300	-1.39342700	-0.02948200
H	-1.97316100	-2.45901900	-0.01807600

β-tocopherol

Charge=0, Multiplicity=1

C	0.14376600	0.58871600	-0.14516100
C	-0.35182700	-0.71680800	-0.13538400
C	-2.58324400	0.16713300	0.04235000
O	-3.94386900	-0.05768700	0.13686200
H	-4.40035200	0.78990700	0.20174200
C	0.59510800	-1.88880900	-0.23826300
H	0.64860000	-2.39798800	0.73083500
H	0.19655600	-2.62081300	-0.94505100
C	1.98416500	-1.44157400	-0.68042400
H	1.98017500	-1.21170200	-1.75050400
H	2.71677900	-2.23383500	-0.51213300
C	2.42247400	-0.18412600	0.06467300
C	3.76437100	0.31242500	-0.44150400
H	4.02669200	1.25683900	0.04104200
H	4.53967900	-0.42158800	-0.21220200
H	3.73328200	0.46332100	-1.52290400
C	2.45173600	-0.38271000	1.57689100
H	3.12232200	-1.20768300	1.82900500
H	2.81639100	0.52491000	2.06320400
H	1.46030100	-0.61205000	1.97340500
O	1.48723500	0.87857800	-0.24749200
C	-0.71090500	1.69648500	-0.07737000
C	-2.07725700	1.46454800	0.01063000
H	-2.76349100	2.30498600	0.06183400
C	-1.73665300	-0.93669100	-0.02269600
C	-2.28199600	-2.33981400	0.01170000
H	-2.21313000	-2.81227200	-0.97323500
H	-1.71446500	-2.96134600	0.70801000
H	-3.32655900	-2.35074700	0.31721500
C	-0.14860600	3.09031400	-0.09996400
H	0.54668500	3.24584300	0.72896600
H	0.40668900	3.27675400	-1.02295700
H	-0.94987100	3.82580100	-0.02409600

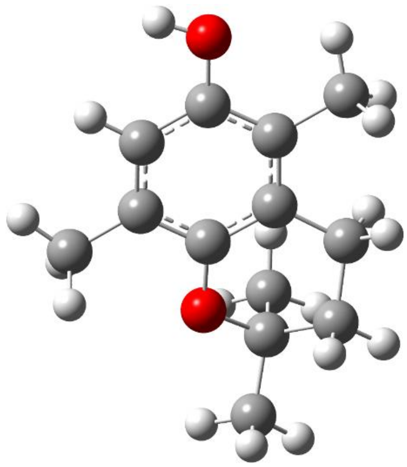

β-tocopherol anion

Charge=-1, Multiplicity=1

C	0.11250400	0.59395100	-0.15307300
C	-0.39819100	-0.70635800	-0.13891700
C	-2.68373100	0.18048300	0.04916800
O	-3.98836900	0.02042400	0.13407700
C	0.54615800	-1.88474700	-0.24047200
H	0.59448200	-2.39597800	0.72821400
H	0.14819300	-2.61640800	-0.94835600
C	1.94293000	-1.45246000	-0.67469900
H	1.95011200	-1.23013100	-1.74667700
H	2.66778600	-2.25025300	-0.49607100
C	2.38622000	-0.19083300	0.06213300
C	3.74268000	0.27982300	-0.43216600
H	4.01326000	1.22602100	0.04265600
H	4.50522400	-0.46221900	-0.18583300
H	3.72744300	0.41954600	-1.51558200
C	2.40015700	-0.37787300	1.57701000
H	3.05969700	-1.20740600	1.84358400
H	2.76984200	0.53008200	2.05944500
H	1.40256400	-0.59305300	1.96559100
O	1.47016100	0.87411000	-0.27569400
C	-0.73950500	1.69983100	-0.07243300
C	-2.10838400	1.47561800	0.02151200
H	-2.77893200	2.32945400	0.07734700
C	-1.78230700	-0.91802400	-0.01671000
C	-2.32302300	-2.32491900	0.02733100
H	-2.38755200	-2.76962100	-0.97290500
H	-1.69178700	-2.98231500	0.62962100
H	-3.32650400	-2.33458700	0.45167500
C	-0.17051700	3.09420600	-0.09281900
H	0.53665100	3.24557700	0.72733600
H	0.37307800	3.28993800	-1.02140900
H	-0.96783500	3.83289000	-0.00088900

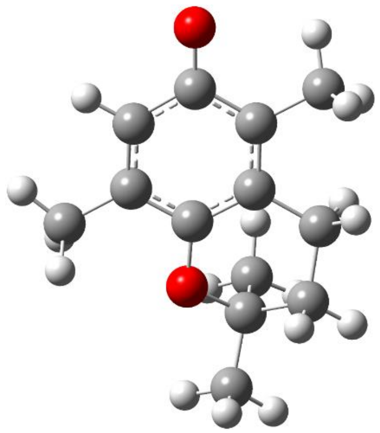

γ-tocopherol

Charge=0, Multiplicity=1

C	0.13295500	0.26812200	-0.13173000
C	0.03234400	-1.12392800	-0.10873500
C	-2.37277600	-0.89780400	0.02928800
O	-3.63299100	-1.45913100	0.11055500
H	-3.55336500	-2.42049500	0.08950700
C	1.26383500	-1.99472800	-0.17356000
H	1.46470700	-2.41102800	0.81913000
H	1.07577400	-2.84291100	-0.83545300
C	2.46598300	-1.19267400	-0.65828500
H	2.38247700	-1.00335600	-1.73310500
H	3.39505600	-1.74021700	-0.48563600
C	2.54210500	0.15820200	0.05098400
C	3.68424400	0.99522100	-0.49575200
H	3.67889200	1.98874300	-0.04147800
H	4.63703800	0.51334700	-0.26655600
H	3.59640600	1.10040200	-1.57948700
C	2.65020100	0.01640800	1.56596000
H	3.52168000	-0.59145400	1.82079200
H	2.76612100	1.00182400	2.02283700
H	1.76333600	-0.45823900	1.99136000
O	1.34428400	0.91400400	-0.25990100
C	-1.00800900	1.08357600	-0.06733300
C	-2.27873000	0.49786500	-0.00416700
C	-1.23771700	-1.69202200	-0.02059200
C	-0.86715400	2.58307100	-0.08857400
H	0.12387900	2.89026800	0.24048100
H	-1.01988900	2.97651300	-1.09915200
H	-1.60775100	3.05363600	0.55953600
C	-3.51787900	1.35393100	0.03047800
H	-3.63483500	1.83892800	1.00466000
H	-3.46473000	2.14481000	-0.72060600
H	-4.41174300	0.76263100	-0.15875200
H	-1.33981800	-2.77382100	-0.00119300

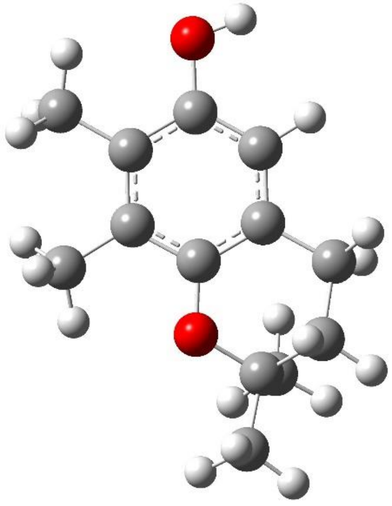

γ-tocopherol anion

Charge=-1, Multiplicity=1

C	0.10023400	0.25576900	-0.15111800
C	0.01489400	-1.13528200	-0.12869000
C	-2.44202400	-0.98457100	0.04189400
O	-3.61323700	-1.57460600	0.15113500
C	1.26277400	-1.98729900	-0.18746800
H	1.45965800	-2.40895900	0.80426000
H	1.09708100	-2.83504100	-0.85664200
C	2.46384400	-1.17007700	-0.65095300
H	2.39776100	-0.98172000	-1.72747500
H	3.39749200	-1.70541600	-0.46254800
C	2.50818400	0.18437000	0.05634200
C	3.65774800	1.02933000	-0.46432900
H	3.63334100	2.02412800	-0.01299400
H	4.60958500	0.55694200	-0.21203900
H	3.59529500	1.13242800	-1.55018400
C	2.58869500	0.04374900	1.57416300
H	3.46245300	-0.55283900	1.84775100
H	2.68325800	1.03074000	2.03314300
H	1.69925200	-0.44159400	1.98134800
O	1.31455300	0.92299500	-0.28699300
C	-1.05848600	1.04702900	-0.08044500
C	-2.31668900	0.43774700	-0.01349900
C	-1.24481900	-1.72713700	-0.02718400
C	-0.93992800	2.55276300	-0.08381600
H	0.06714400	2.87105200	0.17967500
H	-1.17292800	2.96861200	-1.06983000
H	-1.63662100	2.99977100	0.62771500
C	-3.57234600	1.27178100	0.01793200
H	-3.85404500	1.54830600	1.04115500
H	-3.46045100	2.19697800	-0.54939800
H	-4.40869900	0.71280500	-0.40306500
H	-1.31259500	-2.81233400	-0.00098600

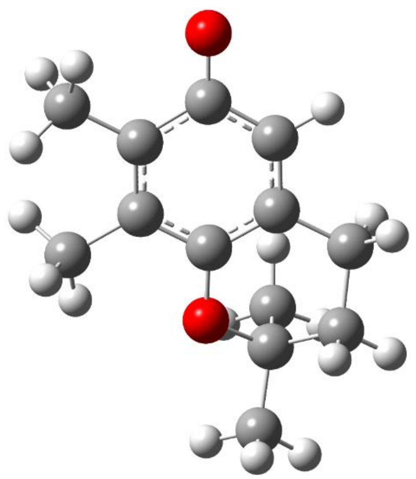

α-tocopherol

Charge=0, Multiplicity=1

C	-0.27300900	-0.47007000	-0.15644700
C	-0.04239700	0.90784100	-0.13718600
C	2.31548300	0.46360100	0.02913200
O	3.60144200	0.96872900	0.11050300
H	4.23775000	0.24685400	0.17148300
C	-1.19240600	1.88346000	-0.22861900
H	-1.33399500	2.36887500	0.74393100
H	-0.94119400	2.68105100	-0.93217700
C	-2.47791900	1.19127100	-0.66489300
H	-2.44424000	0.97844100	-1.73796700
H	-3.34405500	1.82982100	-0.47783200
C	-2.65916700	-0.13461000	0.06720200
C	-3.88863700	-0.87172300	-0.43224600
H	-3.96165500	-1.85387400	0.04065300
H	-4.78636900	-0.30057400	-0.18645600
H	-3.84242900	-1.00267600	-1.51574700
C	-2.70933000	0.03660900	1.58241500
H	-3.52456000	0.71244500	1.85165500
H	-2.88578500	-0.93033900	2.05909000
H	-1.77710100	0.44911600	1.97410400
O	-1.54501300	-0.99533000	-0.27177500
C	0.77996200	-1.39546600	-0.09047200
C	2.09283000	-0.91807500	-0.00444400
C	1.27234900	1.38393700	-0.02371100
C	1.54237900	2.86516000	0.02142900
H	1.41008800	3.31894000	-0.96600000
H	0.85034500	3.36597700	0.70185800
H	2.55778500	3.07342900	0.35256100
C	0.49854200	-2.87776900	-0.11902100
H	-0.56996800	-3.07580100	-0.14035500
H	0.94748600	-3.34505000	-0.99962700
H	0.91845300	-3.37243000	0.76012800
C	3.28092100	-1.84456200	0.05406400
H	3.85830600	-1.68804200	0.97150600
H	2.98647400	-2.89053800	0.02701000
H	3.95561500	-1.67044400	-0.79086600

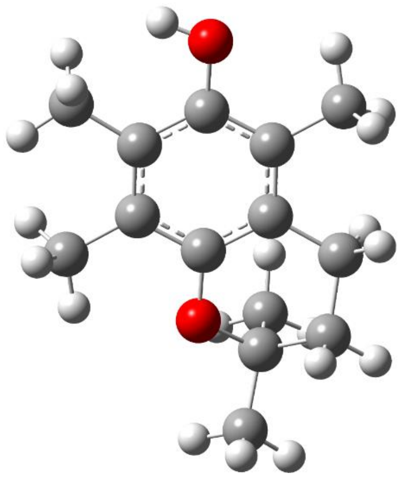

α-tocopherol anion

Charge=-1, Multiplicity=1

C	-0.24331300	-0.46118700	-0.16562100
C	-0.00820100	0.91741200	-0.14570700
C	2.40433200	0.48749200	0.02803800
O	3.64370000	0.92622200	0.10328000
C	-1.16983400	1.88692000	-0.24312200
H	-1.31234900	2.38370700	0.72348300
H	-0.93245300	2.67740500	-0.95874900
C	-2.45960100	1.19199200	-0.66441800
H	-2.43835900	0.97902600	-1.73812500
H	-3.32423400	1.83093700	-0.46909200
C	-2.63060300	-0.13647900	0.06560700
C	-3.87231700	-0.86669600	-0.41599500
H	-3.94239800	-1.85041900	0.05449000
H	-4.76461800	-0.29334700	-0.15542200
H	-3.84365200	-0.99507500	-1.50056400
C	-2.66182000	0.03261300	1.58254400
H	-3.47561000	0.70503600	1.86498700
H	-2.82880200	-0.93578000	2.06025200
H	-1.72563900	0.44735500	1.96184000
O	-1.52664300	-0.99069700	-0.29910300
C	0.81129400	-1.38262400	-0.08648200
C	2.12587500	-0.90818800	-0.00547500
C	1.30773400	1.38777200	-0.02013400
C	1.61227500	2.86271900	0.05411600
H	2.04412300	3.23306700	-0.88312500
H	0.72894900	3.46129200	0.27274700
H	2.35333200	3.05843400	0.83324700
C	0.52228500	-2.86692900	-0.09939600
H	-0.54654200	-3.06272800	-0.05523100
H	0.91270500	-3.33993600	-1.00539400
H	0.99278700	-3.36586100	0.75113200
C	3.30715800	-1.84284400	0.05110000
H	3.84936900	-1.73621900	0.99687500
H	3.02095200	-2.88770000	-0.05668600
H	4.02439600	-1.60523000	-0.74041500

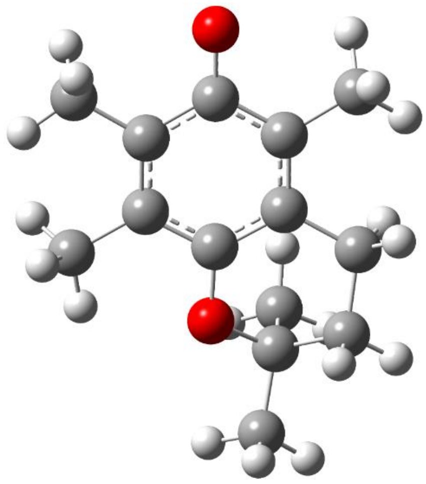

N,N-dimethyl-4-aminophenol

Charge=0, Multiplicity=1

C	2.18372300	-0.00499200	0.03390900
C	1.51592500	1.19887600	-0.17936900
C	0.13470400	1.21725400	-0.28541400
C	-0.62680800	0.04037200	-0.17503500
C	0.06451800	-1.15666000	0.04348600
C	1.45677000	-1.17984600	0.14021900
H	2.08649800	2.11607900	-0.27057600
H	-0.35584100	2.16357900	-0.47654200
H	-0.46472000	-2.09451800	0.14088300
H	1.97396200	-2.11947600	0.30639900
N	-2.03676000	0.09621200	-0.32790400
O	3.55848100	0.02866100	0.12846300
H	3.89125600	-0.86572700	0.27290600
C	-2.67825300	1.11459300	0.50986600
H	-3.74336800	1.13941200	0.27966100
H	-2.55578600	0.88846000	1.57865200
H	-2.27247200	2.10401700	0.31294400
C	-2.72188800	-1.18025400	-0.16736400
H	-3.78509800	-1.02610800	-0.34924000
H	-2.35610500	-1.90728900	-0.89247500
H	-2.60099200	-1.59725200	0.84322200

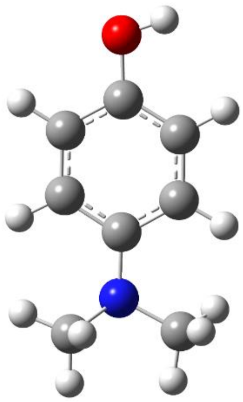

N,N-dimethyl-4-aminophenol anion

Charge=-1, Multiplicity=1

C	2.29270800	0.04181900	0.05155400
C	1.59249400	-1.16191500	-0.23503000
C	0.21214000	-1.20195200	-0.35740500
C	-0.57883300	-0.05471200	-0.19190900
C	0.08869200	1.14087400	0.08730500
C	1.47959800	1.18924300	0.19630700
H	2.16944100	-2.07219000	-0.37020100
H	-0.26773600	-2.14588900	-0.59740800
H	-0.46562400	2.06239500	0.21883300
H	1.96311800	2.13894000	0.40614700
N	-2.00305400	-0.15703200	-0.33955500
O	3.59899300	0.08007500	0.16760400
C	-2.70088100	1.12041100	-0.28283500
H	-3.76027000	0.94829300	-0.47645800
H	-2.61014900	1.60945000	0.69912300
H	-2.31873300	1.79951900	-1.04576500
C	-2.59031000	-1.06812100	0.64784600
H	-3.65730600	-1.17756600	0.44691400
H	-2.13174200	-2.05349900	0.58789900
H	-2.46521500	-0.68471600	1.67197400

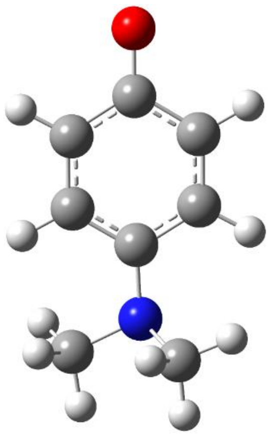

6-hydroxy-5,7,8-trimethyl-1,2,3,4-tetrahydroqunioline

Charge=0, Multiplicity=1

C	-1.74877100	-0.67365100	0.04457800
C	-0.54926800	-1.39014200	-0.02820700
C	0.67792100	-0.69282200	-0.08919800
C	0.70407000	0.70821000	-0.07013900
C	-0.50136000	1.42775700	-0.08293400
C	-1.70079500	0.72665300	-0.02417400
C	-0.49822800	2.93469500	-0.12424200
H	-0.33727700	3.35921000	0.87233600
H	0.29950600	3.30663000	-0.76919200
H	-1.44552300	3.31949300	-0.49838800
C	-0.50140500	-2.89676800	-0.04107000
H	0.17643300	-3.24421500	-0.82415600
H	-0.11958300	-3.29384500	0.90653700
H	-1.47419600	-3.34577200	-0.22328800
C	-3.10088400	-1.33024700	0.18997700
H	-3.67547900	-1.28232300	-0.74157100
H	-3.02570800	-2.37475200	0.47970300
H	-3.69030800	-0.83112400	0.96492100
O	-2.86838700	1.47539300	-0.00141700
H	-3.63057300	0.89740100	-0.11815300
C	2.02145000	1.45112500	0.02105900
H	2.30822900	1.84054200	-0.96336500
H	1.89991100	2.31874700	0.67375900
C	3.12755800	0.54469800	0.54940500
H	2.94378700	0.30187600	1.60117600
H	4.09641600	1.04403500	0.48293400
C	3.13869600	-0.73409400	-0.27209400
H	3.92032000	-1.41871400	0.05700500
H	3.34810100	-0.47724400	-1.31660300
N	1.86453700	-1.44938800	-0.20685400
H	1.88738400	-2.22986100	0.43789400

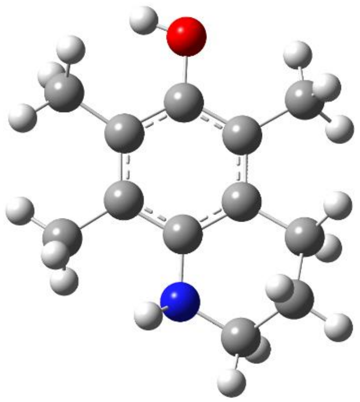

6-hydroxy-5,7,8-trimethyl-1,2,3,4-tetrahydroqunioline anion

Charge=-1, Multiplicity=1

C	-1.80123300	-0.58340600	0.03910300
C	-0.62960300	-1.34406700	-0.03284000
C	0.62785200	-0.71053400	-0.07165100
C	0.69924800	0.69077100	-0.04903900
C	-0.47838900	1.45554600	-0.07402100
C	-1.75654300	0.84028900	-0.01854300
C	-0.42319500	2.96245100	-0.13302700
H	-0.50990200	3.41712300	0.86165200
H	0.50467800	3.32304500	-0.57792800
H	-1.25431200	3.34754700	-0.72663700
C	-0.69873500	-2.85355200	-0.04462300

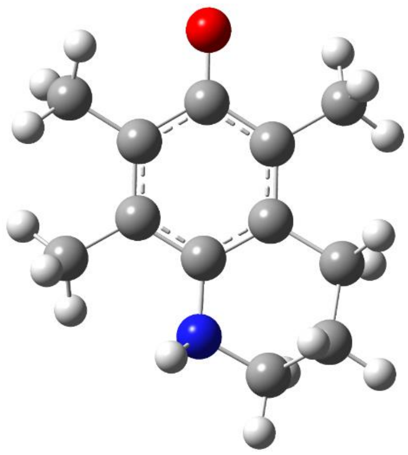

H	0.15645600	-3.27675200	-0.57130700
H	-0.69146300	-3.26735800	0.97069400
H	-1.60651800	-3.20827800	-0.53343400
C	-3.14869200	-1.25109900	0.16823100
H	-3.58486800	-1.49451500	-0.80828000
H	-3.08925600	-2.17957600	0.73793800
H	-3.85182500	-0.58923300	0.67420300
O	-2.86133900	1.56106200	-0.00687700
C	2.04510800	1.38985100	0.05322200
H	2.32285500	1.81958800	-0.91726100
H	1.96158200	2.23013100	0.74694300
C	3.14541500	0.44006300	0.51228500
H	3.00751800	0.18144100	1.56796900
H	4.12599200	0.91101900	0.40879300
C	3.06829500	-0.82630700	-0.32547000
H	3.87230300	-1.52227100	-0.08233600
H	3.17898900	-0.55577100	-1.38226800
N	1.80030000	-1.53210700	-0.15647900
H	1.84921600	-2.15993100	0.63987800

9-hydroxyjulolidine

Charge=0, Multiplicity=1

C	-0.20475700	2.55223000	0.02377800
H	-0.11457000	2.95305700	1.03934100
H	0.32276700	3.24788900	-0.63314700
C	-1.67964800	2.45985400	-0.34980500
H	-1.79414100	2.36002000	-1.43291200
H	-2.20682500	3.36521800	-0.04309100
C	-2.29680300	1.24758000	0.32466800
H	-3.36285400	1.17989100	0.10100000
H	-2.18911600	1.33374000	1.41867500
C	-2.33658100	-1.18101700	0.32333500
H	-2.23338500	-1.27116200	1.41745500
H	-3.39963400	-1.07885700	0.09816500
C	-1.75775400	-2.41218600	-0.35073300
H	-1.86687800	-2.30830900	-1.43402400
H	-2.31420800	-3.30032400	-0.04538800
C	-0.28748100	-2.55188200	0.02591700
H	0.21845500	-3.26644600	-0.62762000
H	-0.21294300	-2.95226200	1.04293600
N	-1.66476200	0.02289700	-0.15813700
O	3.93173500	-0.12174900	0.04176000
H	4.30077900	0.76971500	0.04232500
C	1.87553100	1.16474700	0.01201200
H	2.43673000	2.09519200	0.03008000
C	1.83602400	-1.23343600	0.01147400
H	2.36926500	-2.17878300	0.02950400
C	0.44370900	-1.22619400	-0.01562200
C	-0.25241400	0.00058700	-0.04772100

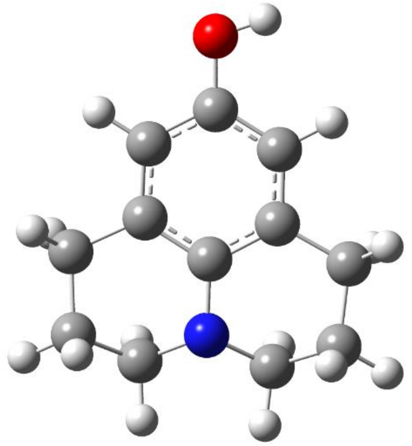

C	0.48185900	1.20300300	-0.01580900
C	2.55265000	-0.04576400	0.02010300

9-hydroxyjulolidine anion

Charge=-1, Multiplicity=1

C	0.20477000	2.54907100	0.01649800
H	-0.30137800	3.25324900	-0.64873500
H	0.10724600	2.95577500	1.02936800
C	1.68471900	2.43830100	-0.33503500
H	2.22121300	3.33264700	-0.01114200
H	1.81545100	2.34882700	-1.41752600
C	2.27414900	1.20832400	0.33297500
H	2.13716500	1.27850700	1.42626100
H	3.34614300	1.13642000	0.13799900
C	2.27413800	-1.20832700	0.33299600
H	3.34613600	-1.13642900	0.13803800
H	2.13713200	-1.27850000	1.42627900
C	1.68471700	-2.43830500	-0.33502200
H	2.22120500	-3.33265200	-0.01111900
H	1.81547000	-2.34882900	-1.41750900
C	0.20476200	-2.54907400	0.01648300
H	0.10721700	-2.95579300	1.02934400
H	-0.30137800	-3.25323700	-0.64877200
N	1.64324000	-0.00000300	-0.18806700
O	-3.97321500	0.00000300	0.06091000
C	-1.89796100	1.19315000	0.00948000
H	-2.42954600	2.14197100	0.03500200
C	-1.89796200	-1.19314500	0.00947600
H	-2.42955000	-2.14196600	0.03499400
C	-0.50475700	-1.20894400	-0.02766300
C	0.21658800	0.00000000	-0.06259900
C	-0.50475500	1.20894600	-0.02765600
C	-2.65665600	0.00000300	0.02451700

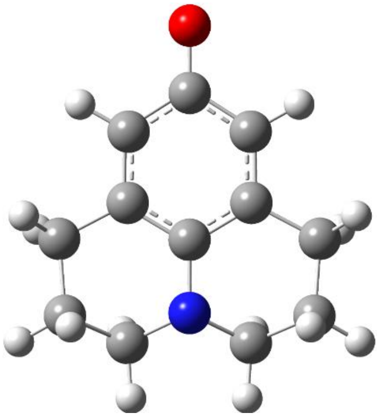

4-butadienylphenol

Charge=0, Multiplicity=1

C	2.32462300	1.13917100	-0.00006900
C	2.77711400	-0.17666400	-0.00002700
C	1.86394500	-1.23164000	0.00005700
C	0.50329600	-0.96623900	0.00009900
C	0.02054200	0.35033800	0.00004900
C	0.95943600	1.39062100	-0.00003000
H	3.04320900	1.95033100	-0.00012700
H	2.22820400	-2.25359300	0.00010000
H	-0.18799700	-1.80103100	0.00017900
H	0.60931200	2.41754900	-0.00005800
O	4.13149200	-0.38595900	-0.00007000
H	4.31982800	-1.33326700	0.00009000
C	-1.40828900	0.68290100	0.00008000

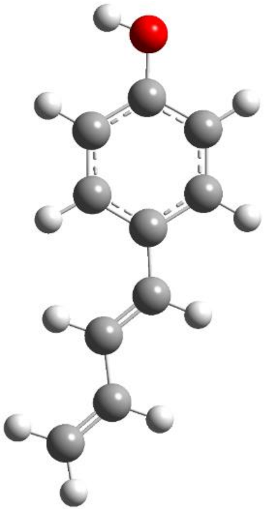

H	-1.63464000	1.74784100	0.00020200
C	-2.43774700	-0.17924100	-0.00004800
H	-2.26786100	-1.25358800	-0.00020200
C	-3.82632700	0.25913000	0.00000300
H	-3.99507000	1.33409000	0.00010700
C	-4.86901500	-0.57771300	-0.00004900
H	-4.72376300	-1.65399400	-0.00014300
H	-5.88862500	-0.21065000	0.00001600

4-butadienylphenol anion

Charge=-1, Multiplicity=1

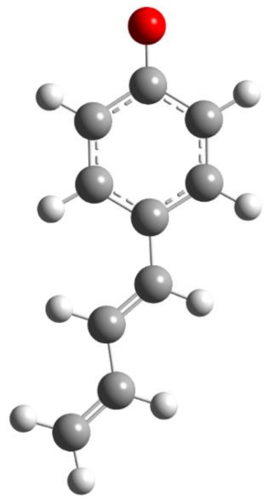

C	-2.38401900	1.11485100	-0.00002400
C	-2.89902100	-0.21236800	-0.00001700
C	-1.92171200	-1.25417800	0.00004800
C	-0.56628500	-0.98818500	0.00007800
C	-0.07198700	0.33034900	0.00005300
C	-1.02234800	1.36534000	0.00001100
H	-3.09093900	1.93881600	-0.00006000
H	-2.27467800	-2.28100700	0.00007100
H	0.12888400	-1.82226200	0.00012700
H	-0.67157700	2.39410500	0.00000000
O	-4.17049600	-0.46407800	-0.00006300
C	1.34869600	0.66094400	0.00005600
H	1.57156000	1.72794000	0.00011700
C	2.39477700	-0.18801000	-0.00002000
H	2.23948000	-1.26480800	-0.00009500
C	3.77363500	0.27373100	-0.00001200
H	3.91853700	1.35288700	0.00005900
C	4.84206000	-0.53308800	-0.00008700
H	4.72956600	-1.61340500	-0.00015800
H	5.85036400	-0.13595500	-0.00007900

4-vinylphenylphenol

Charge=0, Multiplicity=1

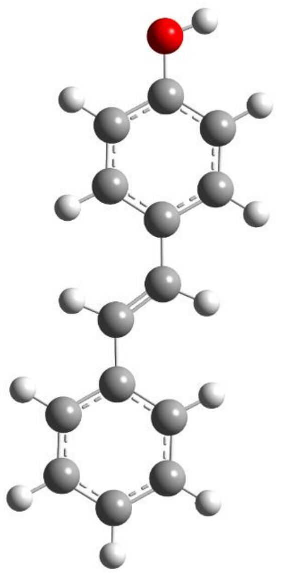

C	-2.32520100	1.32999100	-0.00003000
C	-1.45564800	0.23286600	-0.00019300
C	-2.02365800	-1.05133200	-0.00027900
C	-3.39723400	-1.22823200	-0.00013200
C	-4.24110700	-0.11656000	0.00010100
C	-3.70547000	1.16687100	0.00009800
H	-1.91111900	2.33292200	0.00002000
H	-1.38925400	-1.93006300	-0.00040800
H	-3.83070700	-2.22155400	-0.00014100
H	-4.36644100	2.02708600	0.00024300
C	-0.00821900	0.48083600	-0.00017300
H	0.26375700	1.53329100	0.00004600
C	0.95724500	-0.44955100	-0.00032500
H	0.68768100	-1.50255100	-0.00051800
C	2.40543400	-0.19377500	-0.00016200

C	2.96341900	1.09430900	-0.00020600
C	3.27640500	-1.29174100	0.00007900
C	4.34076800	1.26964000	0.00002400
H	2.32207700	1.96800000	-0.00042300
C	4.65664000	-1.11576200	0.00030800
H	2.85981900	-2.29380100	0.00010000
C	5.19559000	0.16690300	0.00028700
H	4.75180600	2.27274100	-0.00001000
H	5.30944300	-1.98110500	0.00050100
H	6.26980100	0.30964000	0.00046700
O	-5.59167700	-0.34688500	0.00034600
H	-6.06724300	0.49369100	0.00097400

4-vinylphenylphenol anion

Charge=-1, Multiplicity=1

C	-2.38272400	1.32675100	0.00000200
C	-1.50755500	0.22727100	0.00000000
C	-2.09467100	-1.05263300	-0.00000300
C	-3.46577800	-1.22142800	-0.00000400
C	-4.36666400	-0.11325300	-0.00000400
C	-3.75907200	1.17371300	0.00000100
H	-1.96010200	2.32823300	0.00000500
H	-1.46276700	-1.93546000	-0.00000400
H	-3.89020300	-2.22087600	-0.00000600
H	-4.40577800	2.04571600	0.00000400
O	-5.65335700	-0.27395400	-0.00000100
C	-0.06596600	0.46211600	0.00000100
H	0.21187600	1.51430900	0.00000400
C	0.90789800	-0.46555700	-0.00000100
H	0.64269300	-1.51995900	-0.00000400
C	2.35289400	-0.20102400	0.00000000
C	2.90684400	1.09026900	-0.00000300
C	3.23492200	-1.29205500	0.00000400
C	4.28329100	1.27431100	-0.00000100
H	2.26123800	1.96093500	-0.00000700
C	4.61387100	-1.10727900	0.00000600
H	2.82620200	-2.29749700	0.00000600
C	5.14691700	0.17832200	0.00000300
H	4.68691800	2.28063700	-0.00000400
H	5.27133400	-1.96934300	0.00000900
H	6.22019200	0.32779600	0.00000400

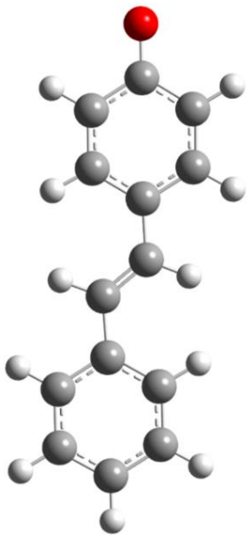

Cartesian coordinates of the optimized acids and conjugate bases of the cannabinoids studied at the M06-2X(SMD)/6-311++G(d,p) level of theory in water at 298.15 K.

Δ[8]-tetrahydrocannabinol

Charge=0, Multiplicity=1

C	1.24291800	1.20934500	0.29977700
C	-0.12903800	0.99293900	0.15903100
C	-0.74256300	-0.19626000	0.56938900
C	0.08075500	-1.11929200	1.23132600
C	1.44875100	-0.91991800	1.38238700
C	2.04493900	0.24596800	0.89960300
H	1.66303800	2.14658000	-0.04910200
H	2.04197600	-1.67364200	1.89236300
O	-0.52184400	-2.23470400	1.75535100
H	0.14263700	-2.77760600	2.19775400
O	-0.84000400	2.02142200	-0.40126400
C	-2.22010600	-0.43701300	0.33879400
H	-2.73208900	-0.44917900	1.31137600
C	-2.82471200	0.68610300	-0.51473900
H	-2.49465500	0.51912700	-1.54969800
C	-2.26827500	2.05786000	-0.12803100
C	-2.52775800	-1.76675700	-0.36695300
C	-3.98410400	-1.88106600	-0.75530900
C	-4.79793800	-0.82252000	-0.77349900
C	-4.34787900	0.58758700	-0.49329500
H	-4.77748100	1.25340500	-1.24735900
H	-4.74158500	0.92318500	0.47438700
C	-2.47564700	2.42121700	1.33929000
H	-2.02596500	3.39684800	1.53789000
H	-3.54156600	2.48411100	1.56547900
H	-2.02421600	1.68854300	2.01112200
C	-2.79751800	3.15850700	-1.03106800
H	-2.69114400	2.87969200	-2.08232700
H	-3.85155700	3.34691800	-0.82157300
H	-2.24214900	4.08160900	-0.85067500
C	-4.45148500	-3.26630100	-1.10216500
H	-4.33640600	-3.93839100	-0.24594100
H	-5.49739200	-3.27236200	-1.41358400
H	-3.84442600	-3.68171200	-1.91336900
C	3.53613300	0.43845500	0.99972400
H	3.90635500	-0.02814700	1.91760200
H	3.76428600	1.50678600	1.05734800
C	4.27125200	-0.16778600	-0.20215600

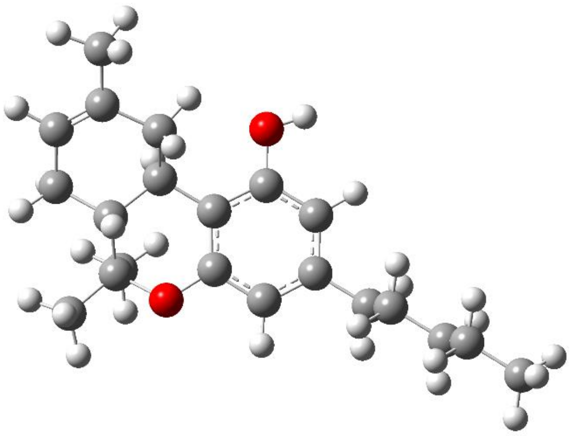

H	4.03877800	-1.23722400	-0.26223700
H	3.89165300	0.29124100	-1.12222700
C	5.78241700	0.02421000	-0.12011100
H	6.15613600	-0.42962900	0.80578900
H	6.00878600	1.09547500	-0.05644300
C	6.52449900	-0.57714900	-1.31103100
H	6.29791200	-1.64707700	-1.37198800
H	6.14717000	-0.12488000	-2.23454700
C	8.03408300	-0.37553700	-1.21930200
H	8.43400000	-0.84138400	-0.31428200
H	8.55073000	-0.81170400	-2.07729900
H	8.28229400	0.68894100	-1.18357800
H	-5.84630900	-0.96585400	-1.02561900
H	-1.90113200	-1.85939900	-1.26420300
H	-2.27522600	-2.61358000	0.27227900

Δ[8]-tetrahydrocannabinol anion

Charge=-1, Multiplicity=1

C	1.25360300	1.17057200	0.30185900
C	-0.11933800	0.94743200	0.16107900
C	-0.74171700	-0.23105600	0.57349700
C	0.04257600	-1.20463300	1.27787600
C	1.43907500	-0.96583700	1.38998900
C	2.04076200	0.19211500	0.90861400
H	1.67948900	2.10621900	-0.04422400
H	2.03900900	-1.71484600	1.90079000
O	-0.49937600	-2.26060700	1.81253300
O	-0.82461100	1.98737900	-0.41405000
C	-2.22315800	-0.44404000	0.34476000
H	-2.73721900	-0.45297100	1.31814100
C	-2.82590700	0.68198100	-0.50567900
H	-2.50906100	0.50822300	-1.54416300
C	-2.24578600	2.04727000	-0.13191300
C	-2.55726000	-1.76998400	-0.35539500
C	-4.01732400	-1.87047600	-0.73225200
C	-4.82013900	-0.80287500	-0.74509800
C	-4.35031900	0.60249800	-0.47078200
H	-4.78085900	1.27279300	-1.22081300
H	-4.73087300	0.94340200	0.50055900
C	-2.44397200	2.42267100	1.33443500
H	-1.97417100	3.39010900	1.52792700
H	-3.50780100	2.50877100	1.56374200
H	-2.00495300	1.68316800	2.00673300

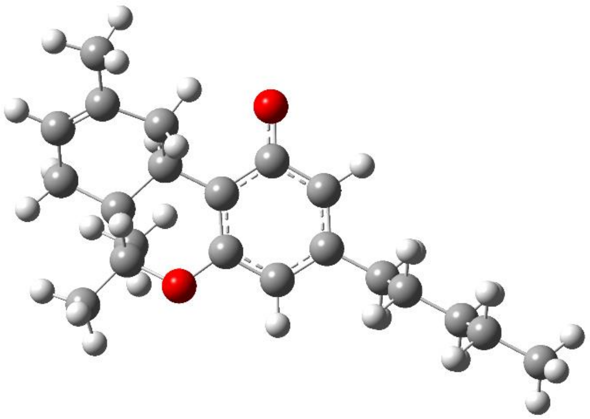

C	-2.76778500	3.15108500	-1.03692000
H	-2.66932200	2.86701800	-2.08774800
H	-3.81888800	3.35327600	-0.82475900
H	-2.20122100	4.06906800	-0.86410100
C	-4.50490600	-3.25033600	-1.07402900
H	-4.38931100	-3.92352400	-0.21869800
H	-5.55386600	-3.24509700	-1.37566300
H	-3.91037100	-3.67358000	-1.89054800
C	3.53533700	0.37884900	1.01411400
H	3.90557400	-0.11696600	1.91707400
H	3.76794300	1.44449600	1.10797300
C	4.27962400	-0.18590100	-0.20200300
H	4.04641800	-1.25233200	-0.30114800
H	3.90889200	0.30472500	-1.10946700
C	5.79091400	-0.00093300	-0.10257700
H	6.15623900	-0.48758100	0.81006200
H	6.01965200	1.06688600	0.00062000
C	6.54306700	-0.56207900	-1.30675700
H	6.31319700	-1.62822200	-1.40844800
H	6.17698900	-0.07557700	-2.21735300
C	8.05248300	-0.37028600	-1.19263800
H	8.44109100	-0.87004100	-0.30085000
H	8.57662500	-0.77741400	-2.06033900
H	8.30449100	0.69115700	-1.11588900
H	-5.87242700	-0.93460400	-0.98809500
H	-1.93969800	-1.87418400	-1.25830700
H	-2.30265600	-2.61277100	0.28804200

iso-tetrahydrocannabinol

Charge=0, Multiplicity=1

C	1.25681900	-1.37293000	-1.14823300
C	-0.12140100	-1.38786700	-0.96824600
C	-0.85704700	-0.21356100	-0.76250600
C	-0.13342100	0.98396300	-0.70319700
C	1.24898600	1.01587100	-0.88177100
C	1.95122300	-0.16309300	-1.11337200
H	1.77861900	-2.30962400	-1.31245000
H	1.76364200	1.96954600	-0.82982300
O	-0.74627700	2.18739700	-0.47117800
O	-0.73173800	-2.61990700	-1.00606200
C	3.45285300	-0.13814600	-1.24509800
H	3.75805600	0.76749200	-1.77801100
H	3.78376300	-0.99638600	-1.83712800

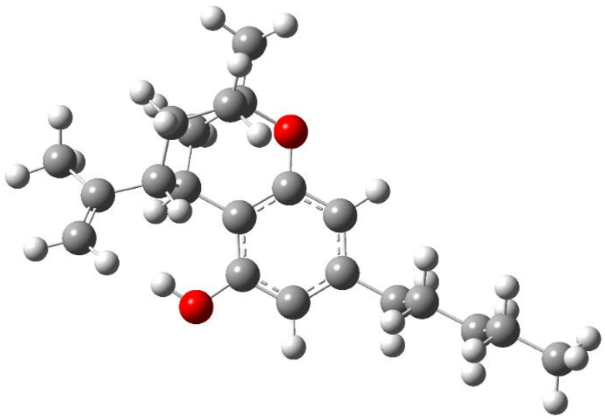

C	4.14721900	-0.17056900	0.12204300
H	3.80276700	0.68242600	0.71785700
H	3.84080500	-1.07609900	0.65834800
C	5.66789900	-0.13184600	0.00724800
H	5.96658500	0.77085200	-0.53948900
H	6.00937100	-0.98607800	-0.58986800
C	6.36784700	-0.15299600	1.36380500
H	6.02109200	0.69827900	1.95957300
H	6.07070100	-1.05630900	1.90743600
C	7.88753000	-0.10577200	1.23625400
H	8.20544600	0.80302500	0.71755400
H	8.37391700	-0.12128800	2.21425900
H	8.25622600	-0.96190200	0.66433800
H	-1.68269800	-2.54879900	-0.85110500
C	-2.10928300	2.12861500	0.02901500
C	-2.36208200	-0.23352800	-0.61022100
H	-2.77816700	-0.91645600	-1.35830300
C	-2.88398900	1.17558400	-0.87645900
H	-3.95330000	1.24520300	-0.66948900
H	-2.72071000	1.46554100	-1.91773800
C	-2.63192200	3.54657400	-0.06502800
H	-3.64241000	3.58658500	0.34807600
H	-1.99582500	4.22418400	0.50938100
H	-2.66329000	3.87884300	-1.10525900
C	-2.08687000	1.61159800	1.49148600
H	-2.28000600	2.44358700	2.17210900
H	-1.08321300	1.23909900	1.71932900
C	-2.78483000	-0.72066500	0.82128600
H	-1.92692700	-1.24052600	1.25957400
C	-3.09858300	0.48795200	1.72184900
H	-4.10675300	0.86349500	1.52621700
H	-3.07578600	0.17059400	2.76672400
C	-3.75174800	-2.97125600	1.17259800
H	-4.55159900	-3.70240000	1.10726400
H	-2.80921900	-3.29749000	1.60225700
H	-5.06111300	-0.89439400	-0.88492100
H	-5.65704700	-0.43502100	0.70423200
H	-5.94033500	-2.07568300	0.10022300
C	-3.92079600	-1.71735300	0.74616100
C	-5.21814600	-1.25894200	0.13517700

iso-tetrahydrocannabinol anion

Charge=-1, Multiplicity=1

C	1.27610100	-1.32621600	-1.17896800
C	-0.12836900	-1.42299600	-1.00175200
C	-0.84873500	-0.20820300	-0.76819300
C	-0.14719900	0.99242200	-0.68583300
C	1.23926900	1.06333900	-0.85557400
C	1.95055900	-0.10879400	-1.11210300
H	1.83116900	-2.24129400	-1.36947600
H	1.73961300	2.02295000	-0.77936400
O	-0.78364000	2.19168500	-0.43085100
O	-0.75107700	-2.56705200	-1.05326200
C	3.45487800	-0.06285800	-1.24003300
H	3.75591200	0.87332800	-1.72126200
H	3.79486400	-0.88293800	-1.88025000
C	4.15743200	-0.16879700	0.11906300
H	3.81580000	0.64938500	0.76356600
H	3.85496300	-1.10278300	0.60676200
C	5.67791500	-0.12345300	0.00073100
H	5.97535600	0.80863900	-0.49525600
H	6.01632800	-0.94231900	-0.64587600
C	6.38548100	-0.22296500	1.34987400
H	6.04531900	0.59464800	1.99463200
H	6.08761200	-1.15427000	1.84366200
C	7.90470100	-0.17456400	1.21686200
H	8.22397300	0.76084100	0.74872900
H	8.39657300	-0.24774200	2.18953600
H	8.26657400	-0.99834500	0.59517300
C	-2.13957100	2.09328200	0.07103900
C	-2.35229200	-0.25546700	-0.62897200
H	-2.73485800	-0.94193900	-1.38887800
C	-2.90407500	1.14953200	-0.85534400
H	-3.97400300	1.20158300	-0.64254000
H	-2.75032100	1.47363500	-1.88861700
C	-2.69153700	3.50339000	0.02211300
H	-3.70030400	3.51275200	0.44174600
H	-2.06579000	4.17789100	0.61187700
H	-2.73625300	3.86594700	-1.00771300
C	-2.10156800	1.53768900	1.51931100
H	-2.30474000	2.34775400	2.22387000
H	-1.09057200	1.17599500	1.73053000
C	-2.78681900	-0.78639000	0.77957100
H	-1.93801000	-1.33413100	1.19897800

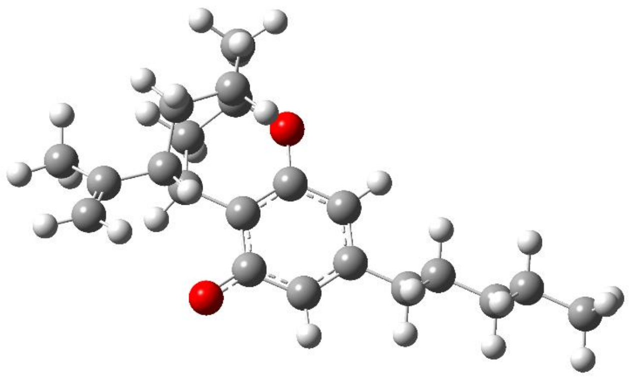

C	-3.09423400	0.39277000	1.72508200
H	-4.10906000	0.76546000	1.55719300
H	-3.05508700	0.04309700	2.75956000
C	-3.84289200	-2.99266800	1.17683800
H	-4.66840300	-3.69546800	1.11686200
H	-2.92610100	-3.33736500	1.64582300
H	-5.00831400	-0.99108900	-1.02087000
H	-5.62566500	-0.40286300	0.51641200
H	-5.96481000	-2.06624200	0.01183900
C	-3.94679200	-1.75394100	0.69017100
C	-5.20792500	-1.28047700	0.01549100

Δ⁹-tetrahydrocannabivarin

Charge=0, Multiplicity=1

C	2.04791500	1.06873100	-0.07340000
C	0.66154400	0.91956900	0.00746800
C	0.06853300	-0.25526600	0.48501500
C	0.94115600	-1.22804900	0.99500900
C	2.32307500	-1.09561800	0.92208500
C	2.88842300	0.05436700	0.36823600
H	2.45155200	1.99547600	-0.46669500
H	2.95414800	-1.88613600	1.31818800
O	0.37664600	-2.31799800	1.60758200
H	1.07653100	-2.90698100	1.91677900
O	-0.07463300	1.99882000	-0.40462300
C	-1.43949100	-0.39990000	0.52330900
H	-1.75148200	-0.30600300	1.57419500
C	-2.09926700	0.73242700	-0.27382100
H	-1.88075500	0.55529400	-1.33633300
C	-1.45793900	2.08277000	0.04439500
C	-1.97395500	-1.71854900	-0.00704900
C	-3.18334100	-1.83158800	-0.56440800
H	-1.34956600	-2.60017500	0.08106300
C	-4.11734300	-0.65728800	-0.73383600
H	-5.10240500	-0.92497300	-0.33718600
H	-4.26287900	-0.50135500	-1.81055400
C	-3.61079600	0.63608700	-0.08640900
H	-4.12292200	1.49019400	-0.53380600
H	-3.84168000	0.63821300	0.98405000
C	-1.47545700	2.44296300	1.52552400
H	-1.04703500	3.43764800	1.66807300
H	-2.50462700	2.45431400	1.89210800
H	-0.90422200	1.73131300	2.12516300

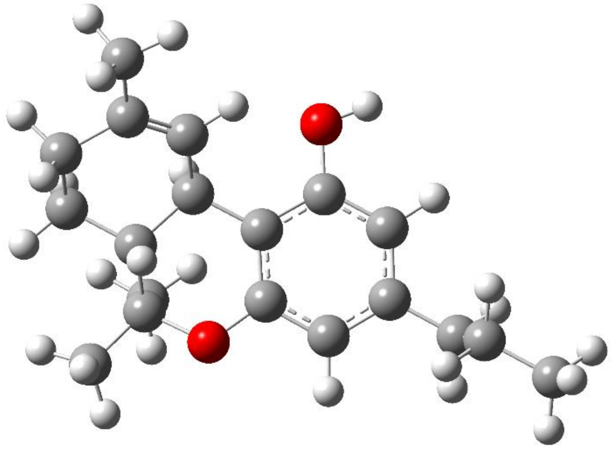

C	-2.05088800	3.20693100	-0.78793300
H	-2.11860600	2.91666500	-1.83931800
H	-3.04766700	3.46415500	-0.42742200
H	-1.41913800	4.09402200	-0.70497100
C	-3.69876800	-3.14122900	-1.09126200
H	-4.61257100	-3.43466600	-0.56481800
H	-3.95933700	-3.05103200	-2.15090100
H	-2.96199900	-3.93847800	-0.98009300
C	4.38388500	0.17550100	0.22895600
H	4.87313000	-0.29583200	1.08701600
H	4.66795100	1.23192400	0.22753500
C	4.89458200	-0.48259000	-1.05914700
H	4.60461200	-1.53815400	-1.05862900
H	4.39762500	-0.01750800	-1.91656300
C	6.40758800	-0.35689600	-1.20076600
H	6.91735700	-0.83420900	-0.35936600
H	6.70965000	0.69374400	-1.22277700
H	6.76150100	-0.82826800	-2.12020500

Δ⁹-tetrahydrocannabivarin anion

Charge=-1, Multiplicity=1

C	2.04528900	0.65327600	-0.72456700
C	0.69199900	0.66133900	-0.37318100
C	0.05104500	-0.45040400	0.17554200
C	0.84377200	-1.59193600	0.53023400
C	2.20871400	-1.58844700	0.13465600
C	2.80221800	-0.49373000	-0.48550900
H	2.48764500	1.54198000	-1.16200900
H	2.80426000	-2.46838100	0.36466800
O	0.34107800	-2.58693800	1.20228900
O	0.03562800	1.85418100	-0.60914400
C	-1.42980900	-0.39979100	0.48806200
H	-1.54305400	-0.33258400	1.58176300
C	-2.07540800	0.84547700	-0.13061200
H	-2.08289200	0.70359100	-1.22091800
C	-1.21140500	2.08483500	0.09930900
C	-2.21796000	-1.60887300	0.01820300
C	-3.51420500	-1.54676200	-0.30212900
H	-1.69603200	-2.55693300	-0.05021100
C	-4.30763900	-0.26221900	-0.24163100
H	-5.22471000	-0.43425800	0.33221800
H	-4.63672200	-0.02706400	-1.26228200
C	-3.52648000	0.92422700	0.33610800

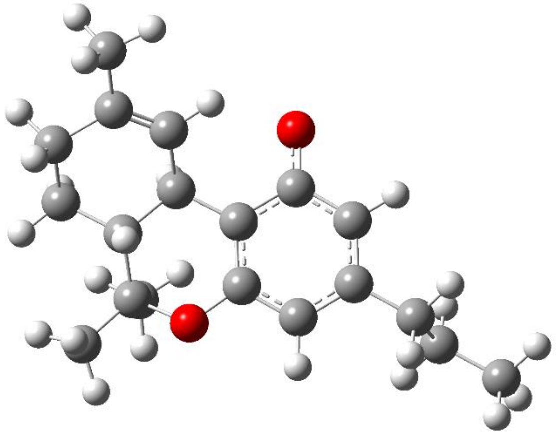

H	-3.99894800	1.85687800	0.02045200
H	-3.55413100	0.89663100	1.43061400
C	-0.90933100	2.36083600	1.56866200
H	-0.33005400	3.28280400	1.65954700
H	-1.84233400	2.48395900	2.12406300
H	-0.34074000	1.54819300	2.02487600
C	-1.79746200	3.32046300	-0.56432700
H	-2.09289100	3.10344500	-1.59403000
H	-2.67072500	3.67351100	-0.01400800
H	-1.05316000	4.12003900	-0.57116100
C	-4.28062500	-2.74987500	-0.77660100
H	-5.11425900	-2.96789000	-0.10115900
H	-4.71522800	-2.56365400	-1.76438500
H	-3.64373600	-3.63399100	-0.83780500
C	4.27623800	-0.50688200	-0.81344500
H	4.46019400	0.08653800	-1.71503300
H	4.59722500	-1.53149300	-1.02662800
C	5.13302800	0.05284300	0.32906900
H	4.81043600	1.07598800	0.54738100
H	4.94832200	-0.53692000	1.23278100
C	6.62012400	0.03692600	-0.00886400
H	6.82273300	0.63877800	-0.89915600
H	6.96208200	-0.98216900	-0.20979800
H	7.22045900	0.43725400	0.81118400

3-homotetrahydrocannibinol

Charge=0, Multiplicity=1

C	1.09930488	1.47699663	-1.33143088
C	-0.22650312	1.48395263	-0.91745788
C	-0.90119212	0.34083863	-0.44830688
C	-0.11650412	-0.82764937	-0.37017388
C	1.21764088	-0.84474437	-0.77224988
C	1.83710188	0.29909163	-1.26747688
H	1.54166088	2.39861163	-1.69337388
H	1.77491188	-1.77373237	-0.69050088
O	-0.67288912	-1.98178937	0.11512512
H	-0.00694112	-2.68095137	0.10127912
O	-0.89300912	2.66899263	-1.04034388
C	-2.35503012	0.46521163	-0.17128488
C	-2.88883912	1.70021663	-0.08510488
C	-1.96179312	2.90676463	-0.09117088
C	-3.22054812	-0.77506037	-0.05303588
C	-4.63378612	-0.50109137	0.46151712

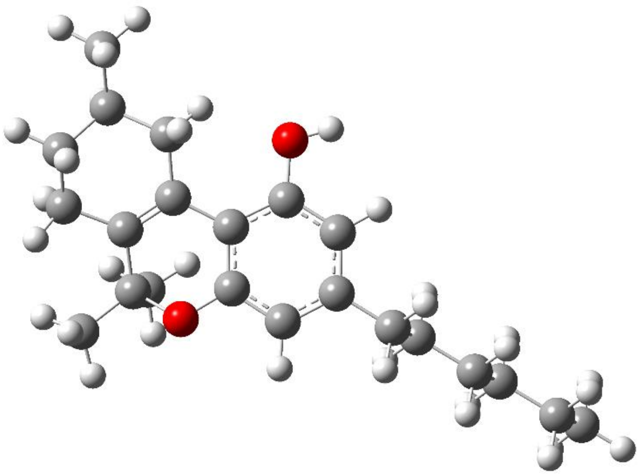

C	-5.20986512	0.72384263	-0.23236488
H	-6.24597312	0.89166663	0.07523312
H	-5.21501312	0.54667663	-1.31526488
C	-4.36686012	1.95451863	0.07821312
H	-4.66490012	2.77615663	-0.57689988
H	-4.56281412	2.30075163	1.10230312
C	-1.36209112	3.12229063	1.29861512
H	-0.69376812	3.98716163	1.29134212
H	-2.16672512	3.30682463	2.01420012
H	-0.80577812	2.24155763	1.62710912
C	-2.60761412	4.19048063	-0.58716288
H	-3.04939112	4.05116063	-1.57598788
H	-3.38257412	4.52349963	0.10415112
H	-1.84321612	4.96769963	-0.64723288
C	3.29521588	0.27792363	-1.64333388
H	3.48460988	1.02876763	-2.41596488
H	3.55135288	-0.69998837	-2.06186888
C	4.19920788	0.55812663	-0.43607288
H	3.94121288	1.53668563	-0.01526988
H	3.99624088	-0.18564937	0.34289812
C	5.68122288	0.52897863	-0.79725688
H	5.87773088	1.27106763	-1.58036588
H	5.92942788	-0.45006837	-1.22445188
C	6.58882288	0.80303463	0.39872312
H	6.34260488	1.78355463	0.82419312
H	6.38784788	0.06353463	1.18351112
C	8.07356688	0.76783863	0.04510012
H	8.27344988	1.50631263	-0.73891288
H	8.31798788	-0.21193537	-0.37949188
C	8.96725188	1.04304363	1.25067412
H	10.02577688	1.01390863	0.98226012
H	8.75561388	2.02885563	1.67426112
H	8.79980288	0.30050863	2.03596512
H	-4.56250012	-0.27418137	1.53348312
H	-3.27209312	-1.27265537	-1.03140788
H	-2.74461712	-1.48541737	0.61959912
C	-5.51623112	-1.73050737	0.27882112
H	-5.07437912	-2.61066337	0.75427812
H	-6.50712512	-1.57122337	0.71203212
H	-5.64389312	-1.95155137	-0.78583088

3-homotetrahydrocannibinol anion

Charge=-1, Multiplicity=1

C	0.87267600	0.50218400	-1.06543200
C	-0.45835100	0.57125100	-0.67541000
C	-1.18933200	-0.51701200	-0.16914500
C	-0.47805600	-1.75280400	0.04581200
C	0.88432300	-1.79777600	-0.37718700
C	1.55016200	-0.71445800	-0.92715800
H	1.36271900	1.38586900	-1.45932900
H	1.40945400	-2.73934700	-0.23935200
O	-1.00606000	-2.79929800	0.60052400
O	-1.07852400	1.78513400	-0.86062500
C	-2.64187400	-0.30724200	0.04642000
C	-3.12583700	0.95156600	0.10013800
C	-2.13477700	2.10514200	0.07752800
C	-3.57796500	-1.50010300	0.11181800
C	-4.97689000	-1.16390200	0.62562400
C	-5.49306000	0.08644500	-0.07234000
H	-6.52126800	0.30439400	0.23183000
H	-5.50619100	-0.09846000	-1.15410800
C	-4.59410800	1.28095500	0.22819100
H	-4.84558700	2.10249800	-0.44692300
H	-4.79387500	1.65665600	1.24169700
C	-1.53094000	2.32869600	1.46524500
H	-0.80559400	3.14642700	1.43639500
H	-2.32656700	2.59240900	2.16624800
H	-1.03740400	1.42518300	1.82929000
C	-2.70892000	3.41009100	-0.45248000
H	-3.14628300	3.27226900	-1.44372300
H	-3.47508400	3.79650100	0.22100600
H	-1.90670800	4.14790300	-0.52024800
C	3.00983400	-0.81177800	-1.29720100
H	3.19858300	-0.23184100	-2.20666200
H	3.26348900	-1.85376400	-1.51487100
C	3.92956900	-0.29700500	-0.18300500
H	3.67781500	0.74642100	0.03855100
H	3.73779300	-0.87000500	0.73159200
C	5.40724200	-0.39899700	-0.55012000
H	5.59155700	0.17083300	-1.46894800
H	5.65192500	-1.44387300	-0.77642100
C	6.33263000	0.10823200	0.55248300
H	6.08899300	1.15351300	0.77876400
H	6.14741900	-0.46065800	1.47183600

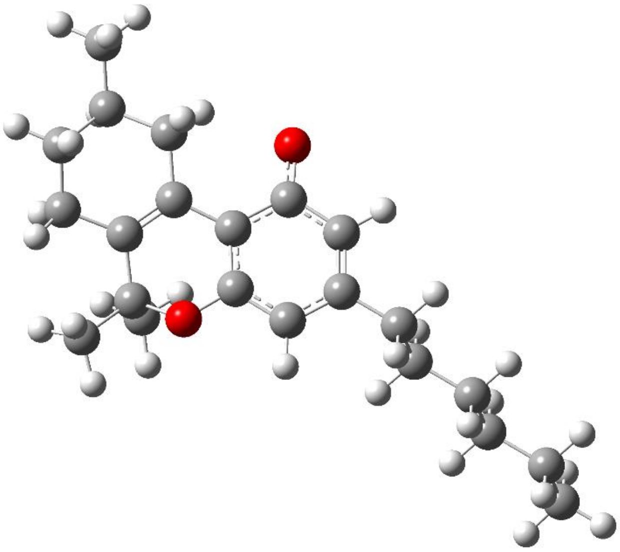

C	7.81172600	0.00535300	0.18824100
H	7.99555900	0.57361600	-0.73008200
H	8.05374000	-1.03890300	-0.03753600
C	8.72391800	0.51578100	1.29968400
H	9.77801200	0.43597600	1.02411800
H	8.51401800	1.56563600	1.52286100
H	8.57324300	-0.05645200	2.21941000
H	-4.89834100	-0.94156500	1.69841700
H	-3.65504200	-1.94309100	-0.89204700
H	-3.13345400	-2.26840600	0.73983900
C	-5.91840600	-2.34833800	0.43883500
H	-5.52451600	-3.24855300	0.91887300
H	-6.90414400	-2.14009800	0.86350600
H	-6.04897700	-2.56547500	-0.62643400

nabilone

Charge=0, Multiplicity=1

C	0.16919800	-1.51712700	-0.27727700
C	1.47665900	-1.05970600	-0.14100500
C	1.77285600	0.27444200	0.16691200
C	0.67332100	1.09199500	0.44740300
C	-0.64241000	0.65114500	0.31232700
C	-0.91498200	-0.66270300	-0.07276000
H	0.02652200	-2.56132100	-0.52997600
H	-1.43473300	1.35713800	0.52970800
O	0.93369700	2.36403200	0.89051200
H	0.10163300	2.81540100	1.07946900
O	2.45806400	-1.99846000	-0.32439500
C	3.20567200	0.75634800	0.24565800
H	3.44322500	0.97051200	1.29684000
C	4.17004900	-0.32642800	-0.26227800
H	4.06324800	-0.37663900	-1.35512100
C	3.76573700	-1.71348900	0.24476400
C	3.47069300	2.04261400	-0.56542500
C	4.91492800	2.46228200	-0.43389000
C	5.95571200	1.39760400	-0.65721200
H	6.93128800	1.78156500	-0.35823500
H	5.97390600	1.22047400	-1.74016300
C	5.60857100	0.08010700	0.05095300
H	6.31150400	-0.68585400	-0.27972000
H	5.73189800	0.19945600	1.13199100
C	3.67890500	-1.81406300	1.76414700
H	3.39334100	-2.82993100	2.04633500

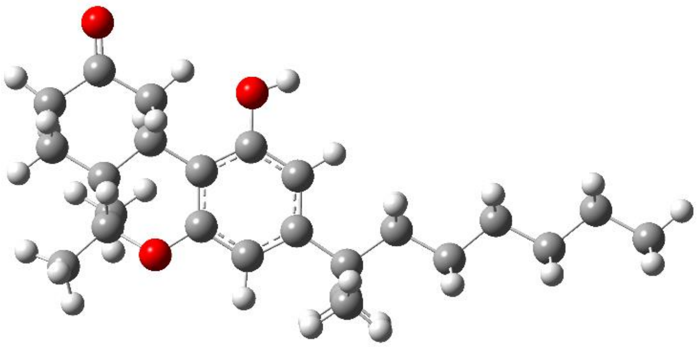

H	4.65212300	-1.59488200	2.20847800
H	2.94612200	-1.12003000	2.18062600
C	4.65953900	-2.80816600	-0.31326800
H	4.78816400	-2.69095300	-1.39209900
H	5.63959400	-2.78361400	0.16456300
H	4.20756300	-3.78220100	-0.11395200
C	-2.34262400	-1.20622200	-0.23516700
C	-3.38376000	-0.09235700	-0.00758100
H	-3.26468800	0.29634100	1.01070700
H	-3.16459700	0.73786500	-0.68956900
C	-4.84865500	-0.48745200	-0.19801400
H	-5.10806000	-1.32650400	0.45585400
H	-5.02173500	-0.82159900	-1.22600200
C	-5.79095000	0.67766000	0.10147300
H	-5.63280800	1.01748400	1.13217600
H	-5.53854700	1.52466900	-0.54813500
C	-7.26164400	0.31725200	-0.08989400
H	-7.51548000	-0.52819700	0.56137700
H	-7.42026400	-0.02531000	-1.11984200
H	3.28627400	1.83116100	-1.62604400
H	2.83994600	2.87221800	-0.25918300
O	5.21936400	3.61551200	-0.17923800
C	-2.49443000	-1.77963700	-1.65399700
H	-2.40258100	-0.98815000	-2.40392600
H	-1.73287000	-2.53459400	-1.85845300
H	-3.46988900	-2.25563200	-1.77346600
C	-2.55092000	-2.33416200	0.79024400
H	-2.47299500	-1.94805100	1.81090500
H	-3.53531800	-2.79081700	0.66812800
H	-1.80437800	-3.12127900	0.66496600
C	-8.20886700	1.47797300	0.20344900
H	-8.04923200	1.82095600	1.23151200
H	-7.95670800	2.32096700	-0.44910900
C	-9.67414400	1.09889000	0.01003100
H	-9.95239800	0.27383800	0.67181000
H	-10.33920200	1.93883000	0.22362000
H	-9.85959800	0.77720200	-1.01865800

nabilone anion

Charge=-1, Multiplicity=1

C	0.17494500	-1.51105300	-0.27437800
C	1.47811200	-1.03619300	-0.14136100
C	1.77366200	0.29250300	0.17138000
C	0.68750100	1.15834300	0.50669300
C	-0.63629600	0.66074500	0.33071900
C	-0.90718100	-0.64629500	-0.06156600
H	0.03403300	-2.55521100	-0.52716000
H	-1.43505600	1.35768800	0.55270900
O	0.88480700	2.35788800	0.97460400
O	2.46864500	-1.97978800	-0.33895600
C	3.20787700	0.76525100	0.24080800
H	3.45710400	0.99038600	1.28866900
C	4.17844300	-0.31117500	-0.26639200
H	4.07533100	-0.35984200	-1.36001200
C	3.77182300	-1.70012100	0.23281100
C	3.46873200	2.04934900	-0.57620000
C	4.90556700	2.48097400	-0.42793100
C	5.95658100	1.42408300	-0.64902200
H	6.92754400	1.81382400	-0.34199200
H	5.98345900	1.25287200	-1.73277600
C	5.61574000	0.09851100	0.04900600
H	6.32396200	-0.66075600	-0.28698500
H	5.73921400	0.21088000	1.13087800
C	3.68833400	-1.80621500	1.75275400
H	3.39755100	-2.82158800	2.03245200
H	4.66325200	-1.59445000	2.19737300
H	2.95957100	-1.10911500	2.17059900
C	4.66867700	-2.79265000	-0.32684600
H	4.79478900	-2.67492600	-1.40610600
H	5.65035700	-2.76589500	0.14796600
H	4.22071800	-3.76885200	-0.12794400
C	-2.33704400	-1.19207400	-0.22772300
C	-3.38486800	-0.08446100	0.00209500
H	-3.27223200	0.29788100	1.02331000
H	-3.16405300	0.75215200	-0.67109100
C	-4.84878700	-0.47925100	-0.19933900
H	-5.11161100	-1.32565500	0.44384700
H	-5.01764700	-0.80207700	-1.23174700
C	-5.79318700	0.68226800	0.10821700
H	-5.64044000	1.01055500	1.14348700
H	-5.53671600	1.53663900	-0.53012000

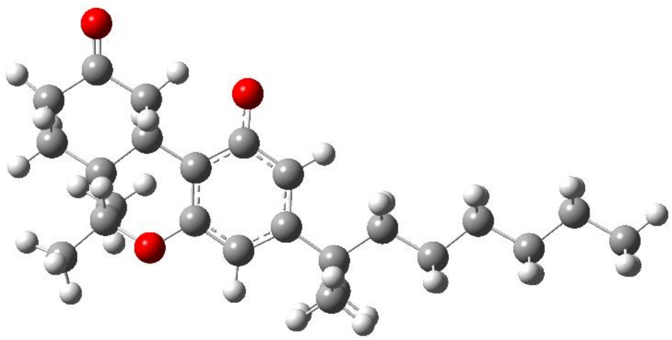

C	-7.26335000	0.32615600	-0.09504600
H	-7.52232500	-0.52562000	0.54592400
H	-7.41697100	-0.00524500	-1.12943100
H	3.29974000	1.82711700	-1.63734600
H	2.81806700	2.86748000	-0.27980900
O	5.20378800	3.63320300	-0.15664900
C	-2.49153200	-1.76218900	-1.64775700
H	-2.39902400	-0.96873000	-2.39597500
H	-1.72966500	-2.51605000	-1.85476100
H	-3.46726100	-2.23767400	-1.77062800
C	-2.54871700	-2.32479100	0.79187000
H	-2.46798700	-1.94377700	1.81449400
H	-3.53441300	-2.77997500	0.67103400
H	-1.80354800	-3.11255000	0.66255400
C	-8.21011100	1.48557000	0.20521800
H	-8.05526600	1.81762000	1.23759300
H	-7.95270300	2.33477000	-0.43716200
C	-9.67516900	1.11183200	0.00006800
H	-9.95890000	0.28073800	0.65189900
H	-10.33945800	1.95111500	0.21865900
H	-9.85584600	0.80115100	-1.03285600

cannabinol

Charge=0, Multiplicity=1

C	-1.53878288	-1.85712912	0.23232674
C	-0.18259388	-1.66079412	0.01205574
C	0.46429912	-0.44323012	0.27804474
C	-0.32098488	0.56164188	0.86849474
C	-1.68106188	0.37481488	1.10191174
C	-2.30480288	-0.82734212	0.77489374
H	-1.97943588	-2.81891712	-0.00561526
H	-2.25080888	1.18057288	1.55551374
O	0.28424212	1.73329188	1.23013674
H	-0.35967988	2.30813588	1.66383074
O	0.52719612	-2.70534712	-0.51631526
C	1.89059112	-0.33265612	-0.09137426
C	2.60924012	-1.52562112	-0.28032126
C	1.89435012	-2.84367412	-0.04214426
C	2.53436912	0.88973588	-0.33488526
C	3.87378812	0.94798388	-0.71190726
C	4.57994812	-0.24772312	-0.85847226
C	3.94985212	-1.46893712	-0.65472226
C	1.87792612	-3.19630012	1.44452274

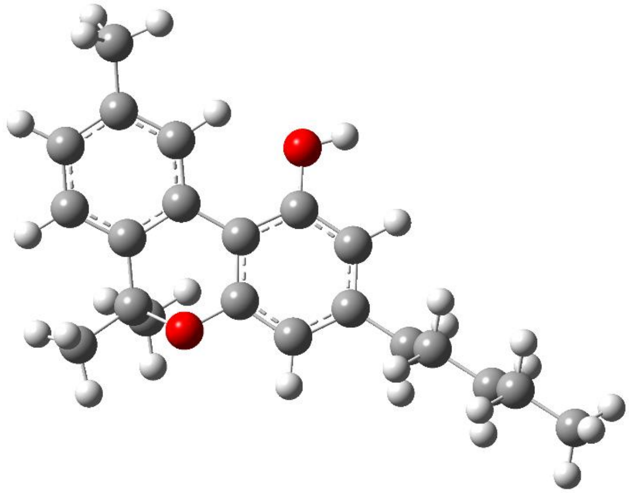

H	1.31268012	-4.11834312	1.60122574
H	2.90334312	-3.34804512	1.78752774
H	1.43134112	-2.39793412	2.04123274
C	2.45517112	-3.99593912	-0.85768326
H	2.50734012	-3.73506312	-1.91648126
H	3.45169212	-4.26628112	-0.50779126
H	1.80472412	-4.86397712	-0.73495626
C	4.55162912	2.26952688	-0.96081926
H	5.36997212	2.42341388	-0.25275726
H	4.97940012	2.30347588	-1.96562526
H	3.84948412	3.09747288	-0.85674626
C	-3.78808688	-0.99676912	0.97192274
H	-4.11733288	-0.39067812	1.82092274
H	-4.00901988	-2.04242512	1.20529474
C	-4.57729288	-0.58397412	-0.27716626
H	-4.34964288	0.46130388	-0.51543626
H	-4.23902888	-1.18472412	-1.12910926
C	-6.08261088	-0.75259112	-0.09637126
H	-6.41411188	-0.15465512	0.76120474
H	-6.30349388	-1.79872812	0.14784874
C	-6.87961688	-0.34502212	-1.33296926
H	-6.65608388	0.69939188	-1.57592026
H	-6.54612888	-0.94297012	-2.18796126
C	-8.38315888	-0.51780212	-1.13956926
H	-8.74018988	0.09271088	-0.30542026
H	-8.93977388	-0.22416812	-2.03245926
H	-8.62886788	-1.56026412	-0.91844726
H	5.62571912	-0.22190412	-1.14762226
H	4.51326112	-2.38415812	-0.79313026
H	1.98168412	1.81407888	-0.24131026

cannabinol anion

Charge=-1, Multiplicity=1

C	1.53024731	1.47941934	0.24875327
C	0.17118831	1.34123534	0.01366727
C	-0.53682769	0.14876634	0.23142527
C	0.16652131	-0.96791466	0.80265727
C	1.56524131	-0.79666866	1.03105627
C	2.23944531	0.38153634	0.75673027
H	2.01655831	2.42836134	0.05166927
H	2.10405231	-1.64217166	1.45002827
O	-0.41343669	-2.08097566	1.11572027
O	-0.48313169	2.44198034	-0.49906273
C	-1.95878969	0.13099534	-0.15524773
C	-2.62247769	1.36441334	-0.32544373
C	-1.84396769	2.63527634	-0.03835973
C	-2.66987169	-1.04668766	-0.43090973
C	-4.00808569	-1.02686566	-0.82587273
C	-4.64920769	0.20354934	-0.96301973
C	-3.95522269	1.38664734	-0.72146473
C	-1.83201069	2.94021534	1.46000727
H	-1.21291369	3.81961034	1.65577827
H	-2.85122069	3.14535434	1.79395127
H	-1.44452369	2.09686034	2.03546627
C	-2.33638269	3.84212134	-0.81983273
H	-2.38077269	3.62220534	-1.88842673
H	-3.32619169	4.14686734	-0.47893873
H	-1.64927769	4.67465334	-0.65580673
C	-4.74477669	-2.31688566	-1.07757173
H	-5.13339869	-2.72784566	-0.14115673
H	-5.59116469	-2.16203266	-1.74865573
H	-4.08428269	-3.06744466	-1.51564973
C	3.72948331	0.48933334	0.96871627
H	4.05821831	-0.28496066	1.66820227
H	3.96734231	1.45929134	1.41823327
C	4.51252131	0.34820134	-0.34233873
H	4.27701531	-0.62099866	-0.79712573
H	4.17704831	1.11790534	-1.04661173
C	6.02023331	0.46585134	-0.14010373
H	6.35297431	-0.30195766	0.56898327
H	6.24915531	1.43517234	0.31946027
C	6.80933031	0.32639534	-1.43953473
H	6.58163031	-0.64315466	-1.89555773
H	6.47246831	1.09121234	-2.14769073

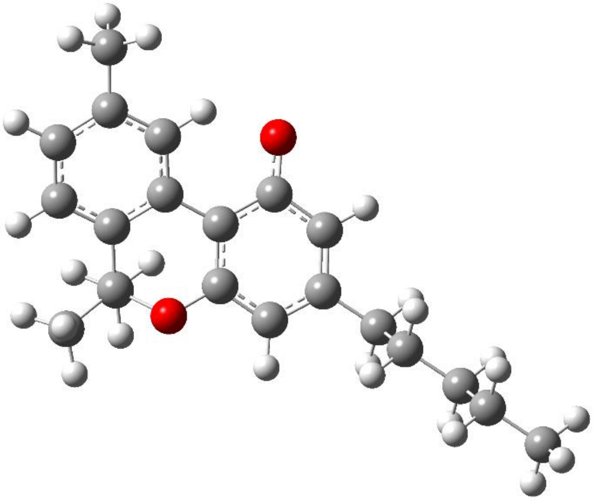

C	8.31458431	0.45249934	-1.22424973
H	8.67488731	-0.31884266	-0.53777273
H	8.86501631	0.35021134	-2.16229573
H	8.56487631	1.42567334	-0.79256573
H	-5.68916469	0.23991834	-1.27054673
H	-4.46700369	2.33354034	-0.84947073
H	-2.16342669	-1.99767266	-0.34133173

cannabichromene

Charge=0, Multiplicity=1

C	2.19651400	-2.12915800	0.85822900
C	0.93201300	-2.62268500	1.16942000
C	-0.18032200	-2.32164300	0.37511300
C	0.02885600	-1.48782000	-0.73606100
C	1.28339600	-0.99860000	-1.06370000
C	2.38151700	-1.32235900	-0.26168300
H	3.03188200	-2.38532000	1.50071700
H	1.39785500	-0.36940200	-1.93963800
O	-1.01208600	-1.20807600	-1.57970200
O	0.84313400	-3.42221200	2.27935900
C	3.73673200	-0.74153000	-0.57095100
H	3.88774700	-0.72450000	-1.65466900
H	4.51494000	-1.37654100	-0.13824300
C	3.88607000	0.68410800	-0.02443400
H	3.09706900	1.31508200	-0.44914100
H	3.73130500	0.66973900	1.06053000
C	5.25027800	1.28892400	-0.34085200
H	5.40210000	1.29113700	-1.42707200
H	6.03676800	0.65301900	0.08324600
C	5.40928300	2.71053200	0.19246100
H	4.62102900	3.34264500	-0.23050500
H	5.25778600	2.70629200	1.27727800
C	6.77583000	3.30540800	-0.13390300
H	6.93609600	3.33976300	-1.21517900
H	6.87424800	4.32252800	0.25219700
H	7.57658800	2.70172800	0.30236200
C	-1.52635800	-2.85685600	0.57749400
H	-1.68135400	-3.70349500	1.23811600
C	-2.55478700	-2.32678000	-0.08768800
H	-3.56236200	-2.71273100	0.02083800
C	-2.34633700	-1.14313600	-1.00184700
C	-3.28562400	-1.19373800	-2.19489200
H	-4.32302300	-1.20699500	-1.85826100

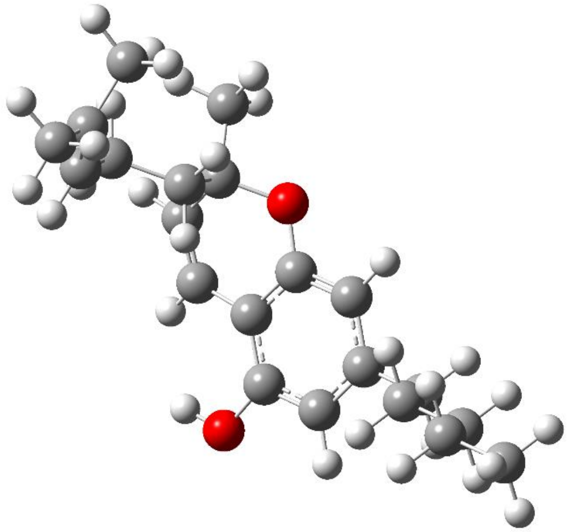

H	-3.13239200	-0.31988100	-2.83234500
H	-3.10143800	-2.09902400	-2.77685500
C	-2.45409500	0.18401000	-0.23718300
H	-2.23509000	0.99460900	-0.94114800
H	-1.67947700	0.20143400	0.53798700
C	-3.82170200	0.43152800	0.41292600
H	-4.59961400	0.43104500	-0.35399800
H	-4.05127900	-0.38767600	1.10000500
C	-3.83391600	1.73352300	1.17246500
C	-4.06803800	2.94104900	0.64784500
C	-4.02922400	4.18740100	1.48978800
H	-3.80176600	3.96340800	2.53325500
H	-3.27532300	4.88417100	1.10923900
H	-4.99076900	4.70930000	1.44609200
C	-4.37339800	3.17824500	-0.80641900
H	-4.39009600	2.26251700	-1.39744900
H	-5.34064100	3.68004800	-0.91379200
H	-3.62338700	3.84921800	-1.23802800
H	-0.07200400	-3.66869600	2.45778400
H	-3.59391300	1.67634300	2.23266200

cannabichromene anion

Charge=-1, Multiplicity=1

C	2.40981600	-2.01083726	1.65668013
C	1.13354500	-2.52661626	2.02380713
C	0.06202100	-2.28946026	1.10620113
C	0.30059100	-1.57077526	-0.07156587
C	1.55534400	-1.08461426	-0.40914787
C	2.62166200	-1.31390926	0.47482013
H	3.23951300	-2.18181026	2.33733313
H	1.69693500	-0.54070926	-1.33661287
O	-0.71896800	-1.40090126	-0.98477087
O	0.95302000	-3.18156826	3.12843713
C	3.98143100	-0.73867426	0.16110913
H	4.18172300	-0.83056926	-0.91145287
H	4.75173600	-1.30908626	0.68877413
C	4.09214500	0.73819174	0.56016013
H	3.32028700	1.31181074	0.03443913
H	3.88370700	0.83516774	1.63195513
C	5.46522700	1.32713774	0.25107613
H	5.67205500	1.22193174	-0.82097787
H	6.23484300	0.74774974	0.77556713
C	5.58690700	2.79704374	0.64513313

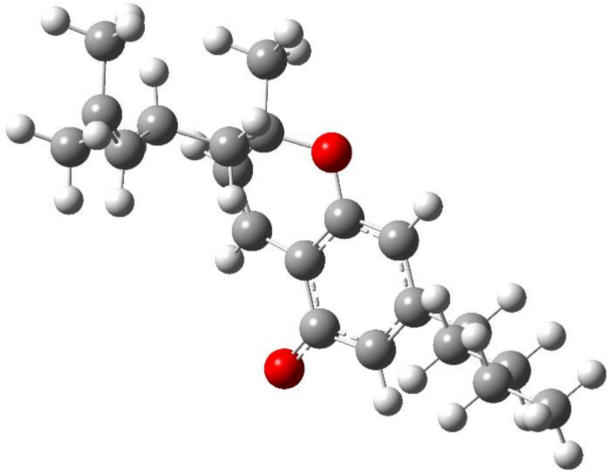

H	4.81800100	3.37422474	0.12022213
H	5.37822800	2.90004174	1.71550113
C	6.96502100	3.37237474	0.33238513
H	7.18206100	3.30138074	-0.73715087
H	7.03682900	4.42379874	0.61999413
H	7.74531600	2.82395074	0.86757513
C	-1.27463200	-2.83373026	1.30386413
H	-1.41324600	-3.60341726	2.05575813
C	-2.29527100	-2.41218226	0.54835913
H	-3.29567600	-2.81930726	0.64721013
C	-2.07087700	-1.30623926	-0.45744787
C	-2.97358300	-1.46303426	-1.66942687
H	-4.02072200	-1.45760726	-1.36263187
H	-2.80965800	-0.64453626	-2.37443787
H	-2.76747900	-2.41193726	-2.16901487
C	-2.21687700	0.08018774	0.19052813
H	-1.95552200	0.83515874	-0.55946787
H	-1.48545800	0.15581174	1.00321613
C	-3.61695200	0.37229774	0.74463113
H	-4.34139100	0.40418474	-0.07003087
H	-3.91429600	-0.44977326	1.40584613
C	-3.62795700	1.65170574	1.53287713
C	-4.24856300	2.79845874	1.23412513
C	-4.14337900	3.99615974	2.14114013
H	-3.53147600	3.78645874	3.01988413
H	-3.70682300	4.84564274	1.60587613
H	-5.13717600	4.31199274	2.47525413
C	-5.09154800	3.03151374	0.00970213
H	-5.12326500	2.17830874	-0.66614687
H	-6.11758900	3.27850674	0.30171713
H	-4.70870100	3.89436674	-0.54463487
H	-3.03715600	1.62678974	2.44858113

cannabigerol

Charge=0, Multiplicity=1

C	2.57542600	-1.14672000	1.48678800
C	1.24559300	-1.54845700	1.59976900
C	0.48797000	-1.91938900	0.48684900
C	1.11880900	-1.84757100	-0.75822800
C	2.44663400	-1.45067100	-0.89402300
C	3.18543000	-1.09901800	0.23486100
H	3.12876100	-0.87356600	2.38052700
H	2.89739700	-1.41858800	-1.88165100
O	0.36328200	-2.19453700	-1.84872500
H	0.89734200	-2.11668800	-2.64924500
O	0.62262700	-1.59628500	2.82283600
C	4.60440700	-0.61013400	0.09580000
H	5.07767700	-1.09861000	-0.76111000
H	5.17523600	-0.88377600	0.98809400
C	4.66986400	0.91033600	-0.09443800
H	4.09181600	1.18514100	-0.98417500
H	4.18799700	1.39896200	0.76018000
C	6.10090700	1.41954200	-0.23576300
H	6.58001100	0.92342600	-1.08857700
H	6.67611200	1.13604900	0.65401700
C	6.17898200	2.93242900	-0.42469400
H	5.60341300	3.21340100	-1.31327300
H	5.69888200	3.42577100	0.42732800
C	7.61501000	3.42870800	-0.56413600
H	8.10427200	2.96518400	-1.42541400
H	7.65467300	4.51201900	-0.69891900
H	8.20025400	3.17927700	0.32539300
H	1.24960800	-1.34074400	3.51085400
C	-0.96254400	-2.32895400	0.59804800
H	-1.16103600	-3.15464100	-0.08490000
H	-1.14764600	-2.69571000	1.61008900
C	-2.77439100	-1.03537800	-0.66006300
C	-3.58974100	0.22935100	-0.78799300
H	-3.24842700	0.96621700	-0.05510200
H	-3.43230500	0.66084500	-1.78435700
C	-5.10134500	0.00063200	-0.59224800
H	-5.26096300	-0.46164300	0.38645500
H	-5.46184500	-0.70904400	-1.34085900
C	-5.87889200	1.28502500	-0.71753400
C	-6.06054900	2.18999200	0.25050600
C	-6.82920800	3.46064700	0.00843500

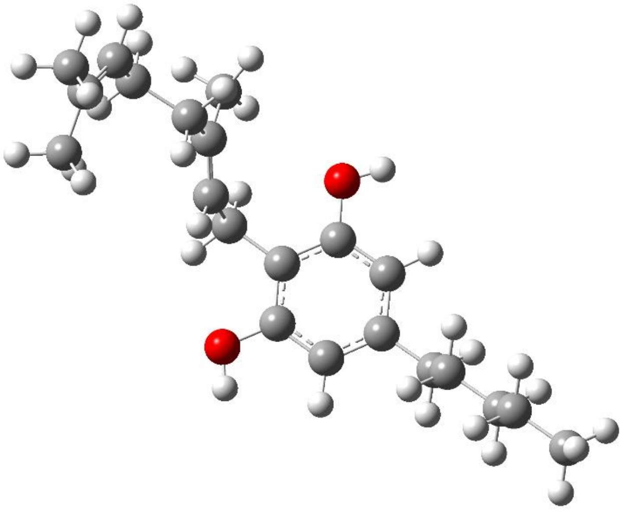

H	-7.68717200	3.52471300	0.68571000
H	-6.20123300	4.33398000	0.21266000
H	-7.19139800	3.52450800	-1.01896400
C	-1.87142500	-1.15732400	0.31958600
H	-1.74140700	-0.31487500	0.99864000
H	-6.29240300	1.50452900	-1.70051900
C	-3.07762000	-2.09748300	-1.68397700
H	-3.14056300	-1.64683100	-2.67949000
H	-2.33078900	-2.88905900	-1.71352100
H	-4.04969500	-2.55943700	-1.48498600
C	-5.51888900	2.04335500	1.64651300
H	-4.98726500	1.10509800	1.80387800
H	-4.83144600	2.86678300	1.86696600
H	-6.33169400	2.11039000	2.37702900

cannabigerol anion C1′

Charge=-1, Multiplicity=1

C	0.99696609	-1.41205529	2.21990056
C	-0.35200406	-1.86417507	2.24851055
C	-0.95862180	-2.19178039	0.99754772
C	-0.22836089	-2.01758466	-0.17478282
C	1.09139174	-1.56522812	-0.18357688
C	1.70771335	-1.26355082	1.03502856
H	1.47208963	-1.17227899	3.16766018
H	1.62196770	-1.45324003	-1.12478072
O	-0.87186522	-2.31803215	-1.36143234
H	-0.26161819	-2.17449364	-2.09497099
O	-1.00474453	-1.97731979	3.37147791
C	3.11530276	-0.71693379	1.04806802
H	3.70506988	-1.19660085	0.26004971
H	3.59263662	-0.95464004	2.00373848
C	3.15098002	0.80174662	0.83755422
H	2.66861195	1.04335577	-0.11655768
H	2.55853931	1.28530821	1.62294345
C	4.56959093	1.36325247	0.84824748
H	5.15957622	0.87304589	0.06410140
H	5.05105300	1.11495878	1.80202092
C	4.61548842	2.87509436	0.64071802
H	4.13242570	3.12158382	-0.31101097
H	4.02682465	3.36287570	1.42519333
C	6.03975988	3.42228199	0.65112167
H	6.63714177	2.96559109	-0.14301105
H	6.05620854	4.50446241	0.50281324

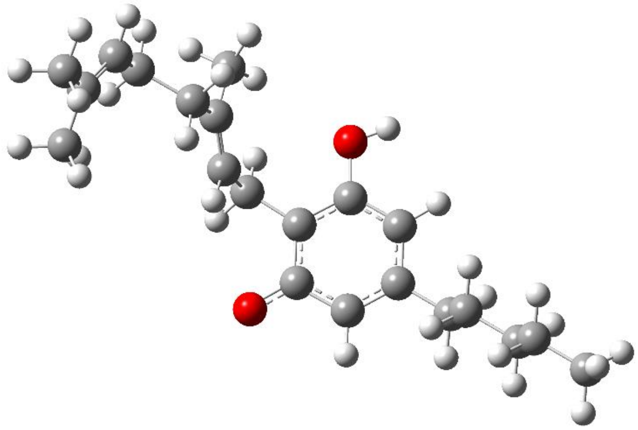

H	6.53169913	3.20666985	1.60377458
C	-2.39136169	-2.67133939	0.94460047
H	-2.51802185	-3.39644557	0.14068623
H	-2.61305462	-3.18580119	1.88393085
C	-4.20178512	-1.28504035	-0.21809017
C	-5.07417317	-0.05184433	-0.21019306
H	-4.84163140	0.56099204	0.66578017
H	-4.85571463	0.55242847	-1.09993656
C	-6.58129916	-0.37336216	-0.20354183
H	-6.80073890	-1.01021837	0.65818253
H	-6.83210970	-0.95104374	-1.09694353
C	-7.41775132	0.87856818	-0.17889200
C	-7.79798216	1.55187653	0.91254161
C	-8.61404616	2.81255904	0.81600703
H	-9.55841392	2.70068828	1.35880342
H	-8.08252414	3.65044996	1.27879002
H	-8.83702967	3.07140628	-0.22039676
C	-3.34753691	-1.51575867	0.78619665
H	-3.30067006	-0.77929319	1.58869450
H	-7.70376444	1.28359803	-1.14843947
C	-4.38976024	-2.19225430	-1.40667347
H	-4.44637385	-1.59781120	-2.32419783
H	-3.58557450	-2.91775592	-1.51861488
H	-5.33234955	-2.74360902	-1.33013660
C	-7.44668094	1.14204695	2.31700493
H	-6.85472107	0.22824783	2.36230174
H	-6.87882464	1.94105456	2.80501112
H	-8.35621645	0.99819453	2.90946716

cannabigerol anion C3′

Charge=-1, Multiplicity=1

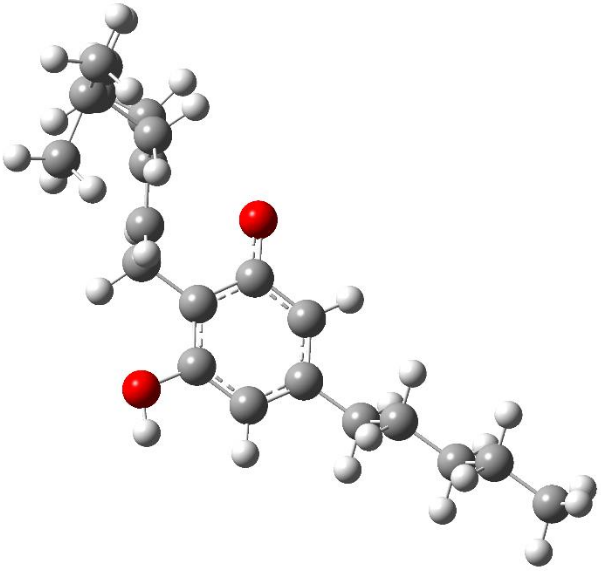

C	-1.23413236	1.52362819	1.82453550
C	0.09850564	1.92588619	1.93114850
C	0.86416864	2.29326319	0.82842250
C	0.27860764	2.23284219	-0.47450550
C	-1.08188336	1.82704519	-0.56146750
C	-1.82673436	1.47879719	0.55931250
H	-1.79237836	1.25221119	2.71576850
H	-1.53913636	1.78905419	-1.54688050
O	0.96431264	2.54505719	-1.53738850
O	0.71034264	1.97259819	3.17257350
C	-3.24859536	0.99155119	0.41326050
H	-3.70740436	1.45456219	-0.46591850

H	-3.83452436	1.29673219	1.28630950
C	-3.32909136	-0.53335281	0.27126250
H	-2.74434836	-0.84247881	-0.60280650
H	-2.86162536	-1.00044881	1.14583350
C	-4.76306336	-1.03509581	0.13034050
H	-5.23001736	-0.55960481	-0.74090650
H	-5.34464336	-0.72125681	1.00583750
C	-4.85403636	-2.55202081	-0.01617550
H	-4.27456836	-2.86326081	-0.89205550
H	-4.38461436	-3.02547881	0.85297550
C	-6.29344436	-3.03952481	-0.15228350
H	-6.77269936	-2.59525681	-1.02921350
H	-6.34208436	-4.12573081	-0.25792950
H	-6.88253436	-2.76092081	0.72600850
H	0.06924964	1.71346319	3.84515650
C	2.31192864	2.70473219	0.97310650
H	2.52081564	3.53352919	0.29557850
H	2.48762264	3.06588819	1.98951650
C	4.14331464	1.41729019	-0.27377550
C	4.95641564	0.15054319	-0.40087950
H	4.61377064	-0.58538281	0.33255150
H	4.79751664	-0.28218981	-1.39668850
C	6.46909564	0.37367719	-0.20699550
H	6.63232364	0.83046919	0.77370950
H	6.82995764	1.08629419	-0.95271350
C	7.24303564	-0.91191681	-0.34126050
C	7.42873864	-1.82137681	0.62181150
C	8.19240164	-3.09307581	0.36930750
H	9.05430064	-3.16274881	1.04103050
H	7.56321264	-3.96562981	0.57320150
H	8.54816964	-3.15324081	-0.66056250
C	3.23389664	1.54179619	0.70032950
H	3.10172964	0.69633819	1.37625950
H	7.64947764	-1.12891481	-1.32780750
C	4.45315264	2.47923919	-1.29662450
H	4.51796264	2.02953419	-2.29266750
H	3.70604364	3.27046419	-1.32761550
H	5.42531964	2.94034419	-1.09519250
C	6.89638464	-1.67952481	2.02189650
H	6.36792664	-0.74074781	2.18658050
H	6.20834464	-2.50224781	2.24312850
H	7.71356564	-1.75153381	2.74705650
